# Supplementary material for: Polygyny is linked to accelerated birdsong evolution but not to larger song repertoires
Source: Nat Commun. 2019 Feb 21;10:884. doi: 10.1038/s41467-019-08621-3 (PMC6385279; doi:10.1038/s41467-019-08621-3)
Supplement: Supplementary file 1 — Supplementary Information [file 41467_2019_8621_MOESM1_ESM.pdf]

Supplementary Information

Polygyny is linked to accelerated birdsong evolution but not to larger song repertoires

Snyder and Creanza

Table of contents

Supplementary Figs. 1–12: Phylogenies	2
Supplementary Figure 13: Examples of stochastic character maps	14
Supplementary Figs. 14–17: Analyses of evolutionary rates	15
Supplementary Figs. 18–31: Analyses of correlated evolution	22
Supplementary Figs. 32–35: Analyses with different phylogenies	45
Supplementary Figs. 36–39: Analyses of interactions between Mating system and EPP	57
Supplementary Figs. 40–41: Visualizing the distributions of data and residuals	61
Supplementary Figs. 42–47: Alternative visualizations of tests of correlated evolution	63
Supplementary Tables	69

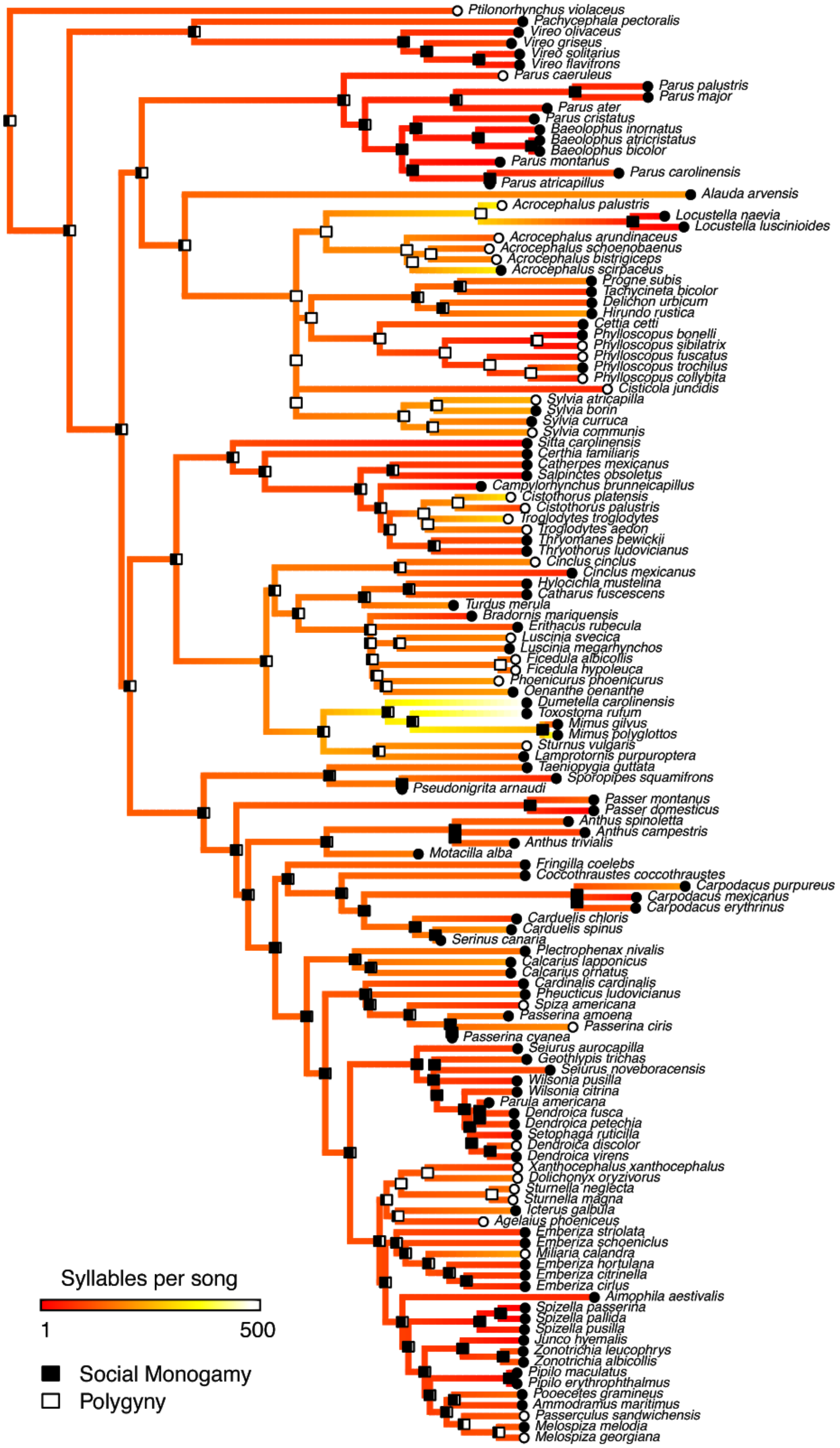

Supplementary Figure 1: Ancestral character estimation of polygyny and syllables per song. Tip labels: monogamy (black circles) and polygyny (white circles). Nodes: bars indicate the results of an ancestral character estimation algorithm (black/white: percent likelihood that the ancestor at that node was monogamous/ polygynous). The colors along the branches of the tree indicate the estimated ancestral number of syllables per song. The number of syllables per song ranged from 1 to 500 in these species and were $\log_{10}$ transformed for analysis. Monogamous and polygynous species did not have significantly different numbers of syllables per song (PhylANOVA p = 0.0597).

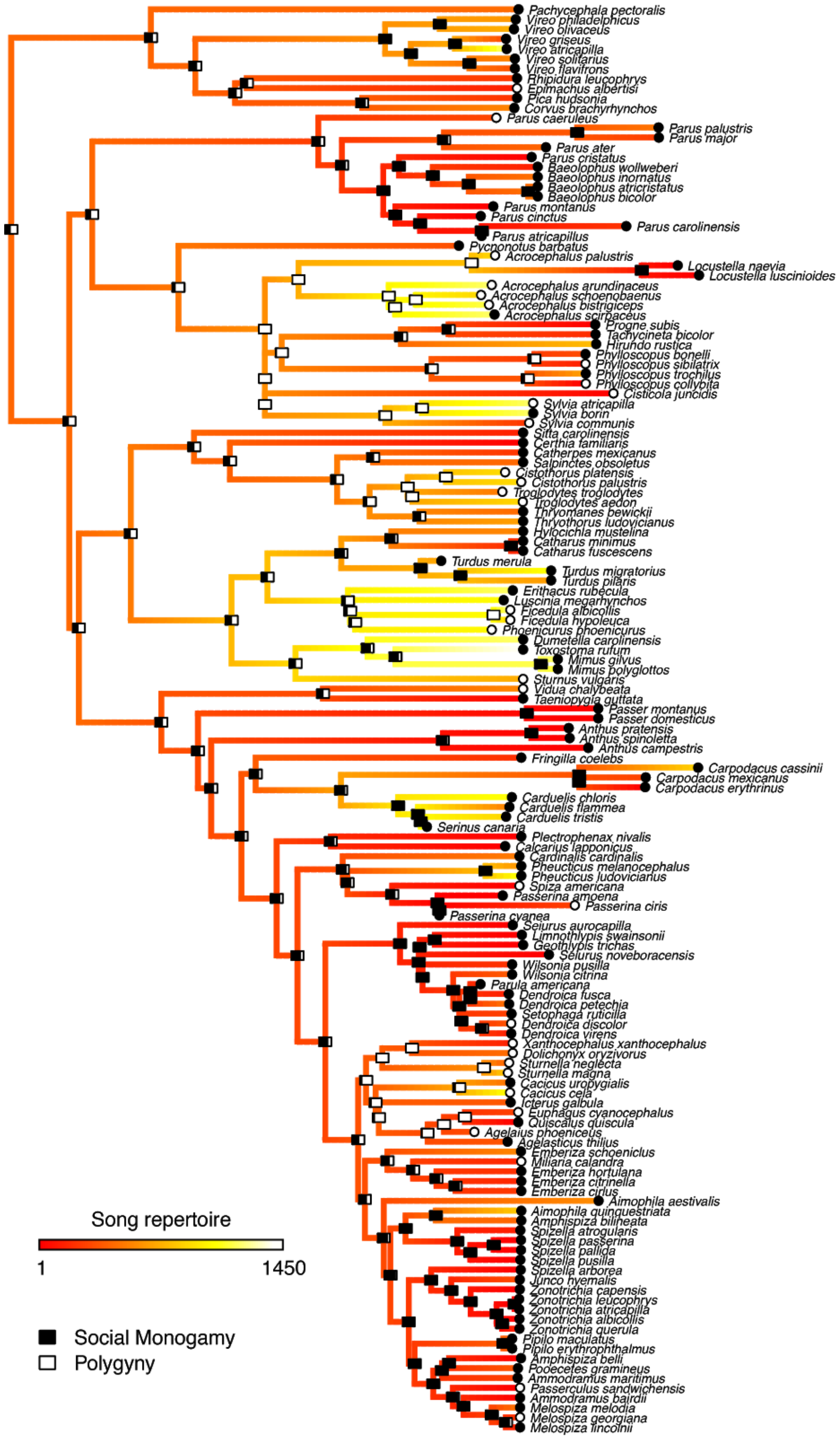

Supplementary Figure 2: Ancestral character estimation of polygyny and song repertoire size. Tip labels: monogamy (black circles) and polygyny (white circles). Nodes: bars indicate the results of an ancestral character estimation algorithm (black/white: percent likelihood that the ancestor at that node was monogamous/ polygynous). The colors along the branches of the tree indicate the estimated ancestral song repertoire size. The song repertoire size ranged from 1 to 1450 in these species and were $\log_{10}$ transformed for analysis. Monogamous and polygynous species did not have significantly different song repertoire sizes (PhylANOVA p = 0.063).

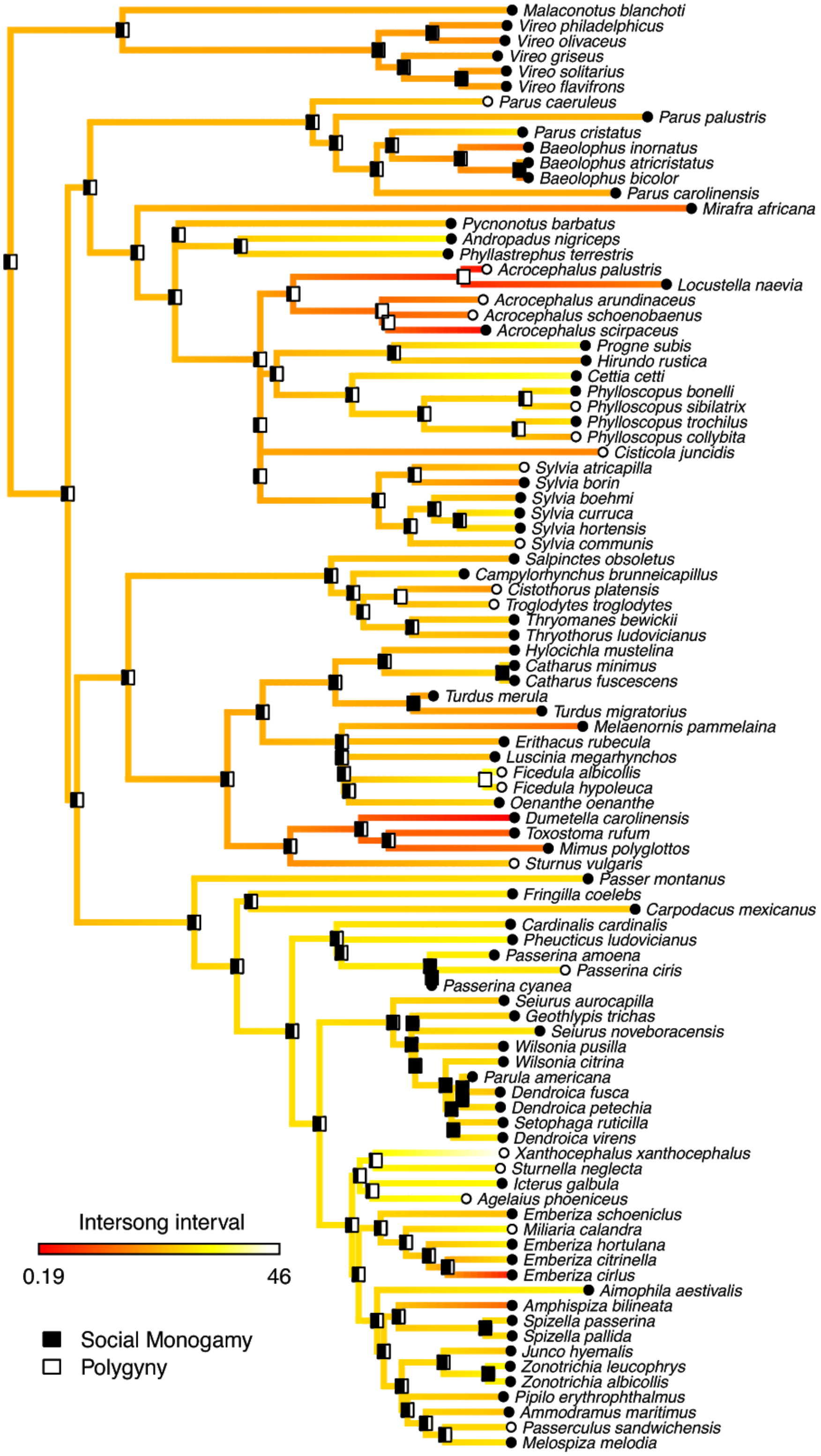

Supplementary Figure 3: Ancestral character estimation of polygyny and intersong interval. Tip labels: monogamy (black circles) and polygyny (white circles). Nodes: bars indicate the results of an ancestral character estimation algorithm (black/white: percent likelihood that the ancestor at that node was monogamous/ polygynous). The colors along the branches of the tree indicate the estimated ancestral intersong interval. The intersong interval ranged from 0.19 to 46 in these species and were log_{10} transformed for analysis. Monogamous and polygynous species did not have significantly different intersong intervals (PhylANOVA p = 0.579).

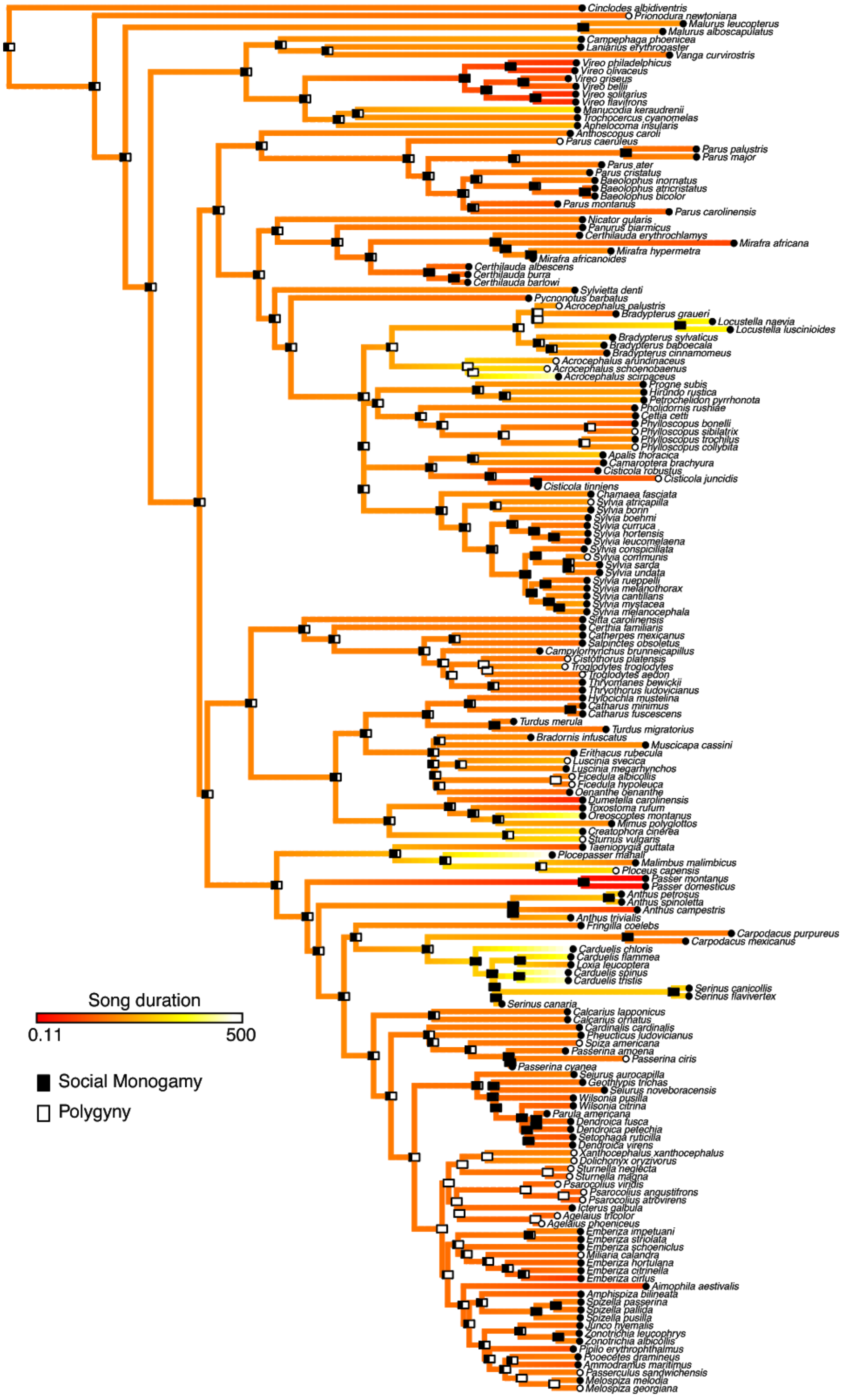

Supplementary Figure 4: Ancestral character estimation of polygyny and song duration. Tip labels: monogamy (black circles) and polygyny (white circles). Nodes: bars indicate the results of an ancestral character estimation algorithm (black/white: percent likelihood that the ancestor at that node was monogamous/ polygynous). The colors along the branches of the tree indicate the estimated ancestral song duration. The song duration ranged from 0.11 to 500 in these species and were $\log_{10}$ transformed for analysis. Monogamous and polygynous species did not have significantly different song durations (PhylANOVA p = 0.899).

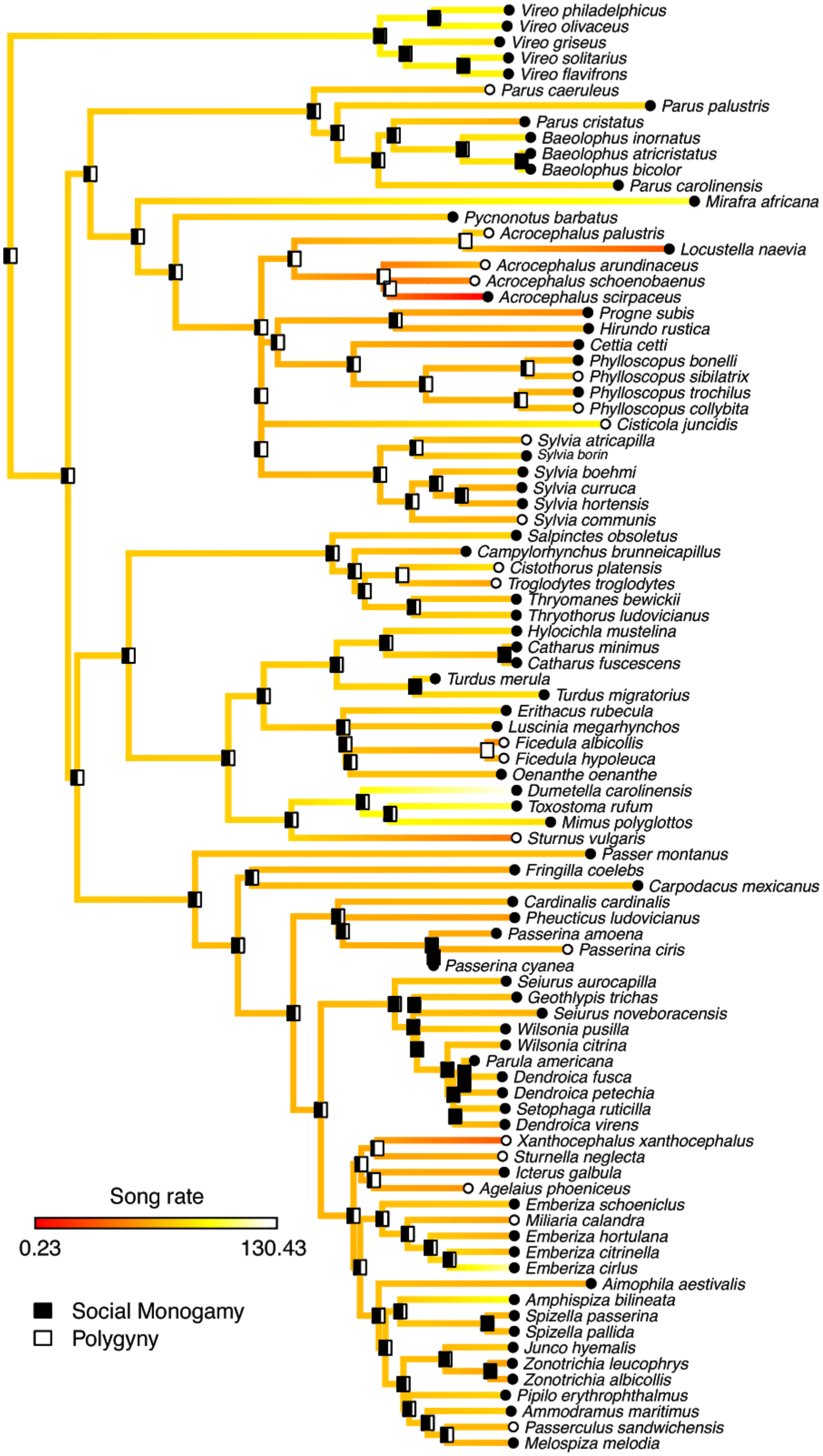

Supplementary Figure 5: Ancestral character estimation of polygyny and song rate. Tip labels: monogamy (black circles) and polygyny (white circles). Nodes: bars indicate the results of an ancestral character estimation algorithm (black/white: percent likelihood that the ancestor at that node was monogamous/ polygynous). The colors along the branches of the tree indicate the estimated ancestral song rate. The song rate ranged from 0.23 to 130.43 in these species and were log_{10} transformed for analysis. Monogamous and polygynous species did not have significantly different song rates (PhylANOVA $p = 0.0698$).

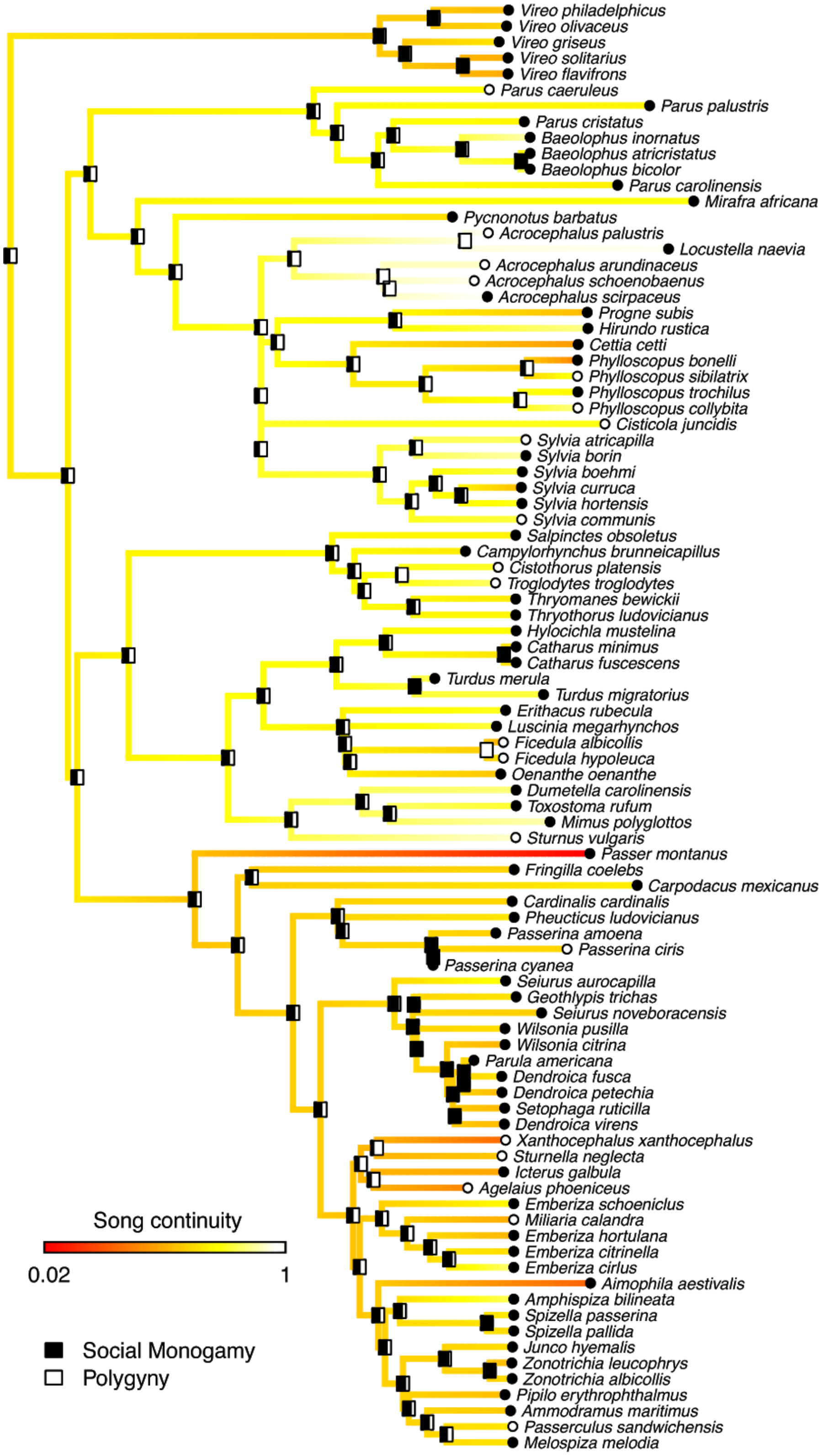

Supplementary Figure 6: Ancestral character estimation of polygyny and song continuity. Tip labels: monogamy (black circles) and polygyny (white circles). Nodes: bars indicate the results of an ancestral character estimation algorithm (black/white: percent likelihood that the ancestor at that node was monogamous/ polygynous). The colors along the branches of the tree indicate the estimated ancestral song continuity. The song continuity ranged from 0.02 to 1 in these species and were $\log_{10}$ transformed for analysis. Monogamous and polygynous species did not have significantly different continuities (PhylANOVA p = 0.221).

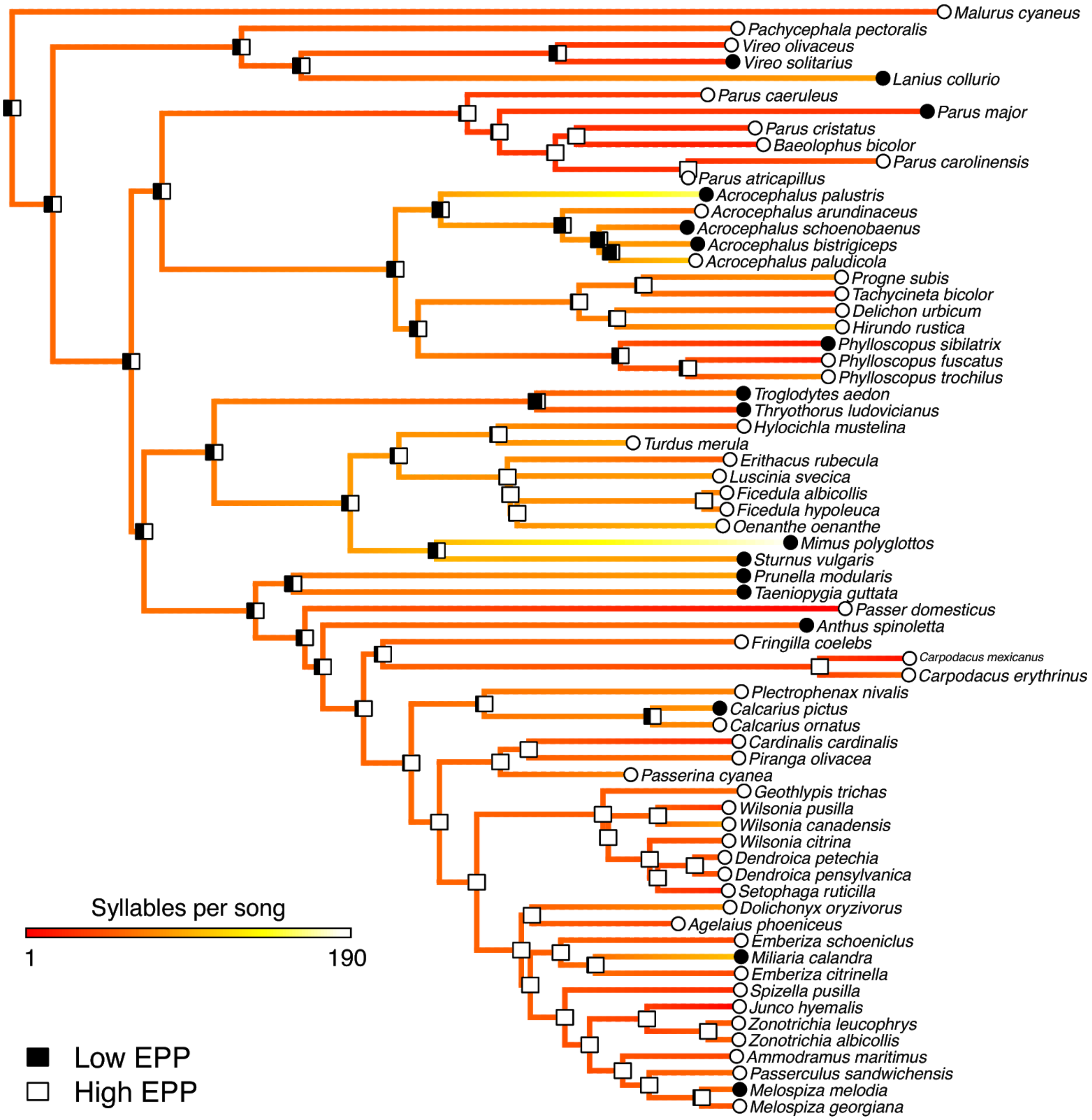

Supplementary Figure 7: Ancestral character estimation of extra-pair paternity and syllables per song. Tip labels: low rates of EPP (black circles) and high rates of EPP (white circles). Nodes: bars indicate the results of an ancestral character estimation algorithm (black/white: percent likelihood that the ancestor at that node had low EPP/high EPP). The colors along the branches of the tree indicate the estimated ancestral number of syllables per song. The number of syllables per song ranged from 1 to 190 in these species and were $\log_{10}$ transformed for analysis. Species with high versus low rates of EPP did not have significantly different numbers of syllables per song (PhylANOVA p = 0.020).

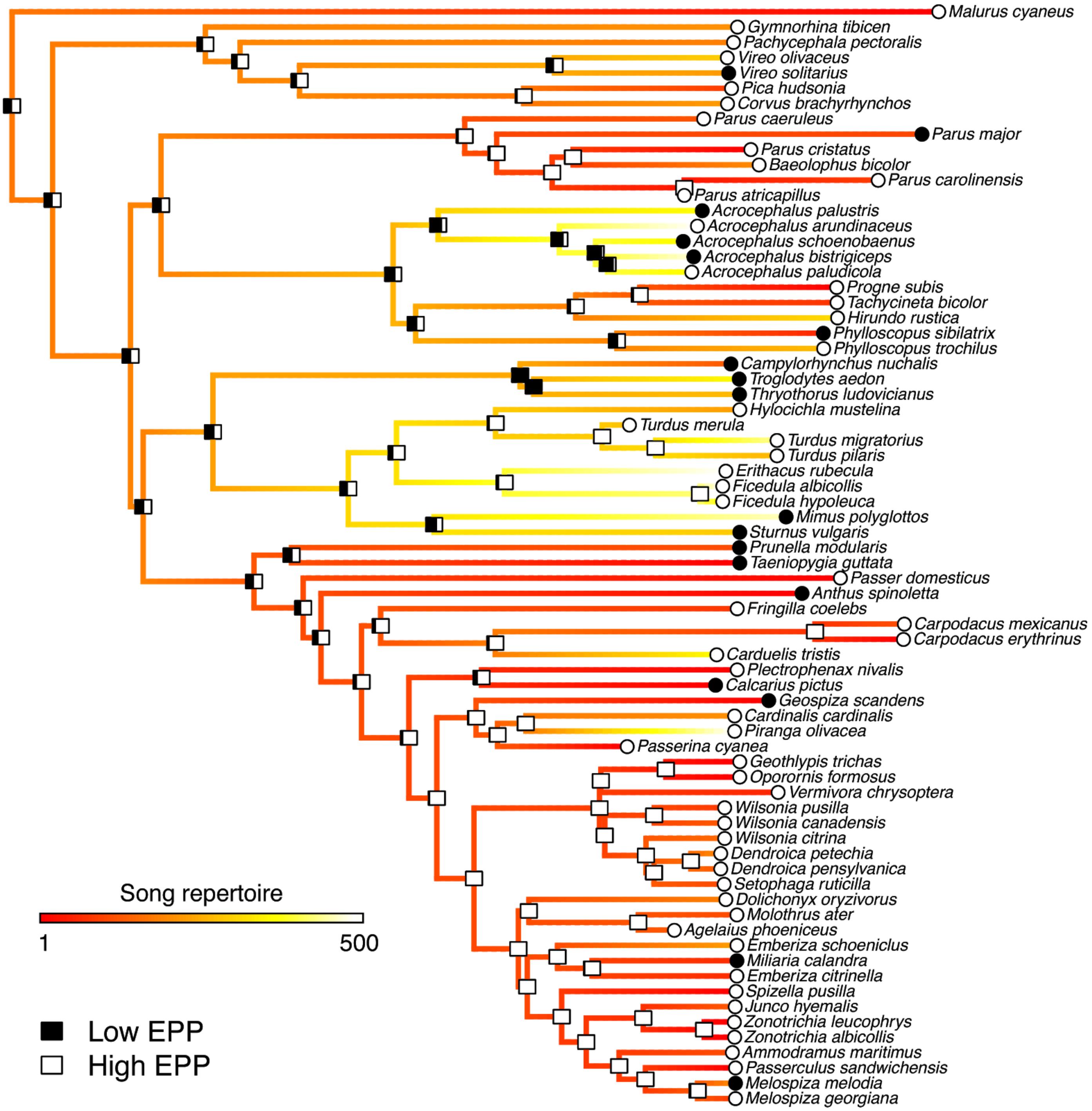

Supplementary Figure 8: Ancestral character estimation of extra-pair paternity and song repertoire size. Tip labels: low rates of EPP (black circles) and high rates of EPP (white circles). Nodes: bars indicate the results of an ancestral character estimation algorithm (black/white: percent likelihood that the ancestor at that node had low EPP/high EPP). The colors along the branches of the tree indicate the estimated ancestral song repertoire size. The song repertoire size ranged from 1 to 500 in these species and were $\log_{10}$ transformed for analysis. Species with high versus low rates of EPP did not have significantly different song repertoire sizes (PhylANOVA $p = 0.566$).

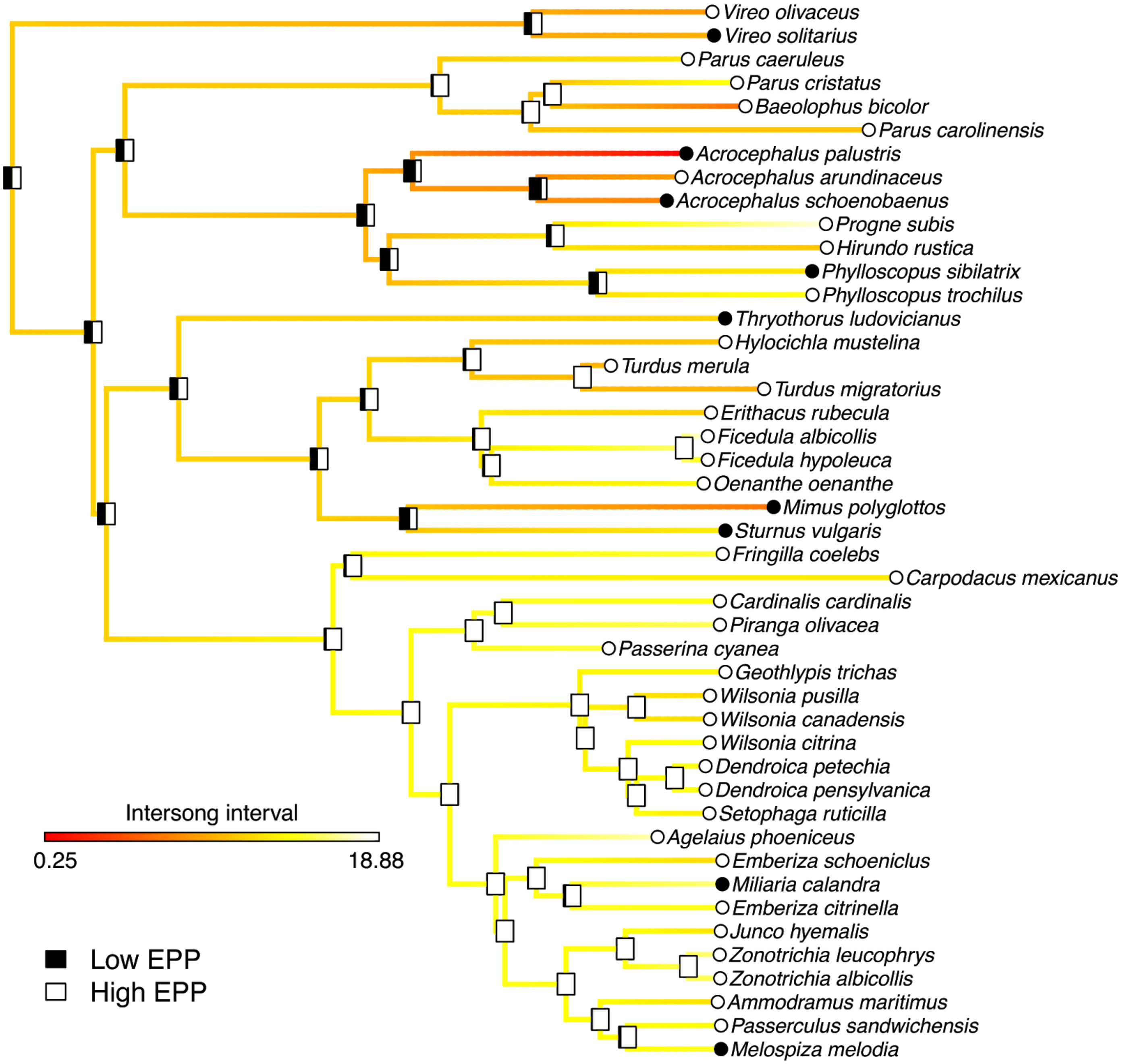

Supplementary Figure 9: Ancestral character estimation of extra-pair paternity and intersong interval. Tip labels: low rates of EPP (black circles) and high rates of EPP (white circles). Nodes: bars indicate the results of an ancestral character estimation algorithm (black/white: percent likelihood that the ancestor at that node had low EPP/high EPP). The colors along the branches of the tree indicate the estimated ancestral intersong interval. The intersong interval ranged from 0.25 to 18.88 in these species and were $\log_{10}$ transformed for analysis. Species with high versus low rates of EPP did not have significantly different intersong intervals (PhylANOVA *p* = 0.045).

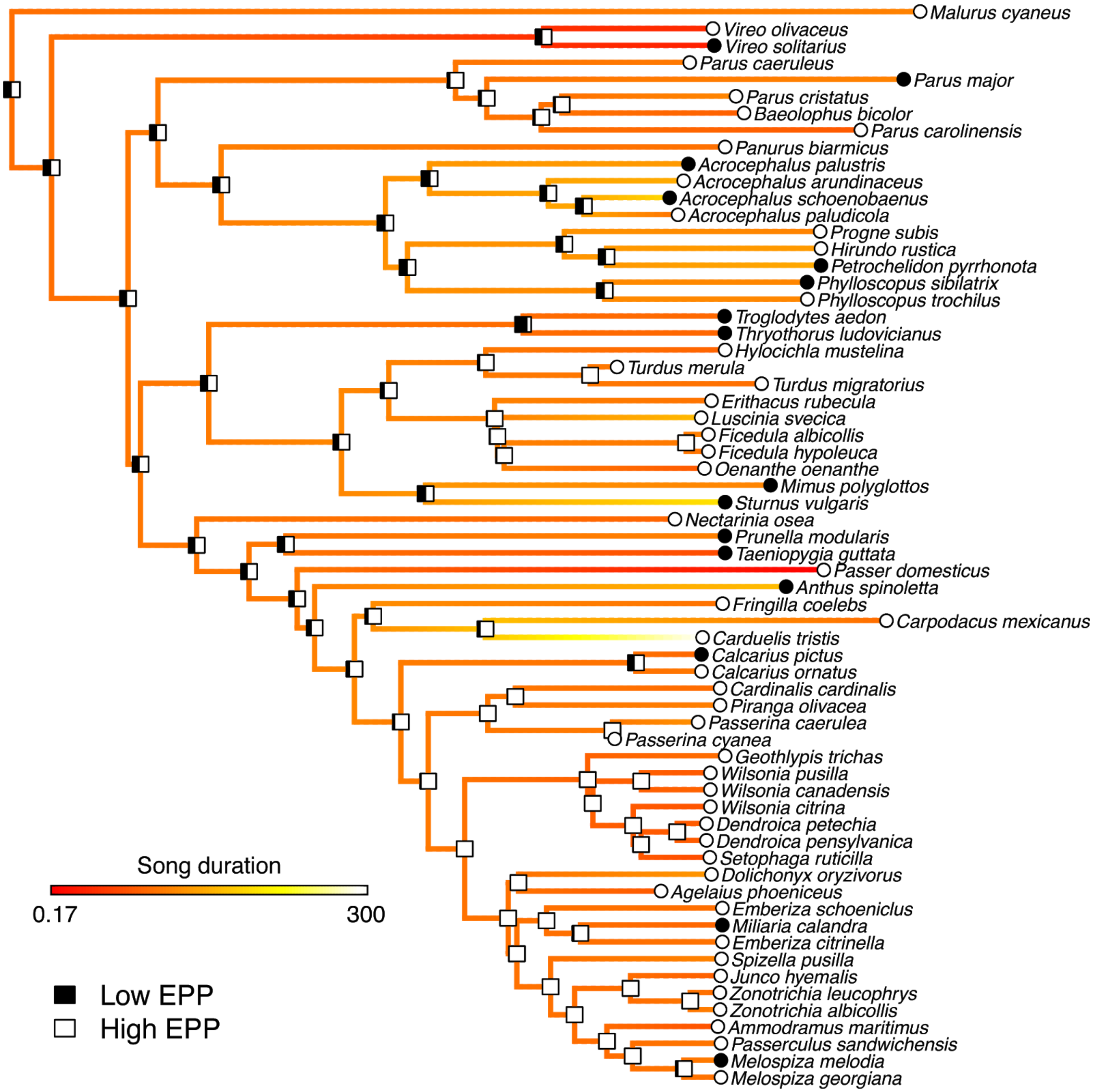

Supplementary Figure 10: Ancestral character estimation of extra-pair paternity and song duration. Tip labels: low rates of EPP (black circles) and high rates of EPP (white circles). Nodes: bars indicate the results of an ancestral character estimation algorithm (black/white: percent likelihood that the ancestor at that node had low EPP/high EPP). The colors along the branches of the tree indicate the estimated ancestral song duration. The song duration ranged from 0.17 to 300 in these species and were $\log_{10}$ transformed for analysis. Species with high versus low rates of EPP did not have significantly different song durations (PhylANOVA p = 0.329).

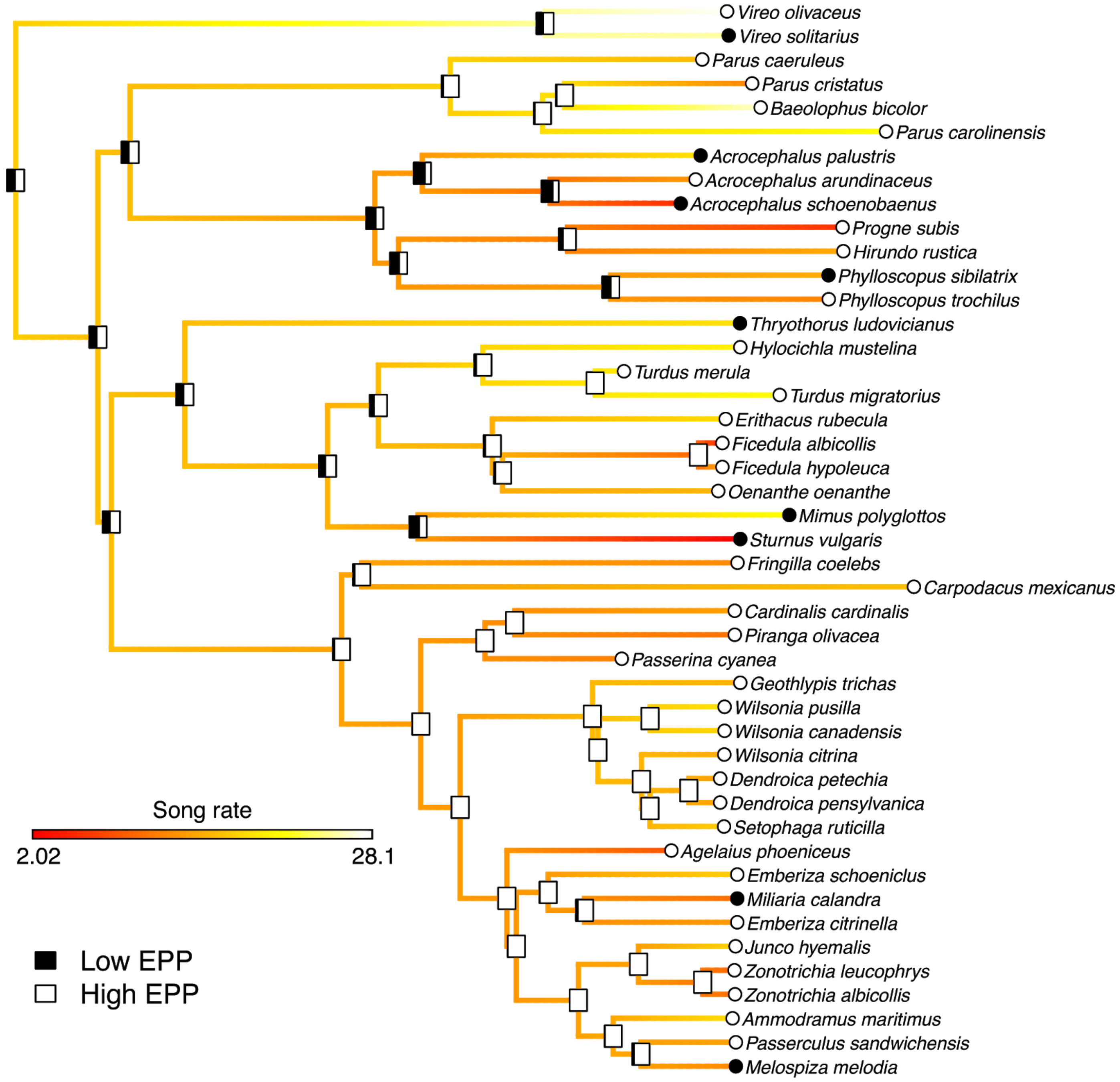

Supplementary Figure 11: Ancestral character estimation of extra-pair paternity and song rate. Tip labels: low rates of EPP (black circles) and high rates of EPP (white circles). Nodes: bars indicate the results of an ancestral character estimation algorithm (black/white: percent likelihood that the ancestor at that node had low EPP/high EPP). The colors along the branches of the tree indicate the estimated ancestral song rate. The song rate ranged from 2.02 to 28.1 in these species and were $\log_{10}$ transformed for analysis. Species with high versus low rates of EPP did not have significantly different song rates (PhylANOVA p = 0.714).

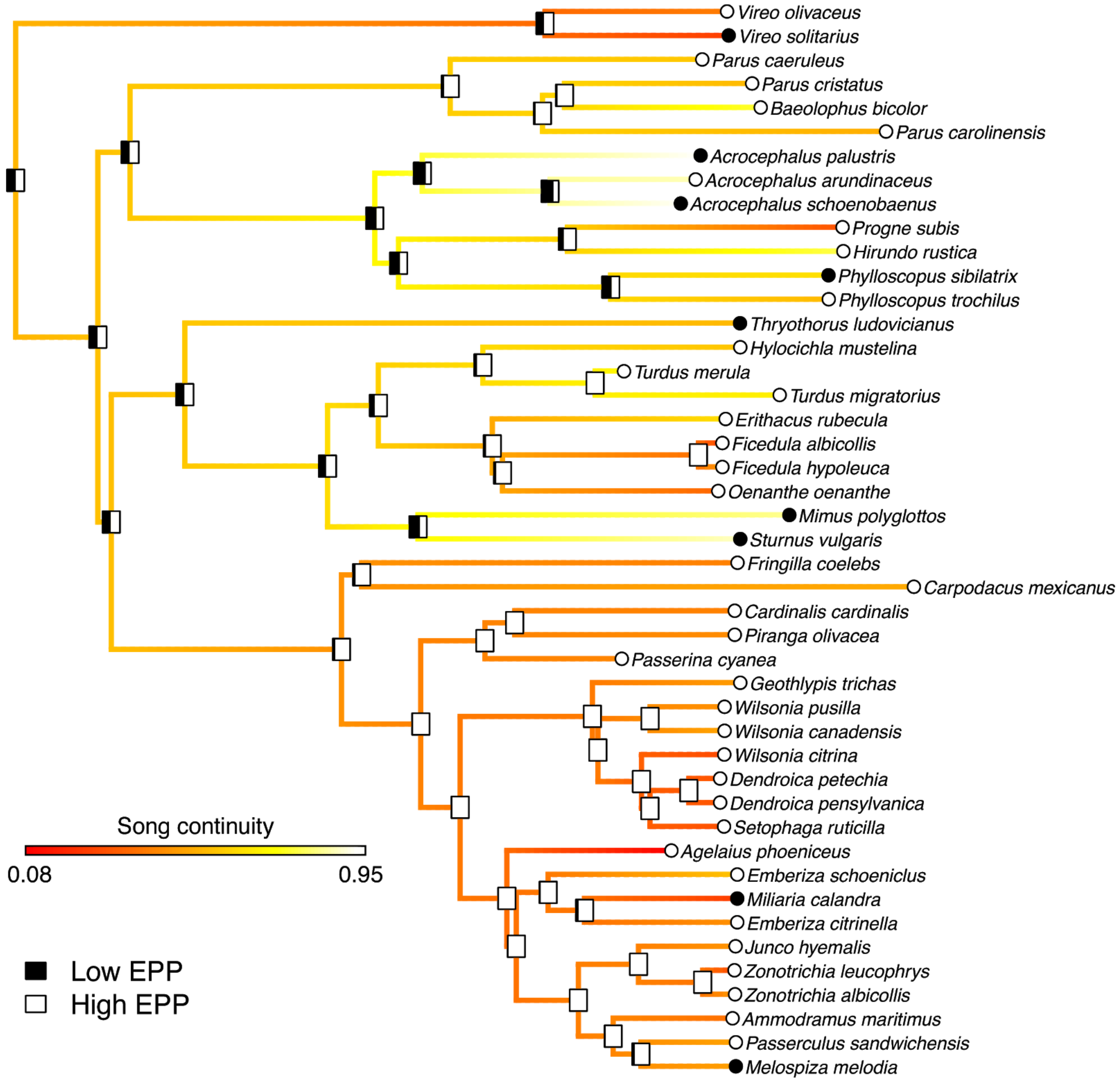

Supplementary Figure 12: Ancestral character estimation of extra-pair paternity and song continuity. Tip labels: low rates of EPP (black circles) and high rates of EPP (white circles). Nodes: bars indicate the results of an ancestral character estimation algorithm (black/white: percent likelihood that the ancestor at that node had low EPP/high EPP). The colors along the branches of the tree indicate the estimated ancestral song continuity. The song continuity ranged from 0.08 to 0.95 in these species and were $\log_{10}$ transformed for analysis. Species with high versus low rates of EPP did not have significantly different song continuities (PhylANOVA p = 0.052).

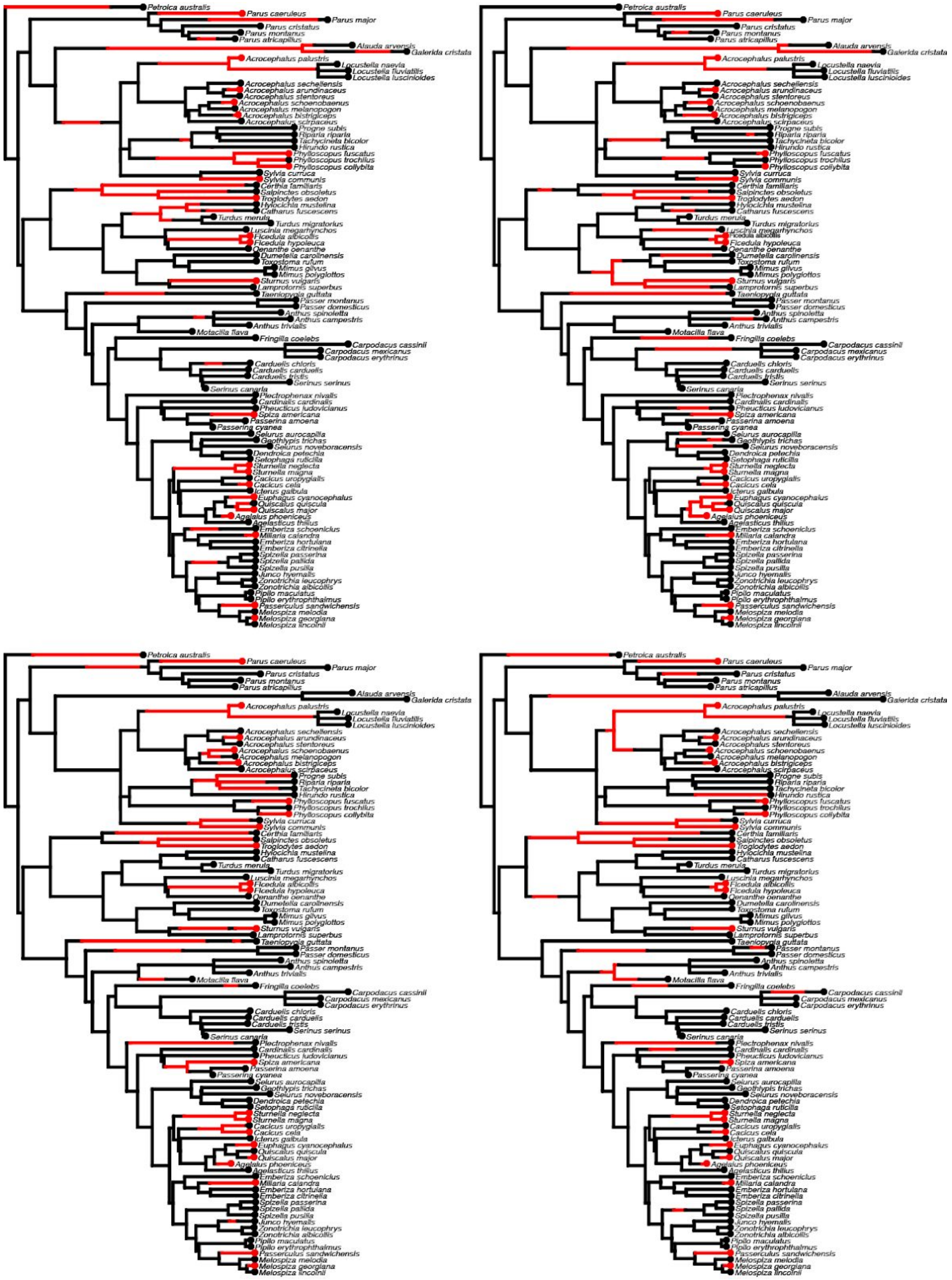

Supplementary Figure 13: Examples of stochastic character maps. We estimated the evolutionary transition rates from monogamy to polygyny and from polygyny to monogamy. Using these rates, we simulated possible evolutionary histories in which the ancestral states of monogamy (black) and polygyny (red) are consistent with these estimated rates of evolution. Four examples of such stochastic character maps, also called simmaps, are shown here; we tested 1000 simmaps for each Brownie analysis.

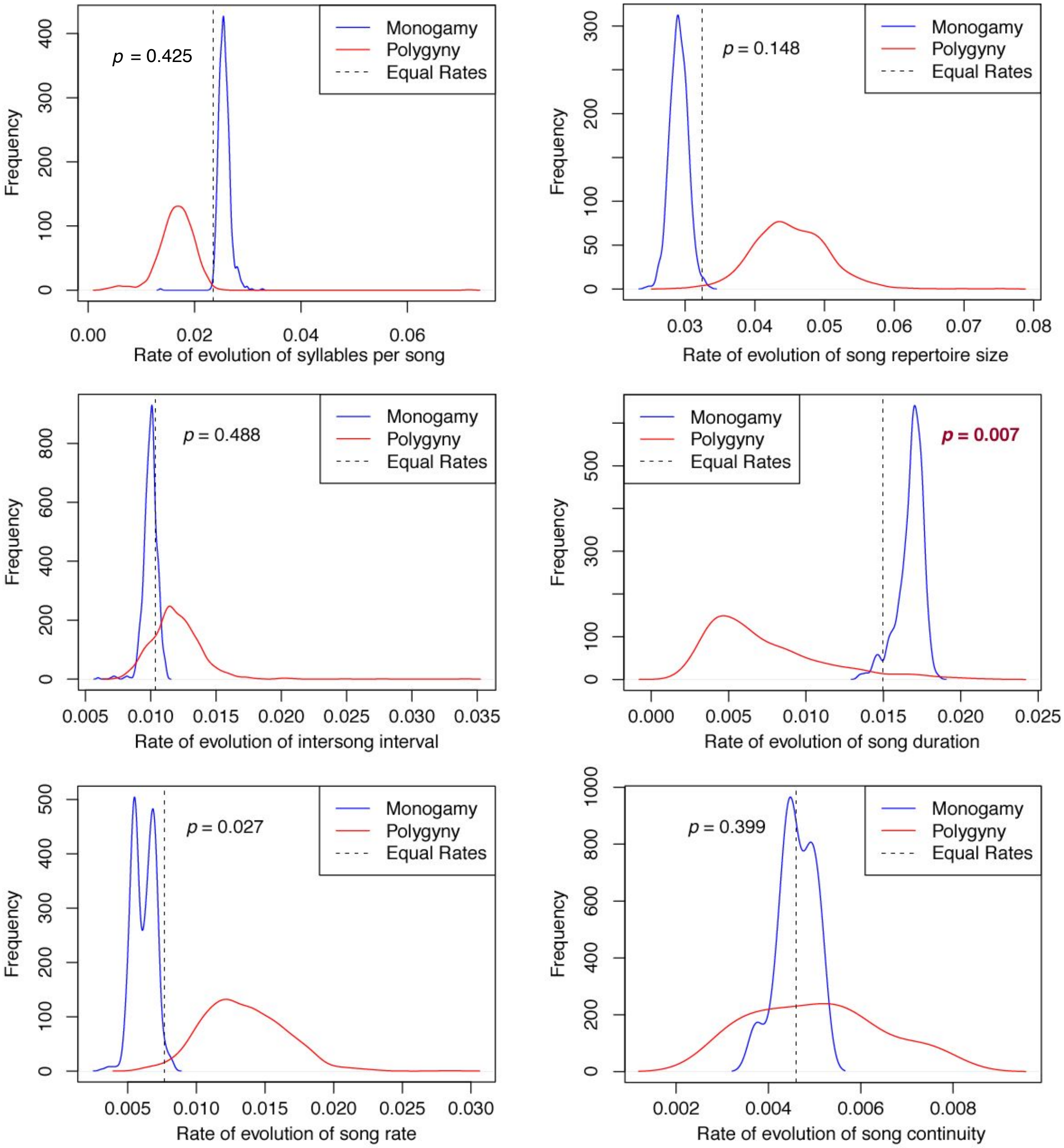

Supplementary Figure 14: Analyses of the rate of evolution of song characteristics in monogamous versus polygynous lineages. We generated 1000 stochastic character maps of possible evolutionary histories of monogamy and polygyny (as in Supplementary Figure 13), and then we tested whether song characteristics evolved at different rates in monogamous versus polygynous portions of the tree. From all runs that converged out of 1000 total runs of the Brownie algorithm, we plot the distribution of the rate of evolution of each song characteristic in monogamous lineages (blue) and its rate of evolution in polygynous lineages (red). Distributions are kernel density plots generated using the R function `density` with a Gaussian smoothing kernel. The dashed line indicates the rate of evolution estimated when the song characteristic is assumed to evolve at the same rate in monogamous and polygynous periods of evolutionary history. We found that syllable repertoire size evolved significantly faster in polygynous lineages (Figure 4, main text) and that song duration evolved significantly faster in monogamous lineages.

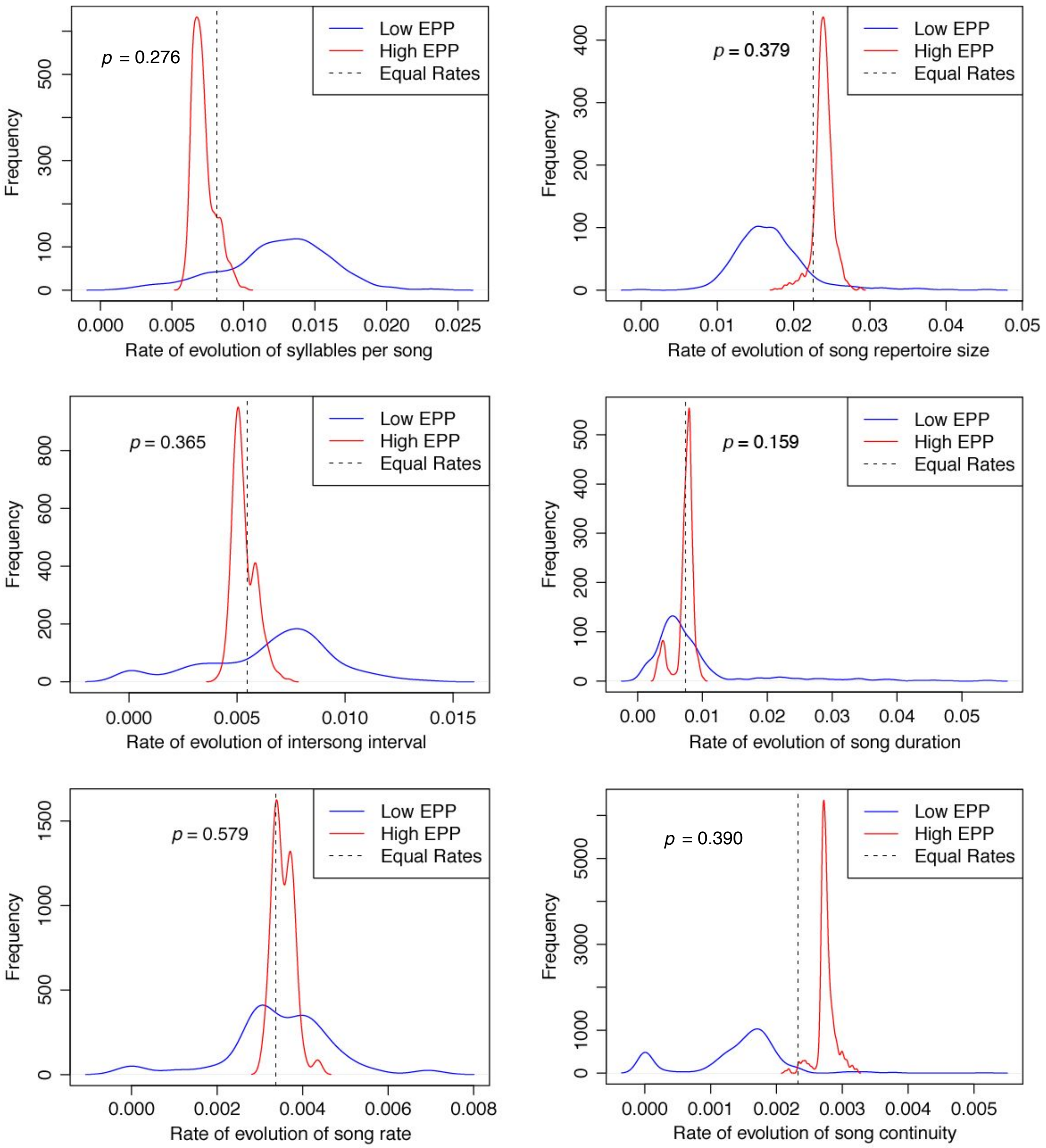

Supplementary Figure 15: Analyses of the rate of evolution of song characteristics in monogamous versus polygynous lineages. We generated 1000 stochastic character maps of possible evolutionary histories of high and low rates of EPP (as in Supplementary Figure 13), and then we tested whether song characteristics evolved at different rates in high versus low EPP lineages. From all runs that converged out of 1000 total runs of the Brownie algorithm, we plot the distribution of the rate of evolution of each song characteristic in low EPP lineages (blue) and its rate of evolution in high EPP lineages (red). Distributions are kernel density plots generated using the R function `density` with a Gaussian smoothing kernel. The dashed line indicates the rate of evolution estimated when the song characteristic is assumed to evolve at the same rate in low EPP and high EPP periods of evolutionary history. We found that high versus low extra-pair paternity did not appear to alter the rate of evolution of any studied song characteristic.

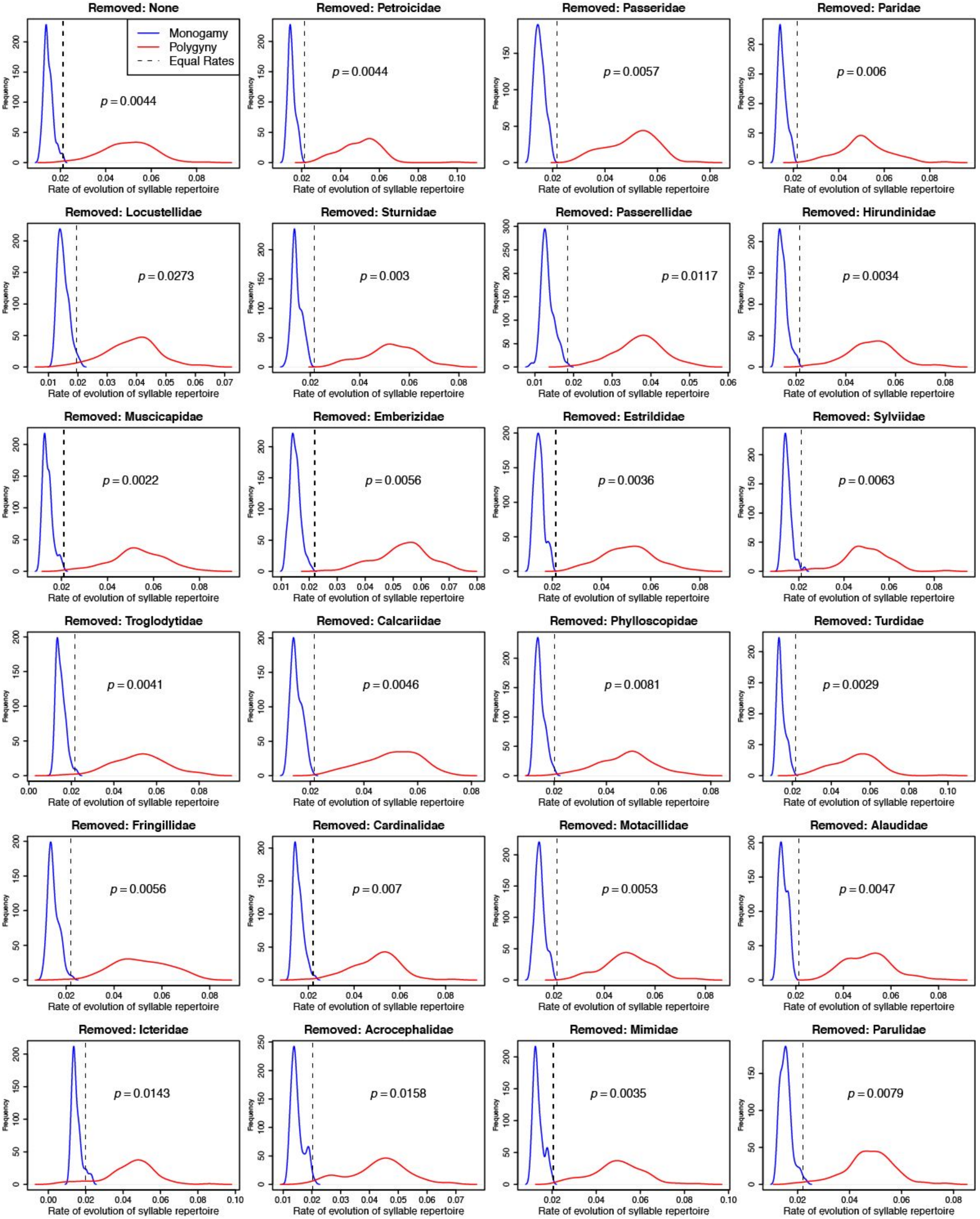

Removed: None
Monogamy
Polygyny
Equal Rates
p = 0.0044
Removed: Petroicidae
p = 0.0044
Removed: Passeridae
p = 0.0057
Removed: Paridae
p = 0.006
Removed: Locustellidae
p = 0.0273
Removed: Sturnidae
p = 0.003
Removed: Passerellidae
p = 0.0117
Removed: Hirundinidae
p = 0.0034
Removed: Muscicapidae
p = 0.0022
Removed: Emberizidae
p = 0.0056
Removed: Estrildidae
p = 0.0036
Removed: Sylviidae
p = 0.0063
Removed: Troglodytidae
p = 0.0041
Removed: Calcariidae
p = 0.0046
Removed: Phylloscopidae
p = 0.0081
Removed: Turdidae
p = 0.0029
Removed: Fringillidae
p = 0.0056
Removed: Cardinalidae
p = 0.007
Removed: Motacillidae
p = 0.0053
Removed: Alaudidae
p = 0.0047
Removed: Icteridae
p = 0.0143
Removed: Acrocephalidae
p = 0.0158
Removed: Mimidae
p = 0.0035
Removed: Parulidae
p = 0.0079
Frequency
Rate of evolution of syllable repertoire

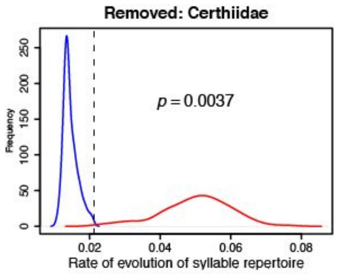

Supplementary Figure 16: Family-level jackknife resampling to test the rate of evolution of syllable repertoire size in monogamous versus polygynous lineages. (Continued from previous page.) Each family was removed in turn for the Brownie analysis comparing the rate of syllable repertoire size evolution in monogamous and polygynous branches of the phylogeny, simulated as 100 unique stochastic character maps. The family that was removed is indicated above the plot. We tested for significance by performing a likelihood-ratio test on the average log-likelihood values across the 100 runs of the Brownie algorithm. The first *p*-value indicates a test of the null hypothesis that the rate distributions are drawn from the same underlying distribution. We also tested the null hypotheses that the rate of evolution was faster in polygyny and in monogamy. We did not find any qualitative differences in the results reported in the main text (**Figure 4A**) with the removal of any family.

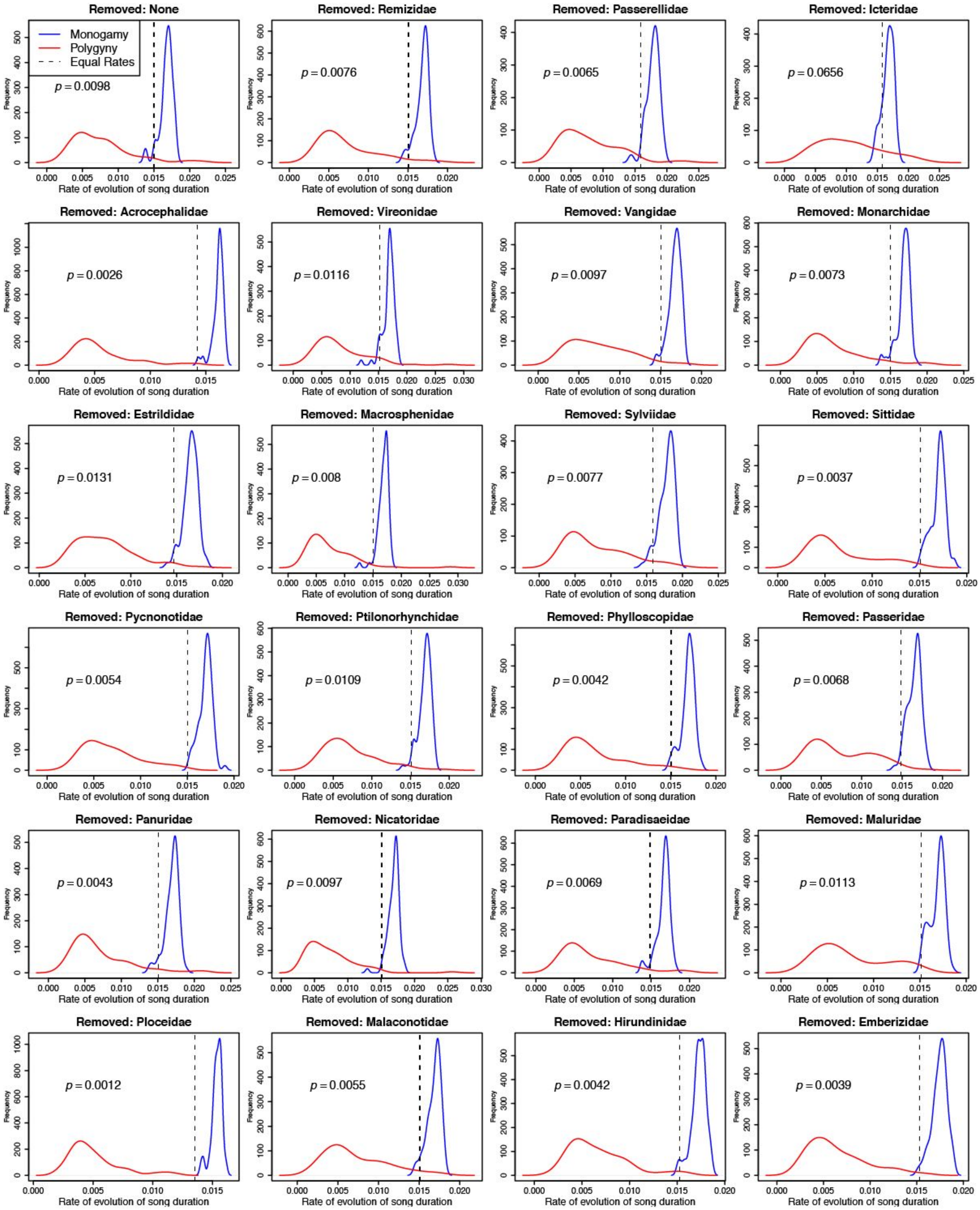
Removed: None
Monogamy
Polygyny
Equal Rates
p = 0.0098
Removed: Remizidae
p = 0.0076
Removed: Passerellidae
p = 0.0065
Removed: Icteridae
p = 0.0656
Removed: Acrocephalidae
p = 0.0026
Removed: Vireonidae
p = 0.0116
Removed: Vangidae
p = 0.0097
Removed: Monarchidae
p = 0.0073
Removed: Estrildidae
p = 0.0131
Removed: Macrosphenidae
p = 0.008
Removed: Sylviidae
p = 0.0077
Removed: Sittidae
p = 0.0037
Removed: Pycnonotidae
p = 0.0054
Removed: Ptilonorhynchidae
p = 0.0109
Removed: Phylloscopidae
p = 0.0042
Removed: Passeridae
p = 0.0068
Removed: Panuridae
p = 0.0043
Removed: Nicatoridae
p = 0.0097
Removed: Paradisaeidae
p = 0.0069
Removed: Maluridae
p = 0.0113
Removed: Ploceidae
p = 0.0012
Removed: Malaconotidae
p = 0.0055
Removed: Hirundinidae
p = 0.0042
Removed: Emberizidae
p = 0.0039
Frequency
Rate of evolution of song duration

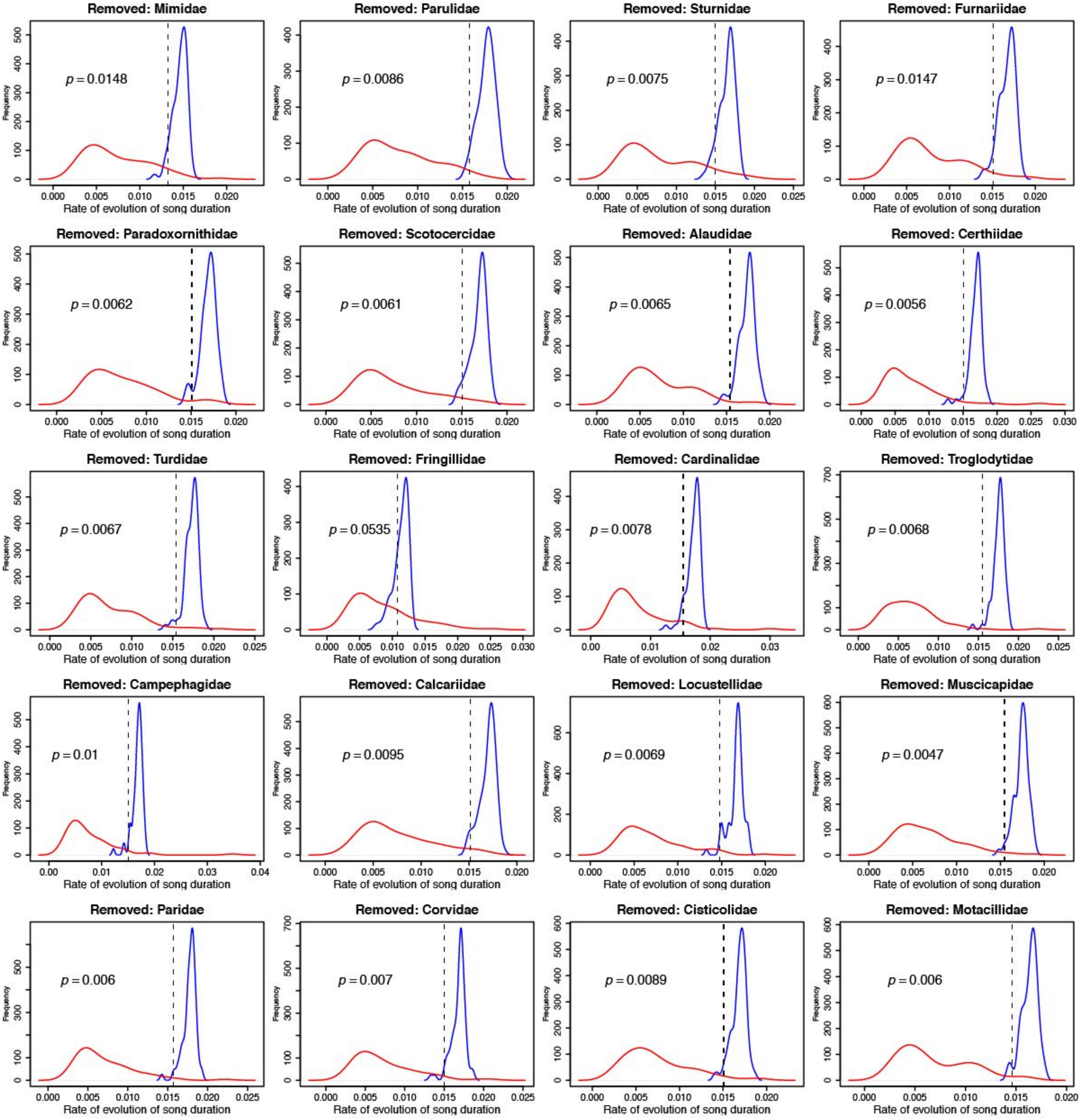

Supplementary Figure 17: Family-level jackknife resampling to test the rate of evolution of song duration in monogamous versus polygynous lineages. (Continued from previous page.) Each family was removed in turn for the Brownie analysis comparing the rate of song duration evolution in monogamous and polygynous branches of the phylogeny, simulated as 100 unique stochastic character maps. The family that was removed is indicated above the plot. We tested for significance by performing a likelihood-ratio test on the average log-likelihood values across the 100 runs of the Brownie algorithm. The first *p*-value indicates a test of the null hypothesis that the rate distributions are drawn from the same underlying distribution. We also tested the null hypotheses that the rate of evolution was faster in polygyny and in monogamy. We did not find any qualitative differences in the results reported in the main text (**Figure 4B**) with the removal of any family.

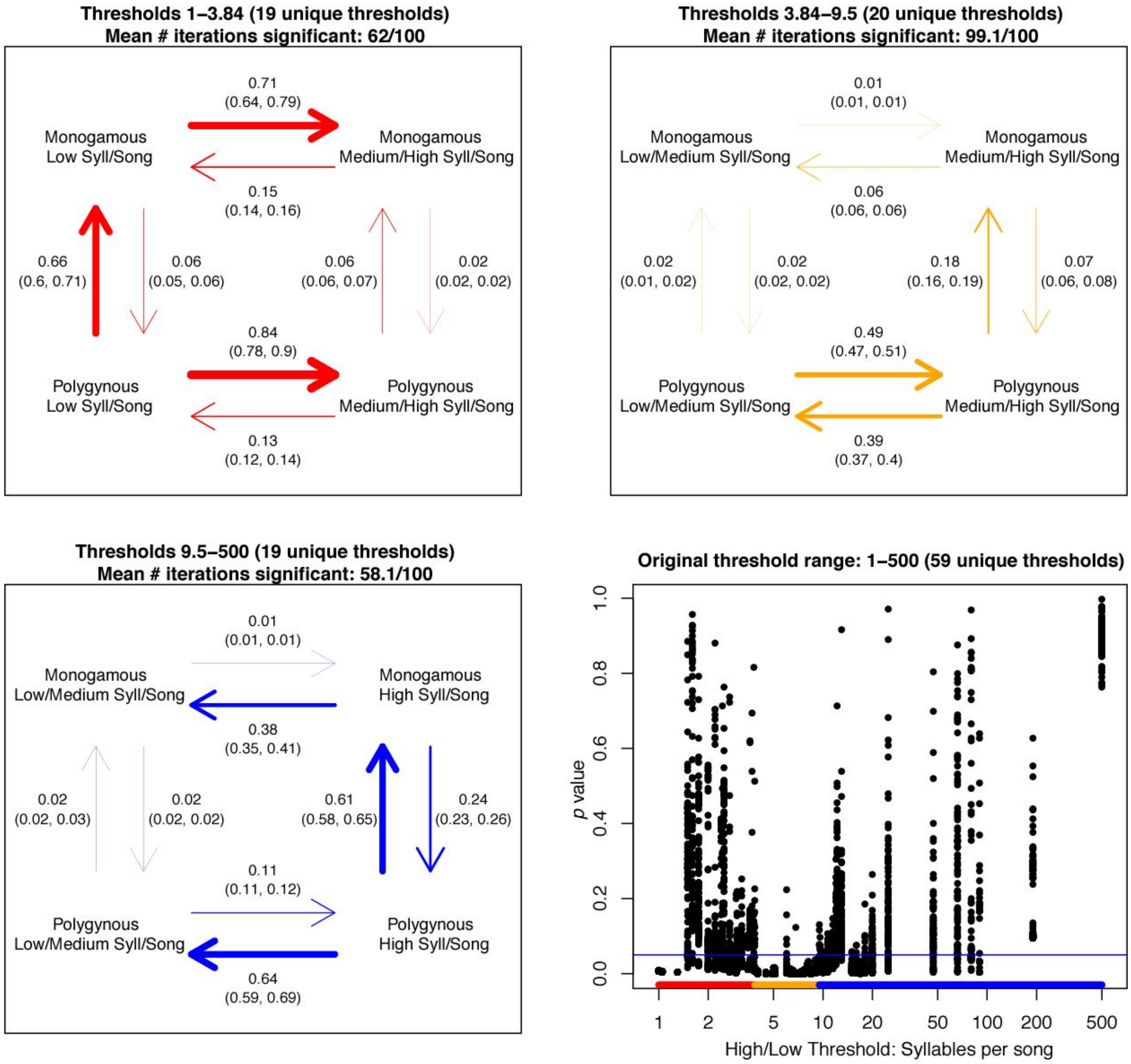

Supplementary Figure 18: Detecting correlated evolution of mating systems and syllables per song. We tested the correlated evolution of number of syllables per song and polygyny using BayesTraits, with syllables per song made binary based on a threshold delineating smaller versus larger numbers of syllables per song. Each observed value of syllables per song was used as the threshold for 100 runs of BayesTraits. Figure panels follow the same format as **Figures 5–6** in the main text. We generated transition plots by calculating the mean rate and 95% confidence interval (in parentheses) for each transition rate, shown here when the threshold between low and high syllables per song is in the lowest third of observed values (red arrows), the middle third of observed values (yellow arrows), and the highest third of observed values (blue arrows). The bottom right panel shows the likelihood-ratio test *p*-value for each run of BayesTraits at each threshold value. These results for syllables per song are qualitatively similar to our results for syllable repertoire size (**Figure 5**). The combination of polygyny and very small numbers of syllables per song is unstable, with high rates of transition away from this state (red arrows). Similarly, the combination of polygyny with very large numbers of syllables per song is unstable (blue arrows). Monogamy also appears to be unstable with either extreme. In the middle range of threshold values (yellow arrows), polygyny significantly accelerates the rate of evolution of syllables per song.

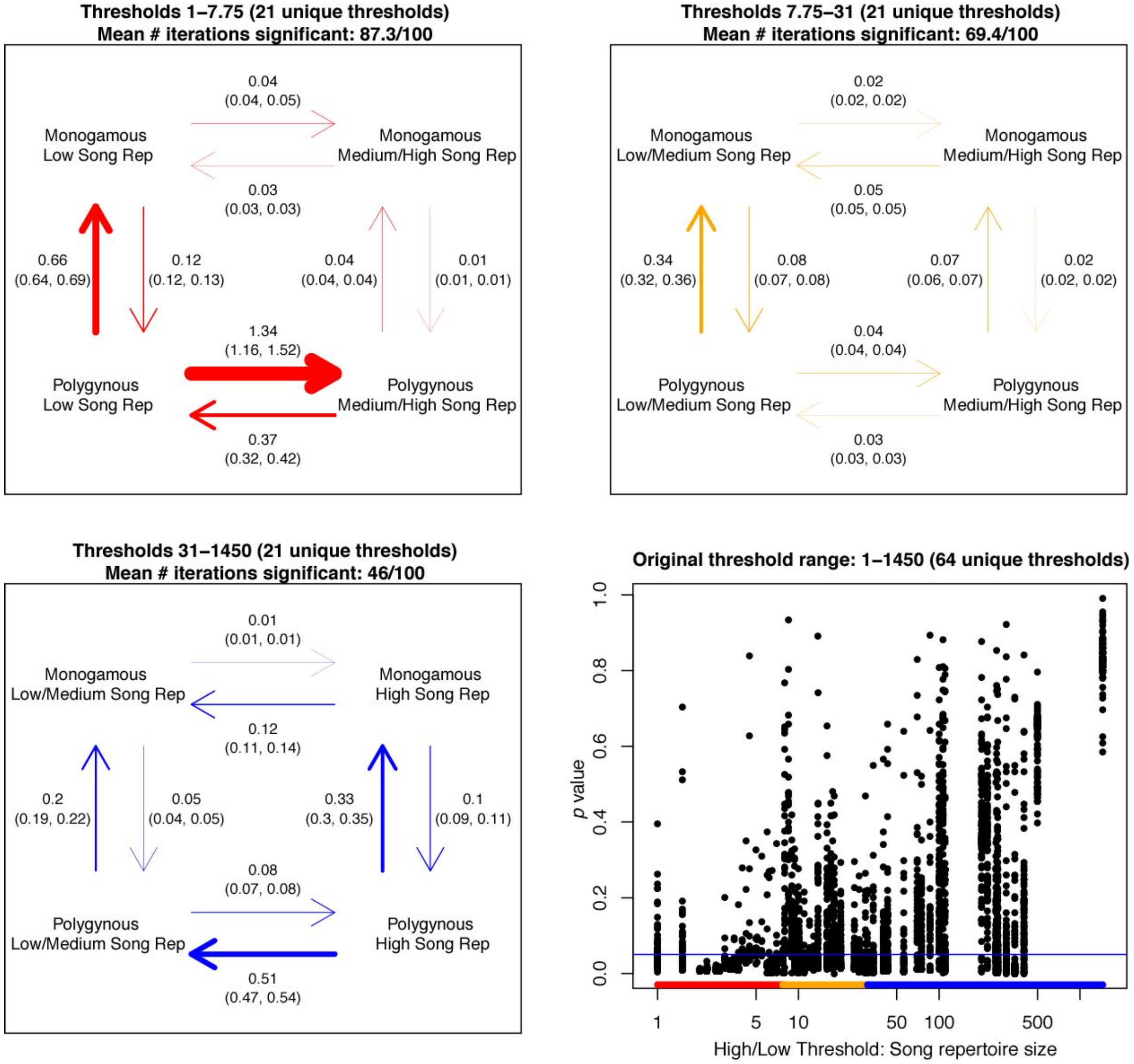

Supplementary Figure 19: Detecting correlated evolution of mating systems and song repertoire size. We tested the correlated evolution of song repertoire size and polygyny using BayesTraits, with song repertoire size made binary based on a threshold delineating smaller versus larger song repertoires. Each observed value of song repertoire size was used as the threshold for 100 runs of BayesTraits. Figure panels follow the same format as **Figures 5–6** in the main text. We generated transition plots by calculating the mean rate and 95% confidence interval (in parentheses) for each transition rate, shown here when the threshold between low and high song repertoire is in the lowest third of observed values (red arrows), the middle third of observed values (yellow arrows), and the highest third of observed values (blue arrows). The bottom right panel shows the likelihood-ratio test *p*-value for each run of BayesTraits at each threshold value. These results for song repertoire size follow a similar pattern to our results for syllable repertoire size (**Figure 5**). The combination of polygyny and very small song repertoires is unstable, with high rates of transition away from this state (red arrows). Similarly, the combination of polygyny with very large song repertoires is unstable (blue arrows).

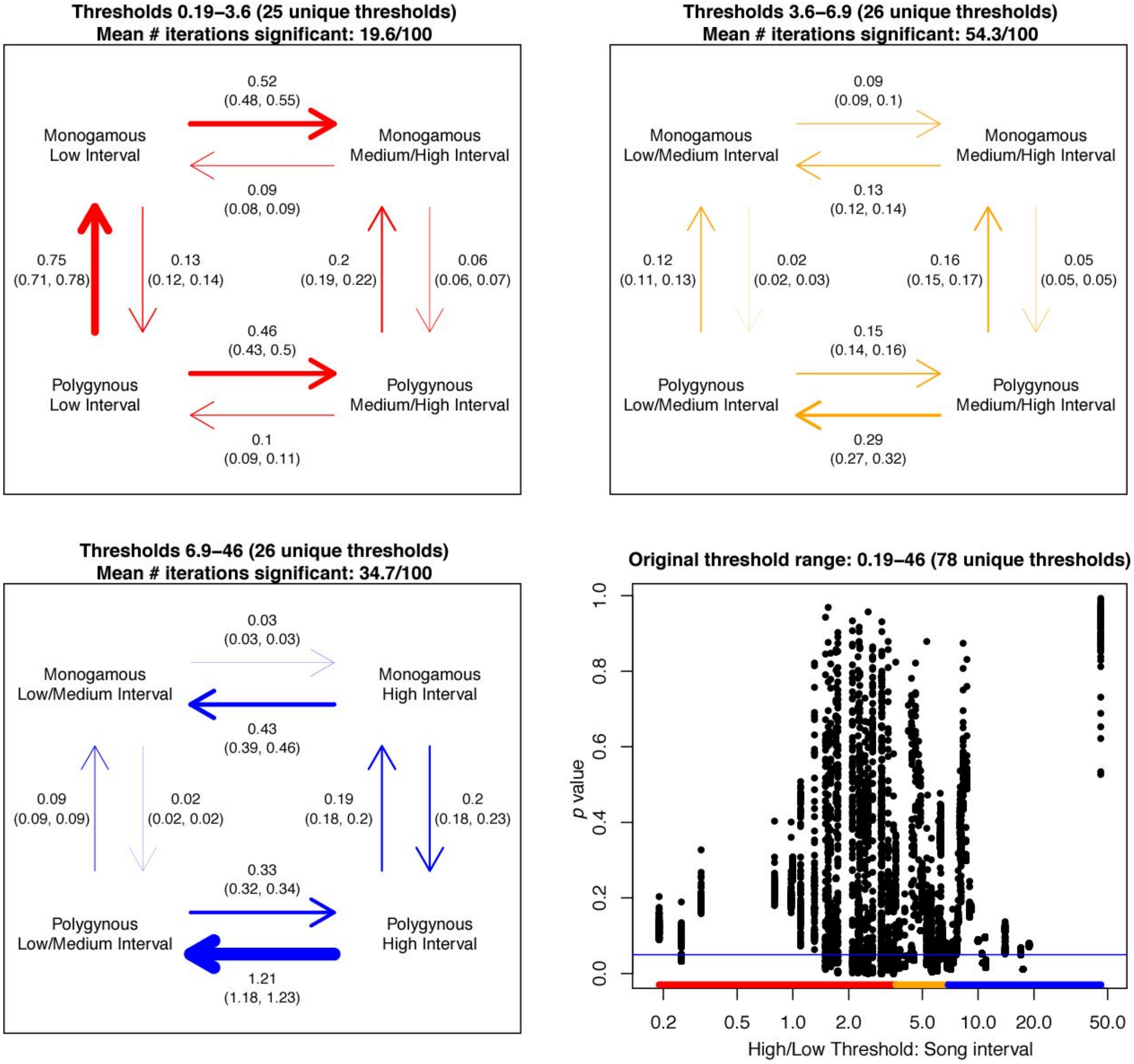

Supplementary Figure 20: Detecting correlated evolution of mating systems and intersong interval. We tested the correlated evolution of intersong interval and polygyny using BayesTraits, with intersong interval made binary based on a threshold delineating smaller versus larger intervals. Each observed value of intersong interval was used as the threshold for 100 runs of BayesTraits. Figure panels follow the same format as **Figures 5–6** in the main text. We generated transition plots by calculating the mean rate and 95% confidence interval (in parentheses) for each transition rate, shown here when the threshold between low and high intersong intervals is in the lowest third of observed values (red arrows), the middle third of observed values (yellow arrows), and the highest third of observed values (blue arrows). The bottom right panel shows the likelihood-ratio test *p*-value for each run of BayesTraits at each threshold value. These results for intersong interval follow a similar pattern to our results for syllable repertoire size (**Figure 5**). The combination of polygyny and very small intersong intervals is unstable, with high rates of transition away from this state (red arrows). Similarly, the combination of polygyny with very large intersong intervals is unstable (blue arrows).

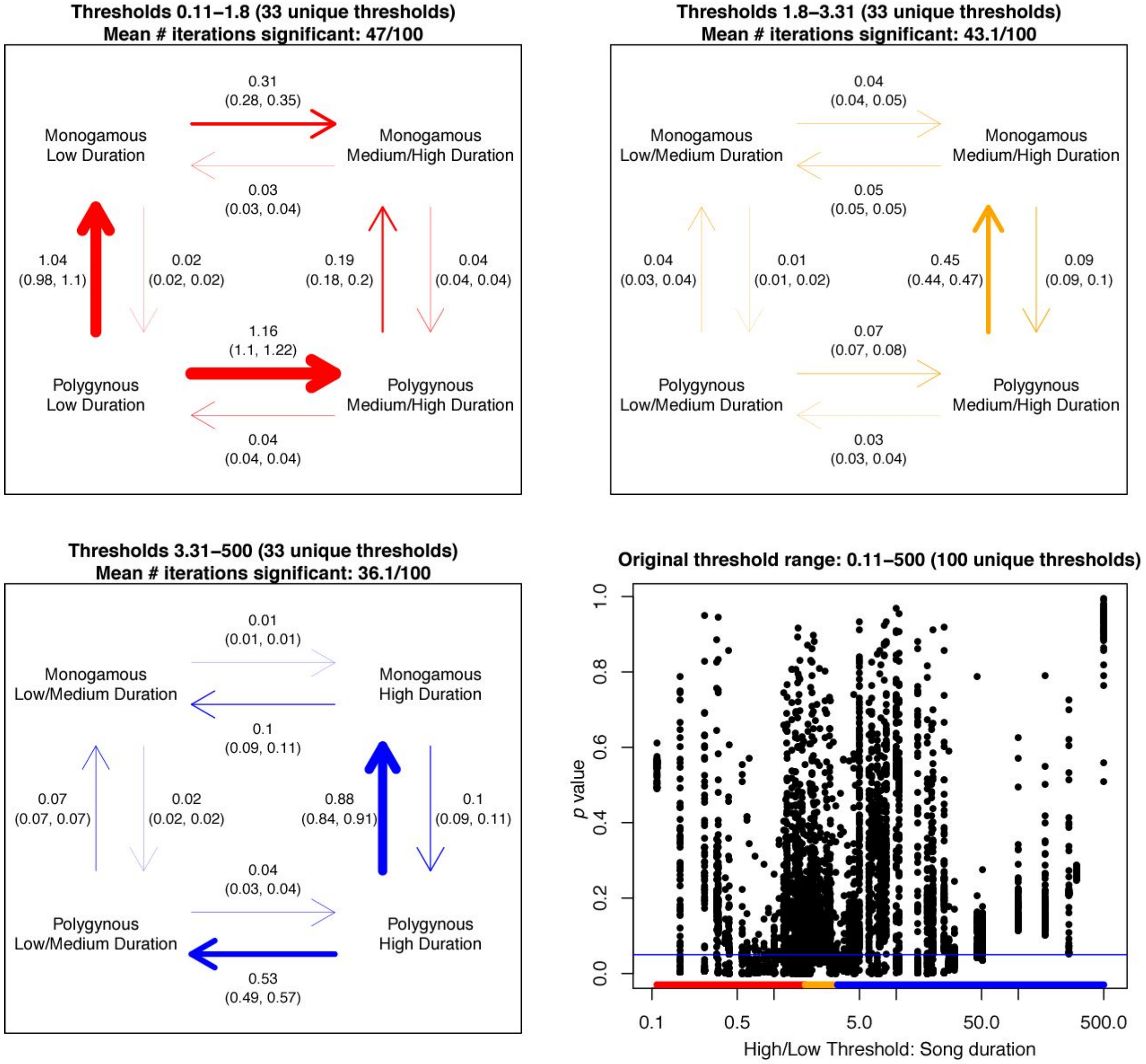

Supplementary Figure 21: Detecting correlated evolution of mating systems and song duration. We tested the correlated evolution of song duration and polygyny using BayesTraits, with song duration made binary based on a threshold delineating smaller versus larger durations. Each observed value of song duration was used as the threshold for 100 runs of BayesTraits. Figure panels follow the same format as **Figures 5–6** in the main text. We generated transition plots by calculating the mean rate and 95% confidence interval (in parentheses) for each transition rate, shown here when the threshold between low and high song durations is in the lowest third of observed values (red arrows), the middle third of observed values (yellow arrows), and the highest third of observed values (blue arrows). The bottom right panel shows the likelihood-ratio test *p*-value for each run of BayesTraits at each threshold value. These results for song duration follow a similar pattern to our results for syllable repertoire size (**Figure 5**). The combination of polygyny and very small song durations is unstable, with high rates of transition away from this state (red arrows). Similarly, the combination of polygyny with very large song durations is unstable (blue arrows).

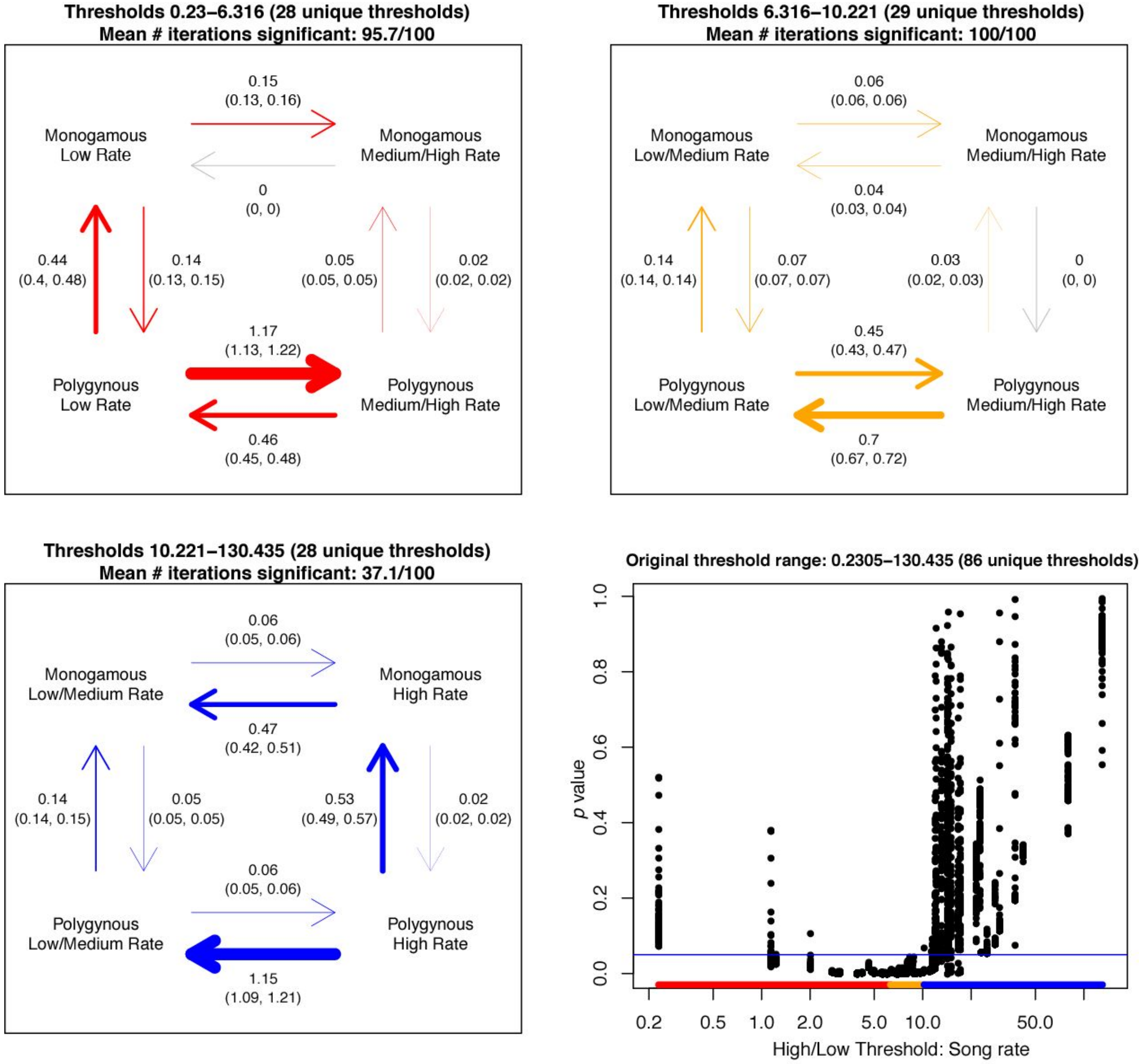

Supplementary Figure 22: Detecting correlated evolution of mating systems and song rate. We tested the correlated evolution of song rate and polygyny using BayesTraits, with song rate made binary based on a threshold delineating smaller versus larger song rates. Each observed value of song rate was used as the threshold for 100 runs of BayesTraits. Figure panels follow the same format as **Figures 5–6** in the main text. We generated transition plots by calculating the mean rate and 95% confidence interval (in parentheses) for each transition rate, shown here when the threshold between low and high song rates is in the lowest third of observed values (red arrows), the middle third of observed values (yellow arrows), and the highest third of observed values (blue arrows). The bottom right panel shows the likelihood-ratio test *p*-value for each run of BayesTraits at each threshold value. These results for song rate follow a similar pattern to our results for syllable repertoire size (**Figure 5**). The combination of polygyny and very low song rates is unstable, with high rates of transition away from this state (red arrows). Similarly, the combination of polygyny with very high song rates is unstable (blue arrows). In the middle range of threshold values (yellow arrows), polygyny significantly accelerates the rate of evolution of song rate.

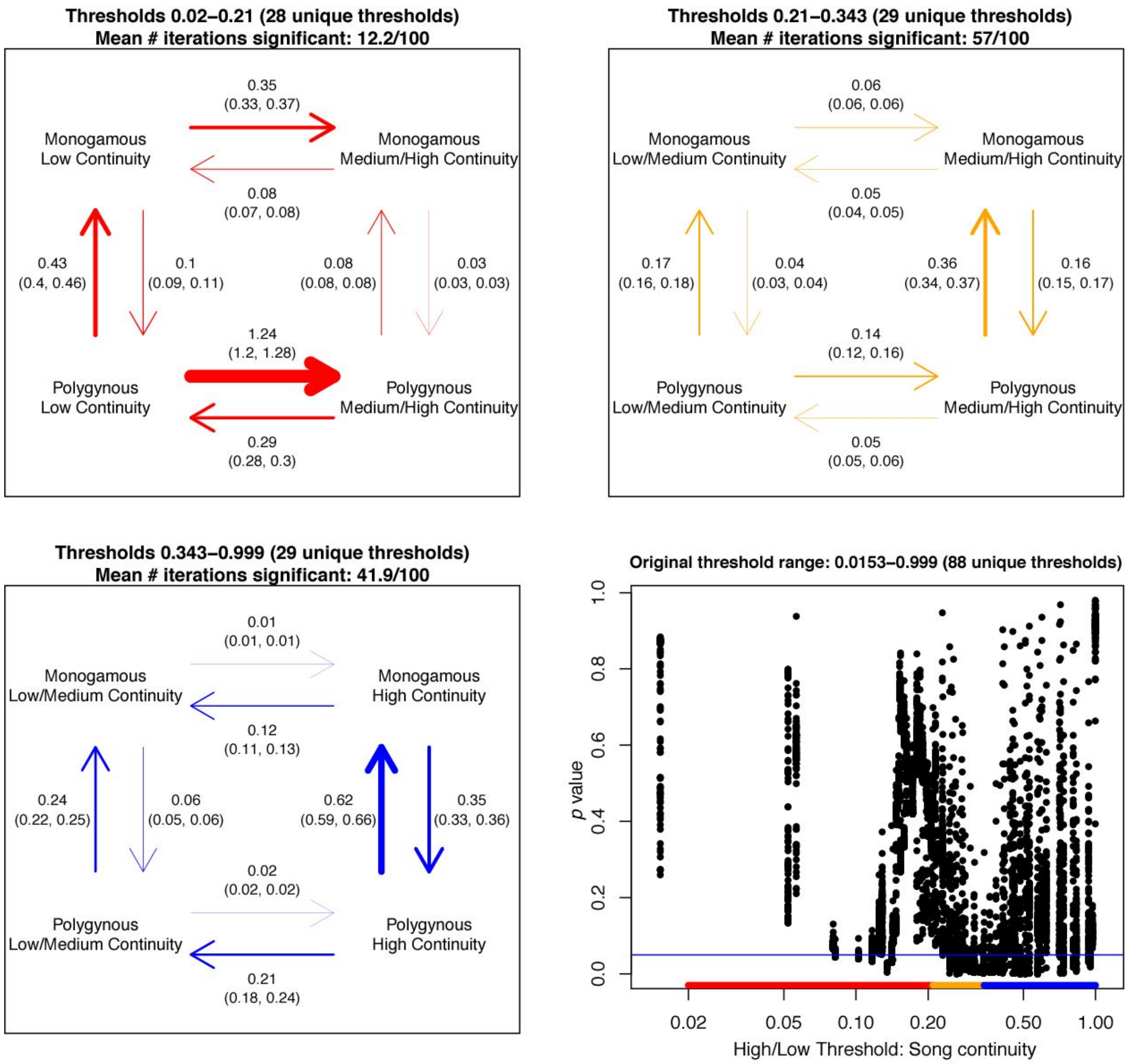

Supplementary Figure 23: Detecting correlated evolution of mating systems and song continuity. We tested the correlated evolution of song continuity and polygyny using BayesTraits, with song continuity made binary based on a threshold delineating smaller versus larger continuity values. Each observed value of song continuity was used as the threshold for 100 runs of BayesTraits. Figure panels follow the same format as **Figures 5–6** in the main text. We generated transition plots by calculating the mean rate and 95% confidence interval (in parentheses) for each transition rate, shown here when the threshold between low and high song continuities is in the lowest third of observed values (red arrows), the middle third of observed values (yellow arrows), and the highest third of observed values (blue arrows). The bottom right panel shows the likelihood-ratio test *p*-value for each run of BayesTraits at each threshold value. These results for song continuity follow a similar pattern to our results for syllable repertoire size (**Figure 5**). The combination of polygyny and very low values of song continuity is unstable, with high rates of transition away from this state (red arrows). Similarly, the combination of polygyny with very large values of song continuity is somewhat unstable (blue arrows).

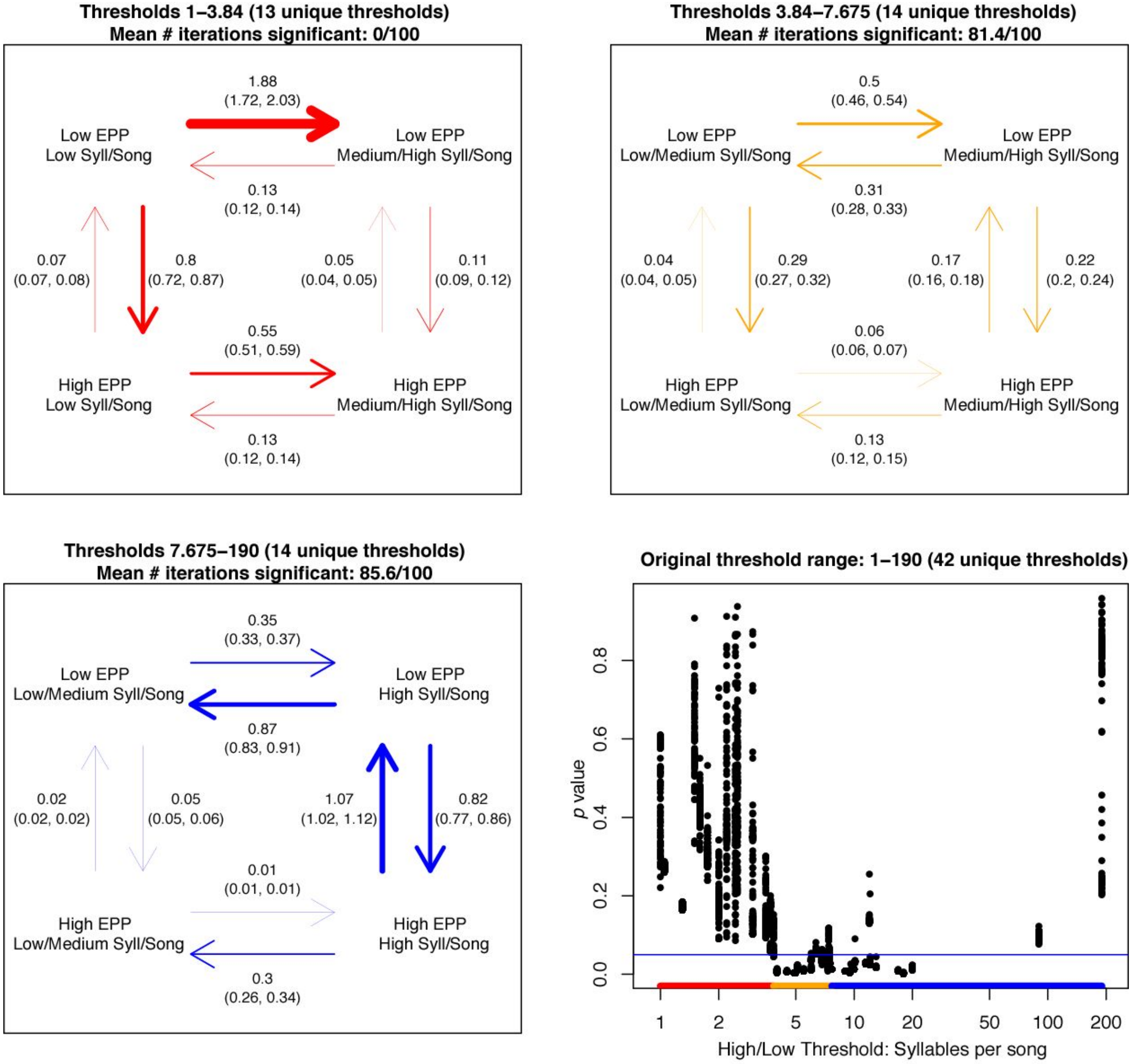

Supplementary Figure 24: Detecting correlated evolution of extra-pair paternity and syllables per song. We tested the correlated evolution of number of syllables per song and EPP using BayesTraits, with syllables per song made binary based on a threshold delineating smaller versus larger numbers of syllables per song. Each observed value of syllables per song was used as the threshold for 100 runs of BayesTraits. Figure panels follow the same format as **Figures 5–6** in the main text. We generated transition plots by calculating the mean rate and 95% confidence interval (in parentheses) for each transition rate, shown here when the threshold between low and high syllables per song is in the lowest third of observed values (red arrows), the middle third of observed values (yellow arrows), and the highest third of observed values (blue arrows). The bottom right panel shows the likelihood-ratio test *p*-value for each run of BayesTraits at each threshold value. These results for syllables per song are qualitatively similar to our results for syllable repertoire size (**Figure 6**). The combination of low EPP and very small numbers of syllables per song is unstable, with high rates of transition away from this state (red arrows), although these values are not significant. Similarly, the combination of high EPP with very large numbers of syllables per song is unstable, and there also appears to be elevated transition rates of EPP when number of syllables per song is high (blue arrows).

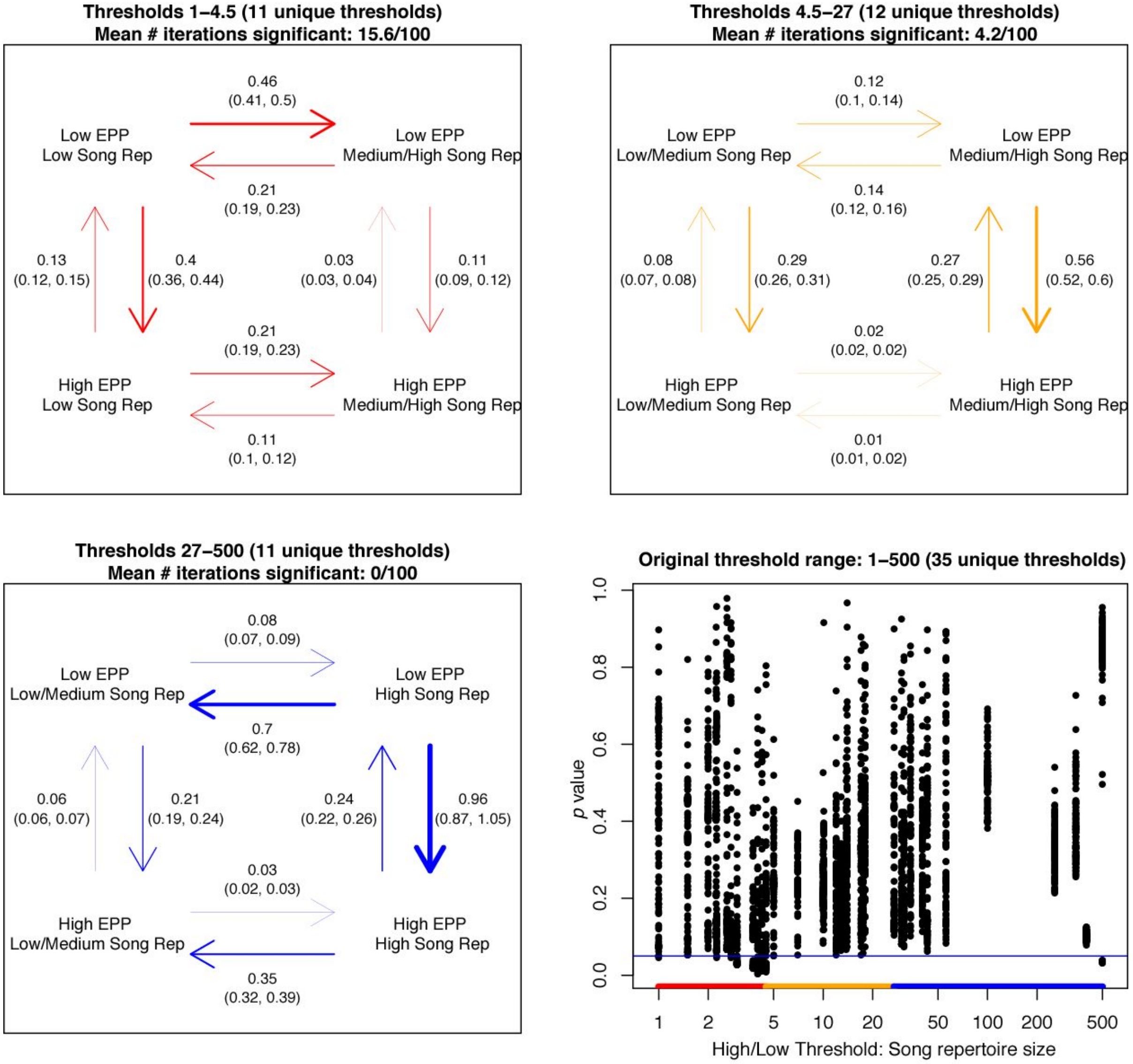

Supplementary Figure 25: Detecting correlated evolution of extra-pair paternity and song repertoire size. We tested the correlated evolution of song repertoire size and EPP using BayesTraits, with song repertoire size made binary based on a threshold delineating smaller versus larger song repertoires. Each observed value of song repertoire size was used as the threshold for 100 runs of BayesTraits. Figure panels follow the same format as **Figures 5–6** in the main text. We generated transition plots by calculating the mean rate and 95% confidence interval (in parentheses) for each transition rate, shown here when the threshold between low and high song repertoire sizes is in the lowest third of observed values (red arrows), the middle third of observed values (yellow arrows), and the highest third of observed values (blue arrows). The bottom right panel shows the likelihood-ratio test *p*-value for each run of BayesTraits at each threshold value. These results for song repertoire size show that very few runs of BayesTraits support a pattern of correlated evolution between song repertoire size and EPP.

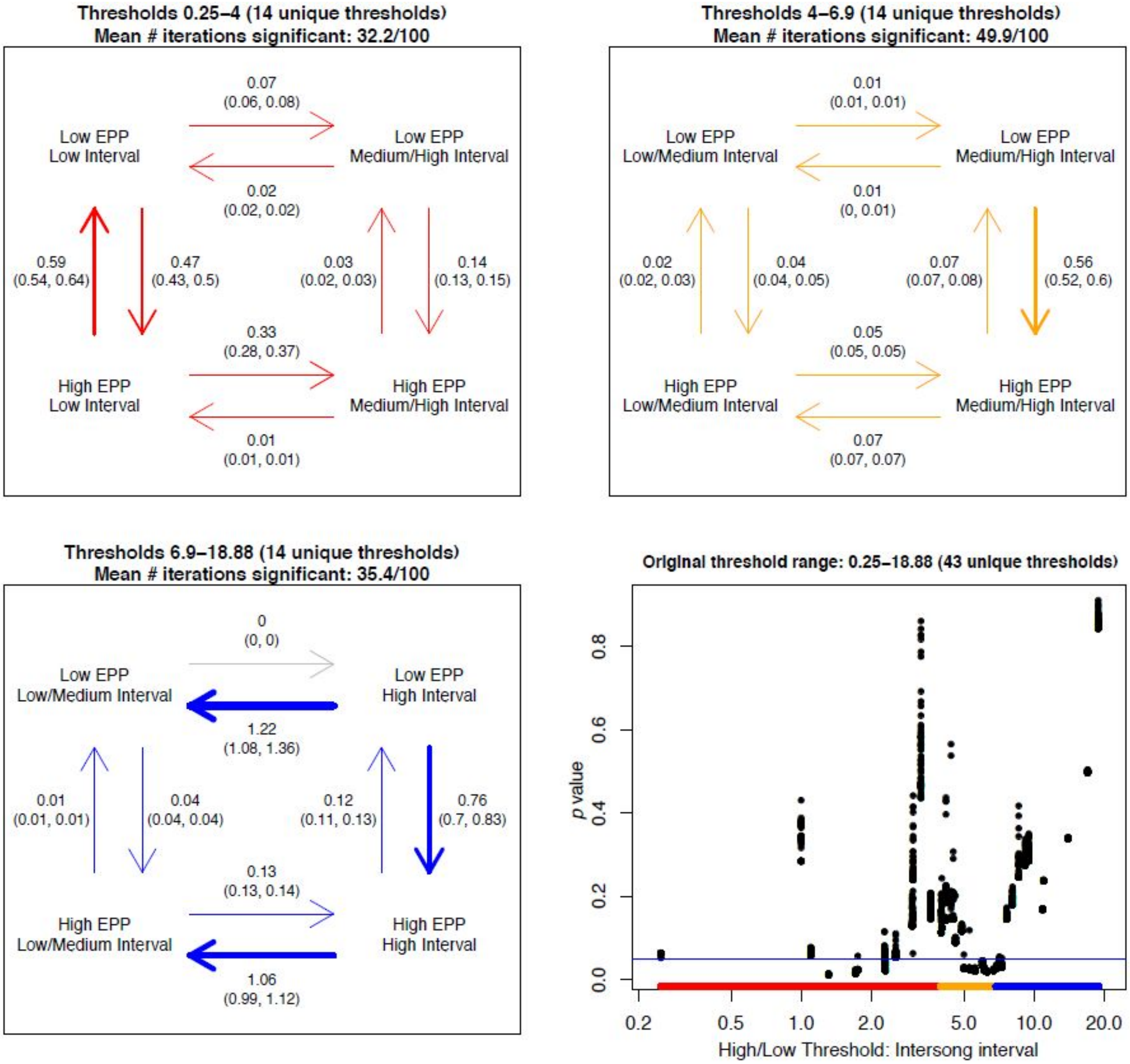

Supplementary Figure 26: Detecting correlated evolution of extra-pair paternity and intersong interval. We tested the correlated evolution of intersong interval and EPP using BayesTraits, with intersong interval made binary based on a threshold delineating smaller versus larger intersong intervals. Each observed value of intersong interval was used as the threshold for 100 runs of BayesTraits. Figure panels follow the same format as **Figures 5–6** in the main text. We generated transition plots by calculating the mean rate and 95% confidence interval (in parentheses) for each transition rate, shown here when the threshold between low and high intersong interval is in the lowest third of observed values (red arrows), the middle third of observed values (yellow arrows), and the highest third of observed values (blue arrows). The bottom right panel shows the likelihood-ratio test *p*-value for each run of BayesTraits at each threshold value. At low values of intersong interval, the transition between high and low EPP appears to be elevated (red arrows), and the highest values of intersong interval appear to quickly transition to lower values (blue arrows).

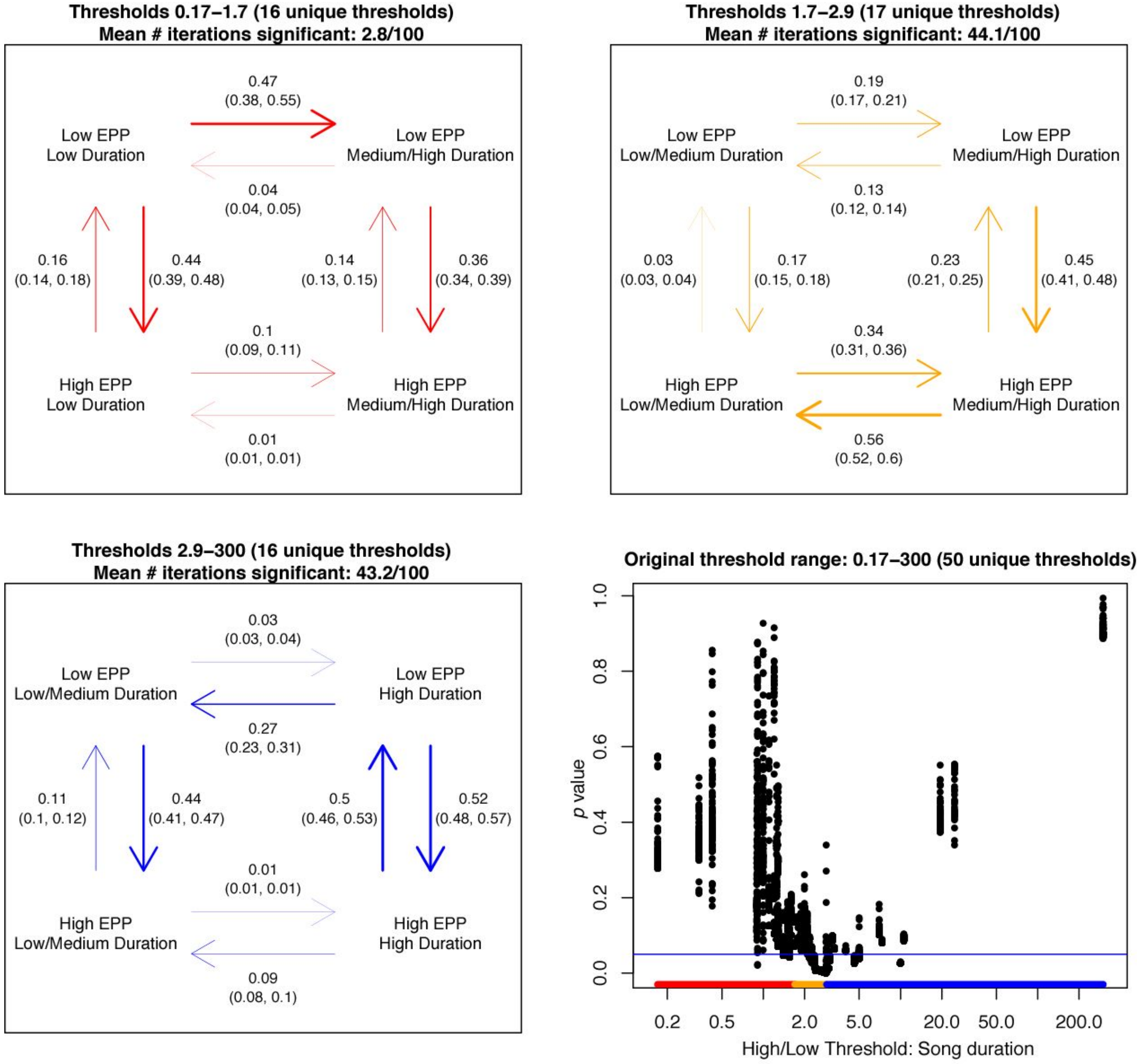

Supplementary Figure 27: Detecting correlated evolution of extra-pair paternity and song duration. We tested the correlated evolution of song duration and EPP using BayesTraits, with song duration made binary based on a threshold delineating smaller versus larger durations. Each observed value of song duration was used as the threshold for 100 runs of BayesTraits. Figure panels follow the same format as **Figures 5–6** in the main text. We generated transition plots by calculating the mean rate and 95% confidence interval (in parentheses) for each transition rate, shown here when the threshold between low and high song durations is in the lowest third of observed values (red arrows), the middle third of observed values (yellow arrows), and the highest third of observed values (blue arrows). The bottom right panel shows the likelihood-ratio test *p*-value for each run of BayesTraits at each threshold value. These results for song duration suggest that high values of song duration are correlated with elevated rates of evolution of EPP (yellow arrows, blue arrows). In addition, high EPP appears to elevate the rate of transition in song duration for the middle range of threshold values (yellow arrows).

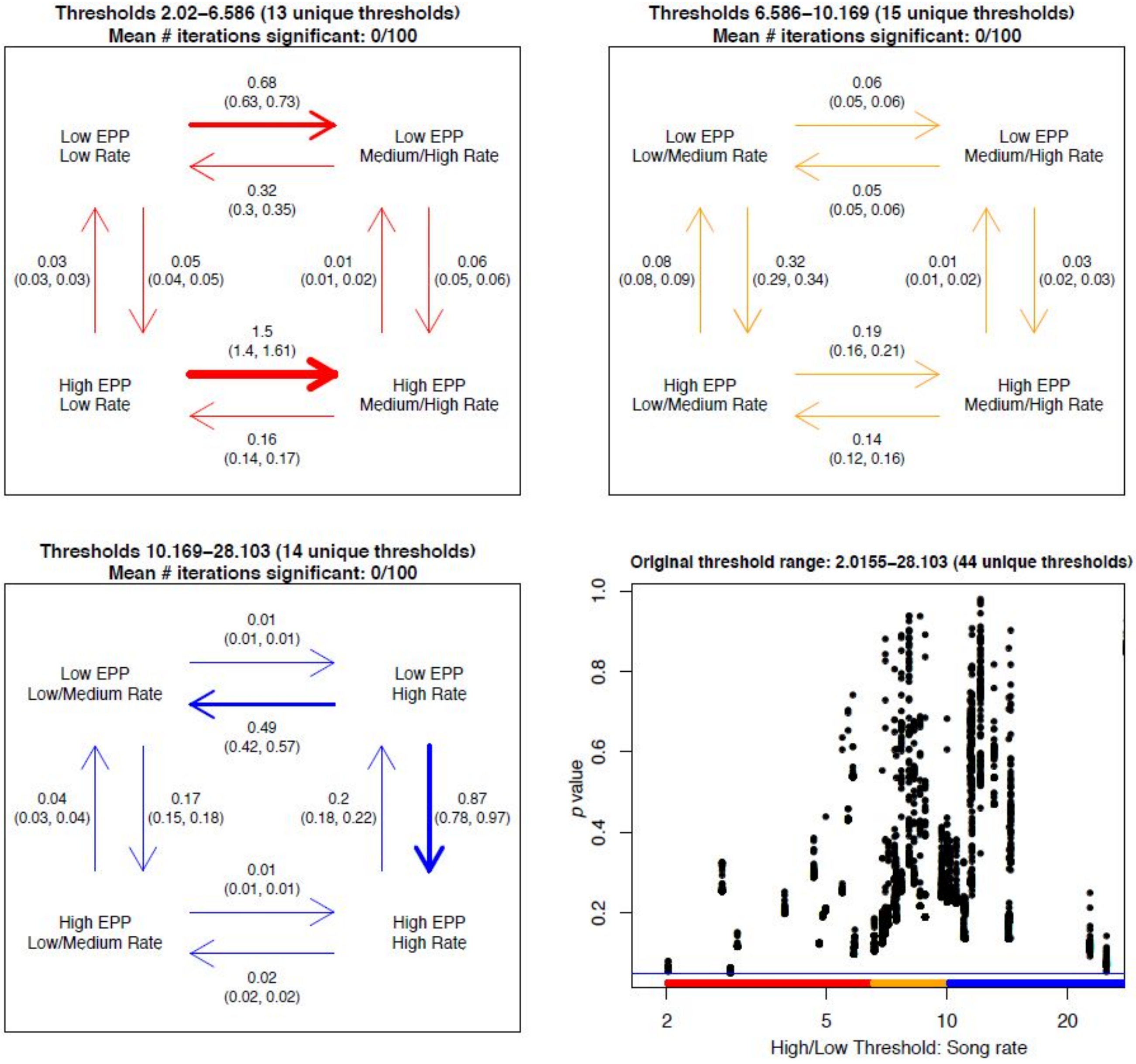

Supplementary Figure 28: Detecting correlated evolution of extra-pair paternity and song rate. We tested the correlated evolution of song rate and EPP using BayesTraits, with song rate made binary based on a threshold delineating smaller versus larger song rates. Each observed value of song rate was used as the threshold for 100 runs of BayesTraits. Figure panels follow the same format as **Figures 5–6** in the main text. We generated transition plots by calculating the mean rate and 95% confidence interval (in parentheses) for each transition rate, shown here when the threshold between low and high song rate is in the lowest third of observed values (red arrows), the middle third of observed values (yellow arrows), and the highest third of observed values (blue arrows). The bottom right panel shows the likelihood-ratio test *p*-value for each run of BayesTraits at each threshold value. These results for song rate show that no runs of BayesTraits support a pattern of correlated evolution between song rate and EPP.

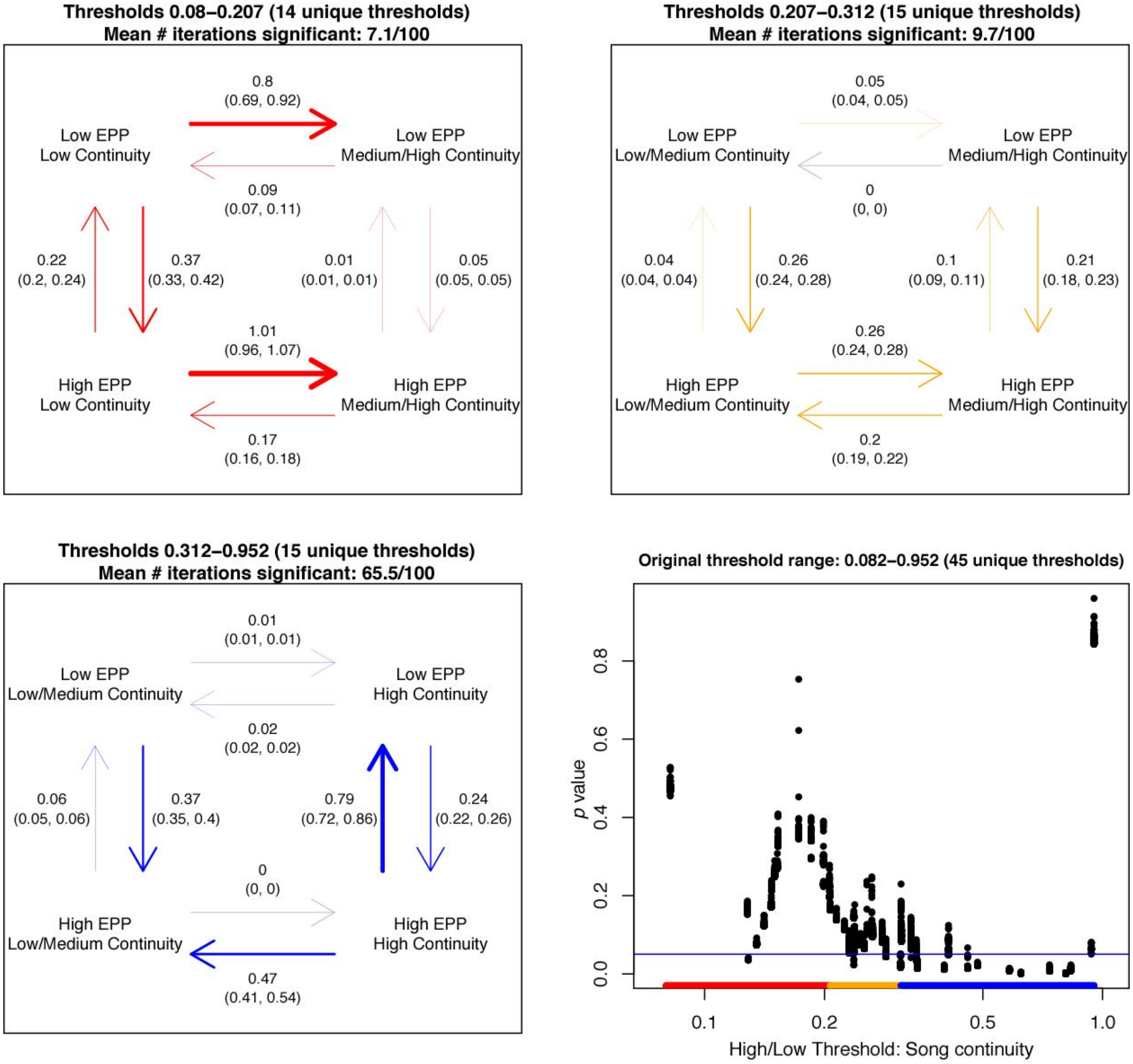

Supplementary Figure 29: Detecting correlated evolution of extra-pair paternity and song continuity. We tested the correlated evolution of song continuity and EPP using BayesTraits, with song continuity made binary based on a threshold delineating smaller versus larger song continuity values. Each observed value of song continuity was used as the threshold for 100 runs of BayesTraits. Figure panels follow the same format as **Figures 5–6** in the main text. We generated transition plots by calculating the mean rate and 95% confidence interval (in parentheses) for each transition rate, shown here when the threshold between low and high song continuity is in the lowest third of observed values (red arrows), the middle third of observed values (yellow arrows), and the highest third of observed values (blue arrows). The bottom right panel shows the likelihood-ratio test *p*-value for each run of BayesTraits at each threshold value. These results for song continuity suggest that low values of song continuity might be unstable, with elevated rates of transition away from low song continuity (red arrows). In addition, the combination of high EPP with high values of song continuity is unstable, with elevated rates of transition leaving this state and most iterations significant (blue arrows).

Supplementary Figure 30 (following pages): Jackknife analysis of correlated evolution between mating system and syllable repertoire size with Bayestraits. We removed one family at a time and repeated our BayesTraits analysis to detect correlated evolution between syllable repertoire size and mating system. We found consistent results with the analysis of all families, shown in Fig. 5.

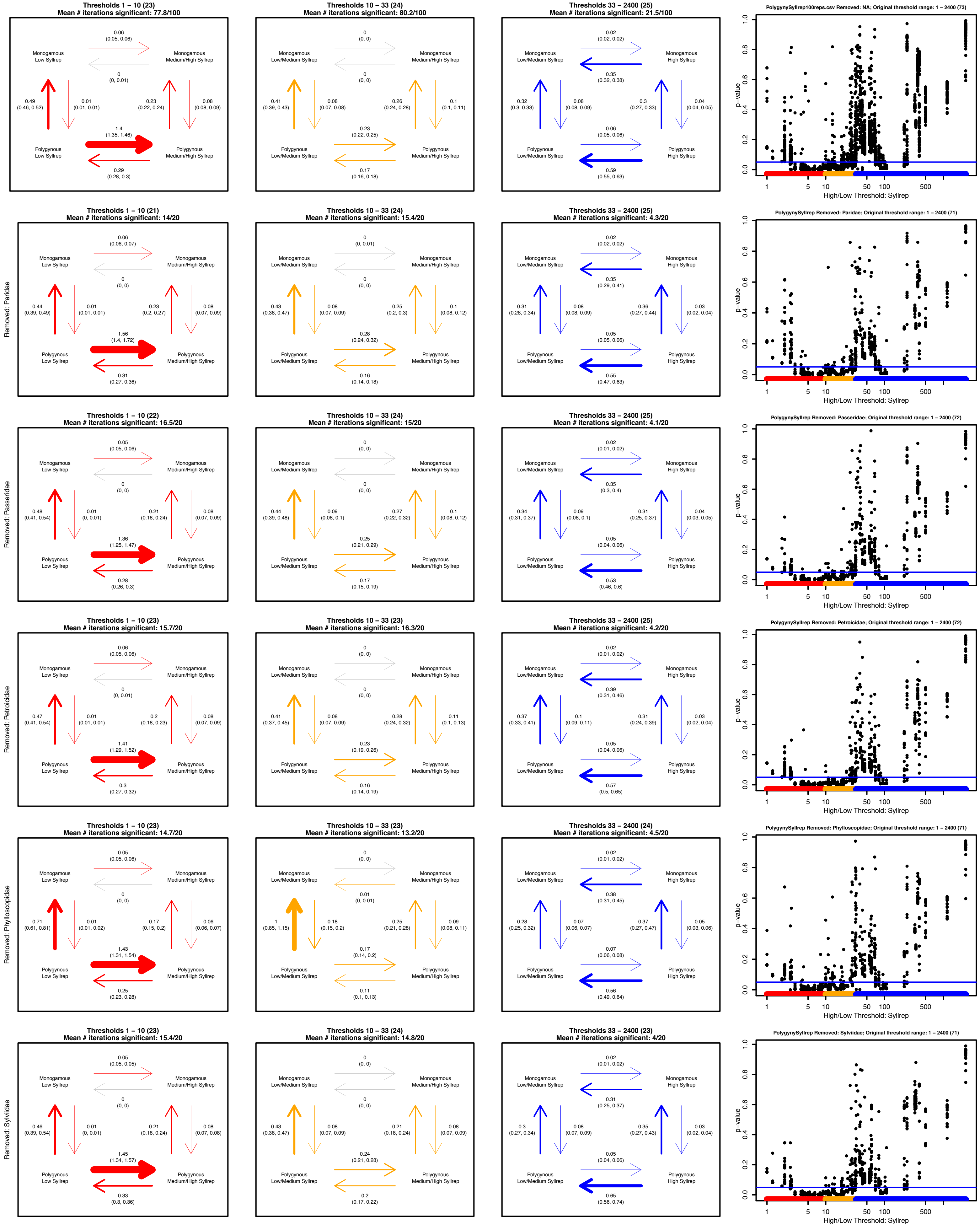
Thresholds 1 – 10 (23)
Mean # iterations significant: 77.8/100
0.06 (0.05, 0.06)
Monogamous Low Syllrep
Monogamous Medium/High Syllrep
0 (0, 0.01)
0.49 (0.46, 0.52)
0.01 (0.01, 0.01)
0.23 (0.22, 0.24)
0.08 (0.08, 0.09)
1.4 (1.35, 1.46)
Polygynous Low Syllrep
Polygynous Medium/High Syllrep
0.29 (0.28, 0.3)
Thresholds 10 – 33 (24)
Mean # iterations significant: 80.2/100
0 (0, 0)
Monogamous Low/Medium Syllrep
Monogamous Medium/High Syllrep
0 (0, 0)
0.41 (0.39, 0.43)
0.08 (0.07, 0.08)
0.26 (0.24, 0.28)
0.1 (0.1, 0.11)
0.23 (0.22, 0.25)
Polygynous Low/Medium Syllrep
Polygynous Medium/High Syllrep
0.17 (0.16, 0.18)
Thresholds 33 – 2400 (25)
Mean # iterations significant: 21.5/100
0.02 (0.02, 0.02)
Monogamous Low/Medium Syllrep
Monogamous High Syllrep
0.35 (0.32, 0.38)
0.32 (0.3, 0.33)
0.08 (0.08, 0.09)
0.3 (0.27, 0.33)
0.04 (0.04, 0.05)
0.06 (0.05, 0.06)
Polygynous Low/Medium Syllrep
Polygynous High Syllrep
0.59 (0.55, 0.63)
PolygynySyllrep100reps.csv Removed: NA; Original threshold range: 1 – 2400 (73)
p–value
High/Low Threshold: Syllrep
Removed: Paridae
Thresholds 1 – 10 (21)
Mean # iterations significant: 14/20
0.06 (0.06, 0.07)
0 (0, 0)
0.44 (0.39, 0.49)
0.01 (0.01, 0.01)
0.23 (0.2, 0.27)
0.08 (0.07, 0.09)
1.56 (1.4, 1.72)
0.31 (0.27, 0.36)
Thresholds 10 – 33 (24)
Mean # iterations significant: 15.4/20
0 (0, 0.01)
0 (0, 0)
0.43 (0.38, 0.47)
0.08 (0.07, 0.09)
0.25 (0.2, 0.3)
0.1 (0.08, 0.12)
0.28 (0.24, 0.32)
0.16 (0.14, 0.18)
Thresholds 33 – 2400 (25)
Mean # iterations significant: 4.3/20
0.02 (0.02, 0.02)
0.35 (0.29, 0.41)
0.31 (0.28, 0.34)
0.08 (0.08, 0.09)
0.36 (0.27, 0.44)
0.03 (0.02, 0.04)
0.05 (0.05, 0.06)
0.55 (0.47, 0.63)
PolygynySyllrep Removed: Paridae; Original threshold range: 1 – 2400 (71)
Removed: Passeridae
Thresholds 1 – 10 (22)
Mean # iterations significant: 16.5/20
0.05 (0.05, 0.06)
0 (0, 0)
0.48 (0.41, 0.54)
0.01 (0, 0.01)
0.21 (0.18, 0.24)
0.08 (0.07, 0.09)
1.36 (1.25, 1.47)
0.28 (0.26, 0.3)
Thresholds 10 – 33 (24)
Mean # iterations significant: 15/20
0 (0, 0)
0 (0, 0)
0.44 (0.39, 0.48)
0.09 (0.08, 0.1)
0.27 (0.22, 0.32)
0.1 (0.08, 0.12)
0.25 (0.21, 0.29)
0.17 (0.15, 0.19)
Thresholds 33 – 2400 (25)
Mean # iterations significant: 4.1/20
0.02 (0.01, 0.02)
0.35 (0.3, 0.4)
0.34 (0.31, 0.37)
0.09 (0.08, 0.1)
0.31 (0.25, 0.37)
0.04 (0.03, 0.05)
0.05 (0.04, 0.06)
0.53 (0.46, 0.6)
PolygynySyllrep Removed: Passeridae; Original threshold range: 1 – 2400 (72)
Removed: Petroicidae
Thresholds 1 – 10 (23)
Mean # iterations significant: 15.7/20
0.06 (0.05, 0.06)
0 (0, 0.01)
0.47 (0.41, 0.54)
0.01 (0.01, 0.01)
0.2 (0.18, 0.23)
0.08 (0.07, 0.09)
1.41 (1.29, 1.52)
0.3 (0.27, 0.32)
Thresholds 10 – 33 (23)
Mean # iterations significant: 16.3/20
0 (0, 0)
0 (0, 0)
0.41 (0.37, 0.45)
0.08 (0.07, 0.09)
0.28 (0.24, 0.32)
0.11 (0.1, 0.13)
0.23 (0.19, 0.26)
0.16 (0.14, 0.19)
Thresholds 33 – 2400 (25)
Mean # iterations significant: 4.2/20
0.02 (0.01, 0.02)
0.39 (0.31, 0.46)
0.37 (0.33, 0.41)
0.1 (0.09, 0.11)
0.31 (0.24, 0.39)
0.03 (0.02, 0.04)
0.05 (0.04, 0.06)
0.57 (0.5, 0.65)
PolygynySyllrep Removed: Petroicidae; Original threshold range: 1 – 2400 (72)
Removed: Phylloscopidae
Thresholds 1 – 10 (23)
Mean # iterations significant: 14.7/20
0.05 (0.05, 0.06)
0 (0, 0)
0.71 (0.61, 0.81)
0.01 (0.01, 0.02)
0.17 (0.15, 0.2)
0.06 (0.06, 0.07)
1.43 (1.31, 1.54)
0.25 (0.23, 0.28)
Thresholds 10 – 33 (23)
Mean # iterations significant: 13.2/20
0 (0, 0)
0.01 (0, 0.01)
1 (0.85, 1.15)
0.18 (0.15, 0.2)
0.25 (0.21, 0.28)
0.09 (0.08, 0.11)
0.17 (0.14, 0.2)
0.11 (0.1, 0.13)
Thresholds 33 – 2400 (24)
Mean # iterations significant: 4.5/20
0.02 (0.01, 0.02)
0.38 (0.31, 0.45)
0.28 (0.25, 0.32)
0.07 (0.06, 0.07)
0.37 (0.27, 0.47)
0.05 (0.03, 0.06)
0.07 (0.06, 0.08)
0.56 (0.49, 0.64)
PolygynySyllrep Removed: Phylloscopidae; Original threshold range: 1 – 2400 (71)
Removed: Sylviidae
Thresholds 1 – 10 (23)
Mean # iterations significant: 15.4/20
0.05 (0.05, 0.05)
0 (0, 0)
0.46 (0.39, 0.54)
0.01 (0, 0.01)
0.21 (0.18, 0.24)
0.08 (0.07, 0.08)
1.45 (1.34, 1.57)
0.33 (0.3, 0.36)
Thresholds 10 – 33 (24)
Mean # iterations significant: 14.8/20
0 (0, 0)
0 (0, 0)
0.43 (0.38, 0.47)
0.08 (0.07, 0.09)
0.21 (0.18, 0.24)
0.08 (0.07, 0.09)
0.24 (0.21, 0.28)
0.2 (0.17, 0.22)
Thresholds 33 – 2400 (23)
Mean # iterations significant: 4/20
0.02 (0.01, 0.02)
0.31 (0.25, 0.37)
0.3 (0.27, 0.34)
0.08 (0.07, 0.09)
0.35 (0.27, 0.43)
0.03 (0.02, 0.04)
0.05 (0.04, 0.06)
0.65 (0.56, 0.74)
PolygynySyllrep Removed: Sylviidae; Original threshold range: 1 – 2400 (71)

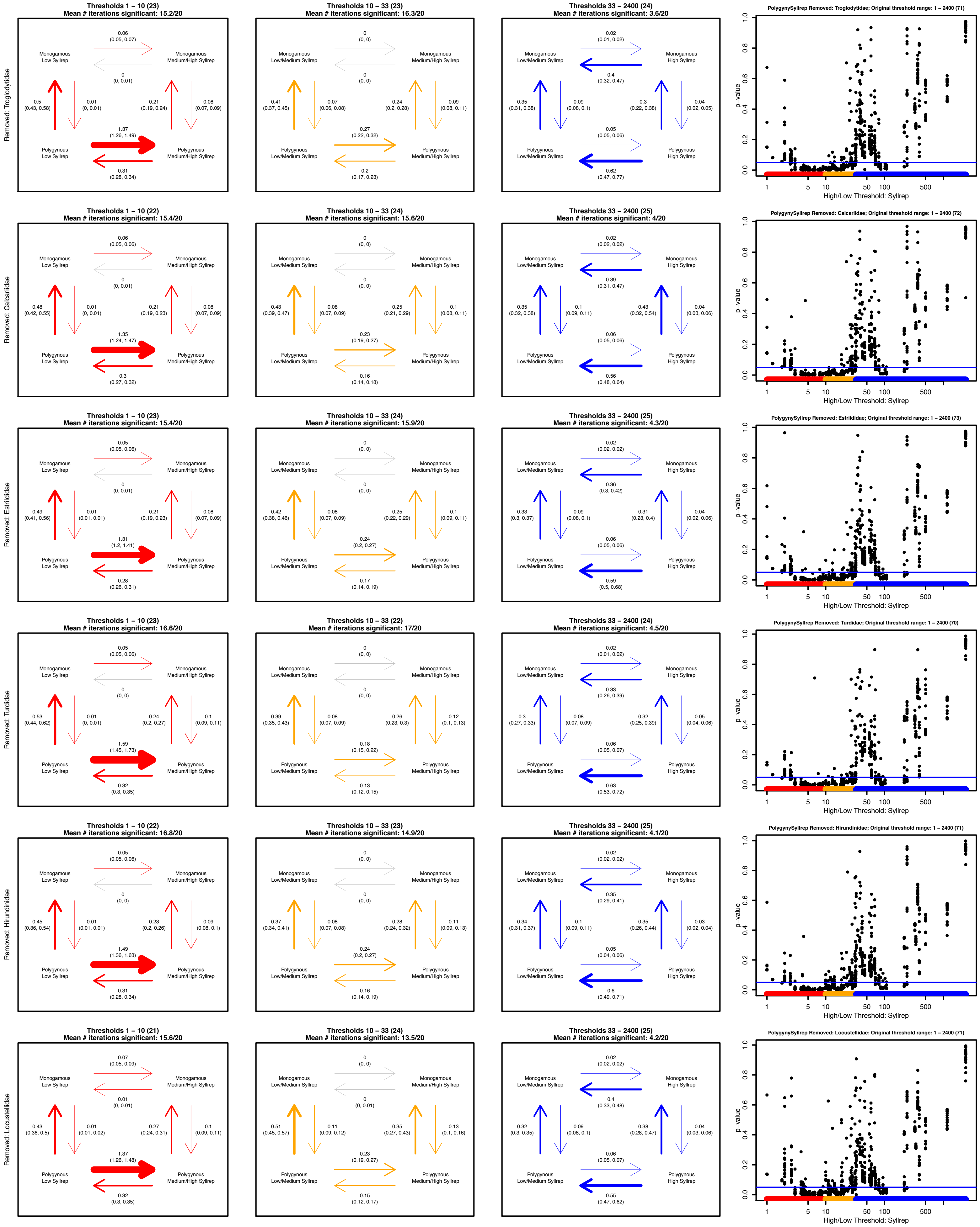

Removed: Troglodytidae
Thresholds 1 – 10 (23)
Mean # iterations significant: 15.2/20
Monogamous Low Syllrep
Monogamous Medium/High Syllrep
Polygynous Low Syllrep
Polygynous Medium/High Syllrep
0.06 (0.05, 0.07)
0 (0, 0.01)
0.5 (0.43, 0.58)
0.01 (0, 0.01)
0.21 (0.19, 0.24)
0.08 (0.07, 0.09)
1.37 (1.26, 1.49)
0.31 (0.28, 0.34)
Thresholds 10 – 33 (23)
Mean # iterations significant: 16.3/20
Monogamous Low/Medium Syllrep
Monogamous Medium/High Syllrep
Polygynous Low/Medium Syllrep
Polygynous Medium/High Syllrep
0 (0, 0)
0 (0, 0)
0.41 (0.37, 0.45)
0.07 (0.06, 0.08)
0.24 (0.2, 0.28)
0.09 (0.08, 0.11)
0.27 (0.22, 0.32)
0.2 (0.17, 0.23)
Thresholds 33 – 2400 (24)
Mean # iterations significant: 3.6/20
Monogamous Low/Medium Syllrep
Monogamous High Syllrep
Polygynous Low/Medium Syllrep
Polygynous High Syllrep
0.02 (0.01, 0.02)
0.4 (0.32, 0.47)
0.35 (0.31, 0.38)
0.09 (0.08, 0.1)
0.3 (0.22, 0.38)
0.04 (0.02, 0.05)
0.05 (0.05, 0.06)
0.62 (0.47, 0.77)
PolygynySyllrep Removed: Troglodytidae; Original threshold range: 1 – 2400 (71)
p−value
0.0 0.2 0.4 0.6 0.8 1.0
1 5 10 50 100 500
High/Low Threshold: Syllrep
Removed: Calcariidae
Thresholds 1 – 10 (22)
Mean # iterations significant: 15.4/20
Monogamous Low Syllrep
Monogamous Medium/High Syllrep
Polygynous Low Syllrep
Polygynous Medium/High Syllrep
0.06 (0.05, 0.06)
0 (0, 0.01)
0.48 (0.42, 0.55)
0.01 (0, 0.01)
0.21 (0.19, 0.23)
0.08 (0.07, 0.09)
1.35 (1.24, 1.47)
0.3 (0.27, 0.32)
Thresholds 10 – 33 (24)
Mean # iterations significant: 15.6/20
Monogamous Low/Medium Syllrep
Monogamous Medium/High Syllrep
Polygynous Low/Medium Syllrep
Polygynous Medium/High Syllrep
0 (0, 0)
0 (0, 0)
0.43 (0.39, 0.47)
0.08 (0.07, 0.09)
0.25 (0.21, 0.29)
0.1 (0.08, 0.11)
0.23 (0.19, 0.27)
0.16 (0.14, 0.18)
Thresholds 33 – 2400 (25)
Mean # iterations significant: 4/20
Monogamous Low/Medium Syllrep
Monogamous High Syllrep
Polygynous Low/Medium Syllrep
Polygynous High Syllrep
0.02 (0.02, 0.02)
0.39 (0.31, 0.47)
0.35 (0.32, 0.38)
0.1 (0.09, 0.11)
0.43 (0.32, 0.54)
0.04 (0.03, 0.06)
0.06 (0.05, 0.06)
0.56 (0.48, 0.64)
PolygynySyllrep Removed: Calcariidae; Original threshold range: 1 – 2400 (72)
p−value
0.0 0.2 0.4 0.6 0.8 1.0
1 5 10 50 100 500
High/Low Threshold: Syllrep
Removed: Estrildidae
Thresholds 1 – 10 (23)
Mean # iterations significant: 15.4/20
Monogamous Low Syllrep
Monogamous Medium/High Syllrep
Polygynous Low Syllrep
Polygynous Medium/High Syllrep
0.05 (0.05, 0.06)
0 (0, 0.01)
0.49 (0.41, 0.56)
0.01 (0.01, 0.01)
0.21 (0.19, 0.23)
0.08 (0.07, 0.09)
1.31 (1.2, 1.41)
0.28 (0.26, 0.31)
Thresholds 10 – 33 (24)
Mean # iterations significant: 15.9/20
Monogamous Low/Medium Syllrep
Monogamous Medium/High Syllrep
Polygynous Low/Medium Syllrep
Polygynous Medium/High Syllrep
0 (0, 0)
0 (0, 0)
0.42 (0.38, 0.46)
0.08 (0.07, 0.09)
0.25 (0.22, 0.29)
0.1 (0.09, 0.11)
0.24 (0.2, 0.27)
0.17 (0.14, 0.19)
Thresholds 33 – 2400 (25)
Mean # iterations significant: 4.3/20
Monogamous Low/Medium Syllrep
Monogamous High Syllrep
Polygynous Low/Medium Syllrep
Polygynous High Syllrep
0.02 (0.02, 0.02)
0.36 (0.3, 0.42)
0.33 (0.3, 0.37)
0.09 (0.08, 0.1)
0.31 (0.23, 0.4)
0.04 (0.02, 0.06)
0.06 (0.05, 0.06)
0.59 (0.5, 0.68)
PolygynySyllrep Removed: Estrildidae; Original threshold range: 1 – 2400 (73)
p−value
0.0 0.2 0.4 0.6 0.8 1.0
1 5 10 50 100 500
High/Low Threshold: Syllrep
Removed: Turdidae
Thresholds 1 – 10 (23)
Mean # iterations significant: 16.6/20
Monogamous Low Syllrep
Monogamous Medium/High Syllrep
Polygynous Low Syllrep
Polygynous Medium/High Syllrep
0.05 (0.05, 0.06)
0 (0, 0)
0.53 (0.44, 0.62)
0.01 (0, 0.01)
0.24 (0.2, 0.27)
0.1 (0.09, 0.11)
1.59 (1.45, 1.73)
0.32 (0.3, 0.35)
Thresholds 10 – 33 (22)
Mean # iterations significant: 17/20
Monogamous Low/Medium Syllrep
Monogamous Medium/High Syllrep
Polygynous Low/Medium Syllrep
Polygynous Medium/High Syllrep
0 (0, 0)
0 (0, 0)
0.39 (0.35, 0.43)
0.08 (0.07, 0.09)
0.26 (0.23, 0.3)
0.12 (0.1, 0.13)
0.18 (0.15, 0.22)
0.13 (0.12, 0.15)
Thresholds 33 – 2400 (24)
Mean # iterations significant: 4.5/20
Monogamous Low/Medium Syllrep
Monogamous High Syllrep
Polygynous Low/Medium Syllrep
Polygynous High Syllrep
0.02 (0.01, 0.02)
0.33 (0.26, 0.39)
0.3 (0.27, 0.33)
0.08 (0.07, 0.09)
0.32 (0.25, 0.39)
0.05 (0.04, 0.06)
0.06 (0.05, 0.07)
0.63 (0.53, 0.72)
PolygynySyllrep Removed: Turdidae; Original threshold range: 1 – 2400 (70)
p−value
0.0 0.2 0.4 0.6 0.8 1.0
1 5 10 50 100 500
High/Low Threshold: Syllrep
Removed: Hirundinidae
Thresholds 1 – 10 (22)
Mean # iterations significant: 16.8/20
Monogamous Low Syllrep
Monogamous Medium/High Syllrep
Polygynous Low Syllrep
Polygynous Medium/High Syllrep
0.05 (0.05, 0.06)
0 (0, 0)
0.45 (0.36, 0.54)
0.01 (0.01, 0.01)
0.23 (0.2, 0.26)
0.09 (0.08, 0.1)
1.49 (1.36, 1.63)
0.31 (0.28, 0.34)
Thresholds 10 – 33 (23)
Mean # iterations significant: 14.9/20
Monogamous Low/Medium Syllrep
Monogamous Medium/High Syllrep
Polygynous Low/Medium Syllrep
Polygynous Medium/High Syllrep
0 (0, 0)
0 (0, 0)
0.37 (0.34, 0.41)
0.08 (0.07, 0.08)
0.28 (0.24, 0.32)
0.11 (0.09, 0.13)
0.24 (0.2, 0.27)
0.16 (0.14, 0.19)
Thresholds 33 – 2400 (25)
Mean # iterations significant: 4.1/20
Monogamous Low/Medium Syllrep
Monogamous High Syllrep
Polygynous Low/Medium Syllrep
Polygynous High Syllrep
0.02 (0.02, 0.02)
0.35 (0.29, 0.41)
0.34 (0.31, 0.37)
0.1 (0.09, 0.11)
0.35 (0.26, 0.44)
0.03 (0.02, 0.04)
0.05 (0.04, 0.06)
0.6 (0.49, 0.71)
PolygynySyllrep Removed: Hirundinidae; Original threshold range: 1 – 2400 (71)
p−value
0.0 0.2 0.4 0.6 0.8 1.0
1 5 10 50 100 500
High/Low Threshold: Syllrep
Removed: Locustellidae
Thresholds 1 – 10 (21)
Mean # iterations significant: 15.6/20
Monogamous Low Syllrep
Monogamous Medium/High Syllrep
Polygynous Low Syllrep
Polygynous Medium/High Syllrep
0.07 (0.05, 0.09)
0.01 (0, 0.01)
0.43 (0.36, 0.5)
0.01 (0.01, 0.02)
0.27 (0.24, 0.31)
0.1 (0.09, 0.11)
1.37 (1.26, 1.48)
0.32 (0.3, 0.35)
Thresholds 10 – 33 (24)
Mean # iterations significant: 13.5/20
Monogamous Low/Medium Syllrep
Monogamous Medium/High Syllrep
Polygynous Low/Medium Syllrep
Polygynous Medium/High Syllrep
0 (0, 0)
0 (0, 0.01)
0.51 (0.45, 0.57)
0.11 (0.09, 0.12)
0.35 (0.27, 0.43)
0.13 (0.1, 0.16)
0.23 (0.19, 0.27)
0.15 (0.12, 0.17)
Thresholds 33 – 2400 (25)
Mean # iterations significant: 4.2/20
Monogamous Low/Medium Syllrep
Monogamous High Syllrep
Polygynous Low/Medium Syllrep
Polygynous High Syllrep
0.02 (0.02, 0.02)
0.4 (0.33, 0.48)
0.32 (0.3, 0.35)
0.09 (0.08, 0.1)
0.38 (0.28, 0.47)
0.04 (0.03, 0.06)
0.06 (0.05, 0.07)
0.55 (0.47, 0.62)
PolygynySyllrep Removed: Locustellidae; Original threshold range: 1 – 2400 (71)
p−value
0.0 0.2 0.4 0.6 0.8 1.0
1 5 10 50 100 500
High/Low Threshold: Syllrep

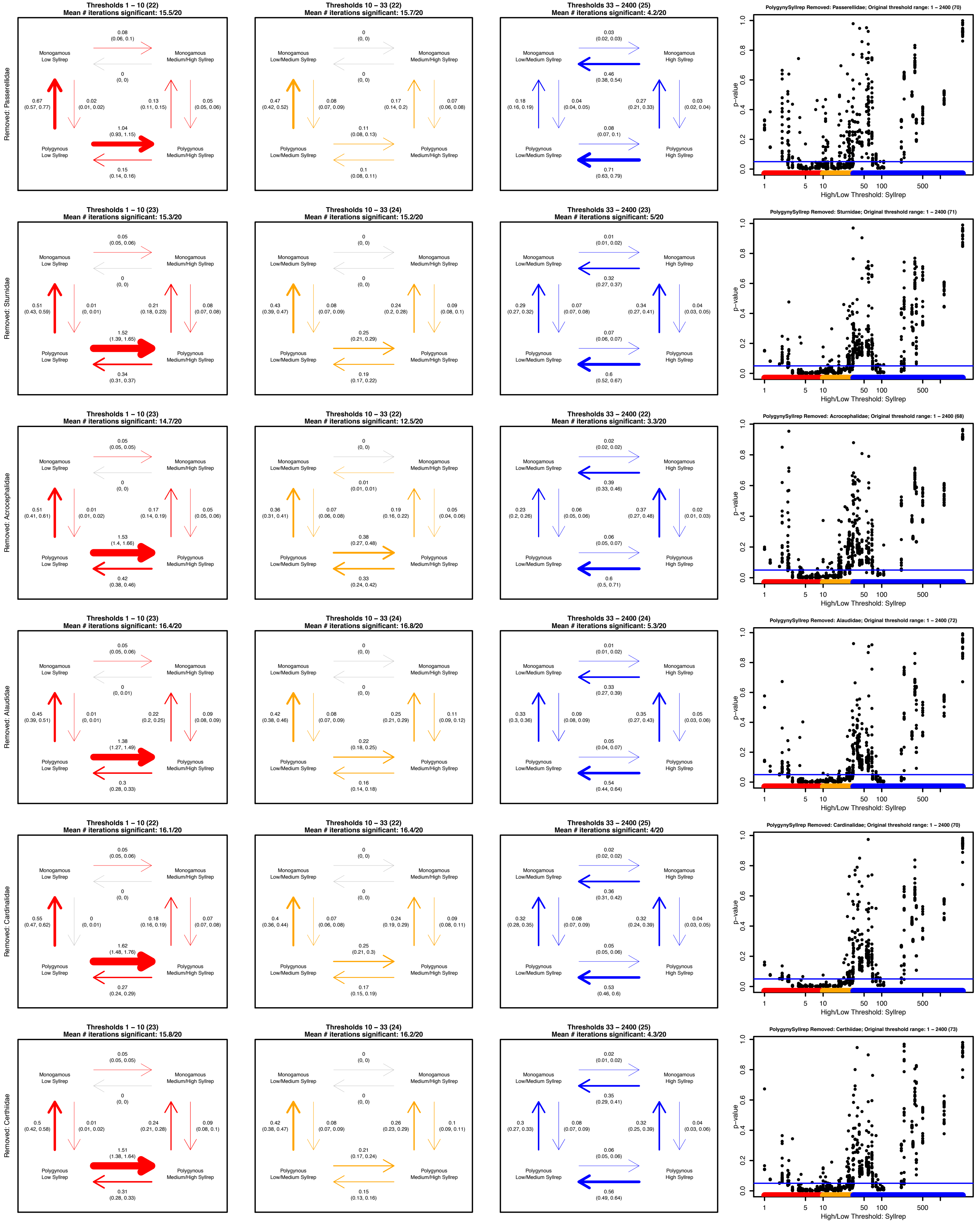

Removed: Passerellidae
Thresholds 1 – 10 (22)
Mean # iterations significant: 15.5/20
0.08 (0.06, 0.1)
Monogamous Low Syllrep
Monogamous Medium/High Syllrep
0 (0, 0)
0.67 (0.57, 0.77)
0.02 (0.01, 0.02)
0.13 (0.11, 0.15)
0.05 (0.05, 0.06)
1.04 (0.93, 1.15)
Polygynous Low Syllrep
Polygynous Medium/High Syllrep
0.15 (0.14, 0.16)
Thresholds 10 – 33 (22)
Mean # iterations significant: 15.7/20
0 (0, 0)
Monogamous Low/Medium Syllrep
Monogamous Medium/High Syllrep
0 (0, 0)
0.47 (0.42, 0.52)
0.08 (0.07, 0.09)
0.17 (0.14, 0.2)
0.07 (0.06, 0.08)
0.11 (0.08, 0.13)
Polygynous Low/Medium Syllrep
Polygynous Medium/High Syllrep
0.1 (0.08, 0.11)
Thresholds 33 – 2400 (25)
Mean # iterations significant: 4.2/20
0.03 (0.02, 0.03)
Monogamous Low/Medium Syllrep
Monogamous High Syllrep
0.46 (0.38, 0.54)
0.18 (0.16, 0.19)
0.04 (0.04, 0.05)
0.27 (0.21, 0.33)
0.03 (0.02, 0.04)
0.08 (0.07, 0.1)
Polygynous Low/Medium Syllrep
Polygynous High Syllrep
0.71 (0.63, 0.79)
PolygynySyllrep Removed: Passerellidae; Original threshold range: 1 – 2400 (70)
p−value
0.0 0.2 0.4 0.6 0.8 1.0
1 5 10 50 100 500
High/Low Threshold: Syllrep
Removed: Sturnidae
Thresholds 1 – 10 (23)
Mean # iterations significant: 15.3/20
0.05 (0.05, 0.06)
Monogamous Low Syllrep
Monogamous Medium/High Syllrep
0 (0, 0)
0.51 (0.43, 0.59)
0.01 (0, 0.01)
0.21 (0.18, 0.23)
0.08 (0.07, 0.08)
1.52 (1.39, 1.65)
Polygynous Low Syllrep
Polygynous Medium/High Syllrep
0.34 (0.31, 0.37)
Thresholds 10 – 33 (24)
Mean # iterations significant: 15.2/20
0 (0, 0)
Monogamous Low/Medium Syllrep
Monogamous Medium/High Syllrep
0 (0, 0)
0.43 (0.39, 0.47)
0.08 (0.07, 0.09)
0.24 (0.2, 0.28)
0.09 (0.08, 0.1)
0.25 (0.21, 0.29)
Polygynous Low/Medium Syllrep
Polygynous Medium/High Syllrep
0.19 (0.17, 0.22)
Thresholds 33 – 2400 (23)
Mean # iterations significant: 5/20
0.01 (0.01, 0.02)
Monogamous Low/Medium Syllrep
Monogamous High Syllrep
0.32 (0.27, 0.37)
0.29 (0.27, 0.32)
0.07 (0.07, 0.08)
0.34 (0.27, 0.41)
0.04 (0.03, 0.05)
0.07 (0.06, 0.07)
Polygynous Low/Medium Syllrep
Polygynous High Syllrep
0.6 (0.52, 0.67)
PolygynySyllrep Removed: Sturnidae; Original threshold range: 1 – 2400 (71)
p−value
0.0 0.2 0.4 0.6 0.8 1.0
1 5 10 50 100 500
High/Low Threshold: Syllrep
Removed: Acrocephalidae
Thresholds 1 – 10 (23)
Mean # iterations significant: 14.7/20
0.05 (0.05, 0.05)
Monogamous Low Syllrep
Monogamous Medium/High Syllrep
0 (0, 0)
0.51 (0.41, 0.61)
0.01 (0.01, 0.02)
0.17 (0.14, 0.19)
0.05 (0.05, 0.06)
1.53 (1.4, 1.66)
Polygynous Low Syllrep
Polygynous Medium/High Syllrep
0.42 (0.38, 0.46)
Thresholds 10 – 33 (22)
Mean # iterations significant: 12.5/20
0 (0, 0)
Monogamous Low/Medium Syllrep
Monogamous Medium/High Syllrep
0.01 (0.01, 0.01)
0.36 (0.31, 0.41)
0.07 (0.06, 0.08)
0.19 (0.16, 0.22)
0.05 (0.04, 0.06)
0.38 (0.27, 0.48)
Polygynous Low/Medium Syllrep
Polygynous Medium/High Syllrep
0.33 (0.24, 0.42)
Thresholds 33 – 2400 (22)
Mean # iterations significant: 3.3/20
0.02 (0.02, 0.02)
Monogamous Low/Medium Syllrep
Monogamous High Syllrep
0.39 (0.33, 0.46)
0.23 (0.2, 0.26)
0.06 (0.05, 0.06)
0.37 (0.27, 0.48)
0.02 (0.01, 0.03)
0.06 (0.05, 0.07)
Polygynous Low/Medium Syllrep
Polygynous High Syllrep
0.6 (0.5, 0.71)
PolygynySyllrep Removed: Acrocephalidae; Original threshold range: 1 – 2400 (68)
p−value
0.0 0.2 0.4 0.6 0.8 1.0
1 5 10 50 100 500
High/Low Threshold: Syllrep
Removed: Alaudidae
Thresholds 1 – 10 (23)
Mean # iterations significant: 16.4/20
0.05 (0.05, 0.06)
Monogamous Low Syllrep
Monogamous Medium/High Syllrep
0 (0, 0.01)
0.45 (0.39, 0.51)
0.01 (0, 0.01)
0.22 (0.2, 0.25)
0.09 (0.08, 0.09)
1.38 (1.27, 1.49)
Polygynous Low Syllrep
Polygynous Medium/High Syllrep
0.3 (0.28, 0.33)
Thresholds 10 – 33 (24)
Mean # iterations significant: 16.8/20
0 (0, 0)
Monogamous Low/Medium Syllrep
Monogamous Medium/High Syllrep
0 (0, 0)
0.42 (0.38, 0.46)
0.08 (0.07, 0.09)
0.25 (0.21, 0.29)
0.11 (0.09, 0.12)
0.22 (0.18, 0.25)
Polygynous Low/Medium Syllrep
Polygynous Medium/High Syllrep
0.16 (0.14, 0.18)
Thresholds 33 – 2400 (24)
Mean # iterations significant: 5.3/20
0.01 (0.01, 0.02)
Monogamous Low/Medium Syllrep
Monogamous High Syllrep
0.33 (0.27, 0.39)
0.33 (0.3, 0.36)
0.09 (0.08, 0.09)
0.35 (0.27, 0.43)
0.05 (0.03, 0.06)
0.05 (0.04, 0.07)
Polygynous Low/Medium Syllrep
Polygynous High Syllrep
0.54 (0.44, 0.64)
PolygynySyllrep Removed: Alaudidae; Original threshold range: 1 – 2400 (72)
p−value
0.0 0.2 0.4 0.6 0.8 1.0
1 5 10 50 100 500
High/Low Threshold: Syllrep
Removed: Cardinalidae
Thresholds 1 – 10 (22)
Mean # iterations significant: 16.1/20
0.05 (0.05, 0.06)
Monogamous Low Syllrep
Monogamous Medium/High Syllrep
0 (0, 0)
0.55 (0.47, 0.62)
0 (0, 0.01)
0.18 (0.16, 0.19)
0.07 (0.07, 0.08)
1.62 (1.48, 1.76)
Polygynous Low Syllrep
Polygynous Medium/High Syllrep
0.27 (0.24, 0.29)
Thresholds 10 – 33 (22)
Mean # iterations significant: 16.4/20
0 (0, 0)
Monogamous Low/Medium Syllrep
Monogamous Medium/High Syllrep
0 (0, 0)
0.4 (0.36, 0.44)
0.07 (0.06, 0.08)
0.24 (0.19, 0.29)
0.09 (0.08, 0.11)
0.25 (0.21, 0.3)
Polygynous Low/Medium Syllrep
Polygynous Medium/High Syllrep
0.17 (0.15, 0.19)
Thresholds 33 – 2400 (25)
Mean # iterations significant: 4/20
0.02 (0.02, 0.02)
Monogamous Low/Medium Syllrep
Monogamous High Syllrep
0.36 (0.31, 0.42)
0.32 (0.28, 0.35)
0.08 (0.07, 0.09)
0.32 (0.24, 0.39)
0.04 (0.03, 0.05)
0.05 (0.05, 0.06)
Polygynous Low/Medium Syllrep
Polygynous High Syllrep
0.53 (0.46, 0.6)
PolygynySyllrep Removed: Cardinalidae; Original threshold range: 1 – 2400 (70)
p−value
0.0 0.2 0.4 0.6 0.8 1.0
1 5 10 50 100 500
High/Low Threshold: Syllrep
Removed: Certhiidae
Thresholds 1 – 10 (23)
Mean # iterations significant: 15.8/20
0.05 (0.05, 0.05)
Monogamous Low Syllrep
Monogamous Medium/High Syllrep
0 (0, 0)
0.5 (0.42, 0.58)
0.01 (0.01, 0.02)
0.24 (0.21, 0.28)
0.09 (0.08, 0.1)
1.51 (1.38, 1.64)
Polygynous Low Syllrep
Polygynous Medium/High Syllrep
0.31 (0.28, 0.33)
Thresholds 10 – 33 (24)
Mean # iterations significant: 16.2/20
0 (0, 0)
Monogamous Low/Medium Syllrep
Monogamous Medium/High Syllrep
0 (0, 0)
0.42 (0.38, 0.47)
0.08 (0.07, 0.09)
0.26 (0.23, 0.29)
0.1 (0.09, 0.11)
0.21 (0.17, 0.24)
Polygynous Low/Medium Syllrep
Polygynous Medium/High Syllrep
0.15 (0.13, 0.16)
Thresholds 33 – 2400 (25)
Mean # iterations significant: 4.3/20
0.02 (0.01, 0.02)
Monogamous Low/Medium Syllrep
Monogamous High Syllrep
0.35 (0.29, 0.41)
0.3 (0.27, 0.33)
0.08 (0.07, 0.09)
0.32 (0.25, 0.39)
0.04 (0.03, 0.06)
0.06 (0.05, 0.06)
Polygynous Low/Medium Syllrep
Polygynous High Syllrep
0.56 (0.49, 0.64)
PolygynySyllrep Removed: Certhiidae; Original threshold range: 1 – 2400 (73)
p−value
0.0 0.2 0.4 0.6 0.8 1.0
1 5 10 50 100 500
High/Low Threshold: Syllrep

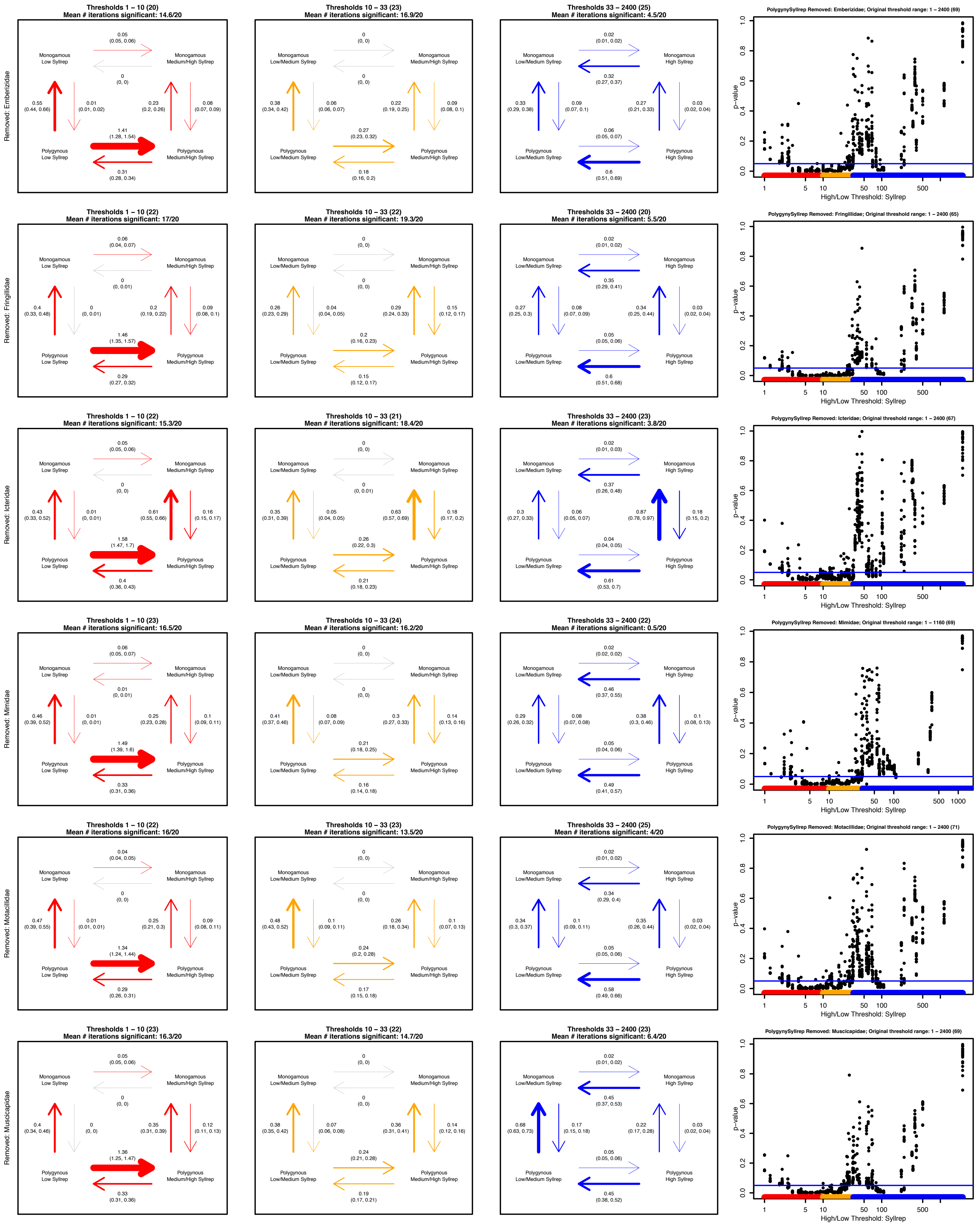

Removed: Emberizidae
Thresholds 1 – 10 (20)
Mean # iterations significant: 14.6/20
Thresholds 10 – 33 (23)
Mean # iterations significant: 16.9/20
Thresholds 33 – 2400 (25)
Mean # iterations significant: 4.5/20
PolygynySyllrep Removed: Emberizidae; Original threshold range: 1 – 2400 (69)
Removed: Fringillidae
Thresholds 1 – 10 (22)
Mean # iterations significant: 17/20
Thresholds 10 – 33 (22)
Mean # iterations significant: 19.3/20
Thresholds 33 – 2400 (20)
Mean # iterations significant: 5.5/20
PolygynySyllrep Removed: Fringillidae; Original threshold range: 1 – 2400 (65)
Removed: Icteridae
Thresholds 1 – 10 (22)
Mean # iterations significant: 15.3/20
Thresholds 10 – 33 (21)
Mean # iterations significant: 18.4/20
Thresholds 33 – 2400 (23)
Mean # iterations significant: 3.8/20
PolygynySyllrep Removed: Icteridae; Original threshold range: 1 – 2400 (67)
Removed: Mimidae
Thresholds 1 – 10 (23)
Mean # iterations significant: 16.5/20
Thresholds 10 – 33 (24)
Mean # iterations significant: 16.2/20
Thresholds 33 – 2400 (22)
Mean # iterations significant: 0.5/20
PolygynySyllrep Removed: Mimidae; Original threshold range: 1 – 1160 (69)
Removed: Motacillidae
Thresholds 1 – 10 (22)
Mean # iterations significant: 16/20
Thresholds 10 – 33 (23)
Mean # iterations significant: 13.5/20
Thresholds 33 – 2400 (25)
Mean # iterations significant: 4/20
PolygynySyllrep Removed: Motacillidae; Original threshold range: 1 – 2400 (71)
Removed: Muscicapidae
Thresholds 1 – 10 (23)
Mean # iterations significant: 16.3/20
Thresholds 10 – 33 (22)
Mean # iterations significant: 14.7/20
Thresholds 33 – 2400 (23)
Mean # iterations significant: 6.4/20
PolygynySyllrep Removed: Muscicapidae; Original threshold range: 1 – 2400 (69)
Monogamous
Low Syllrep
Monogamous
Medium/High Syllrep
Monogamous
Low/Medium Syllrep
Monogamous
High Syllrep
Polygynous
Low Syllrep
Polygynous
Medium/High Syllrep
Polygynous
Low/Medium Syllrep
Polygynous
High Syllrep
p–value
High/Low Threshold: Syllrep

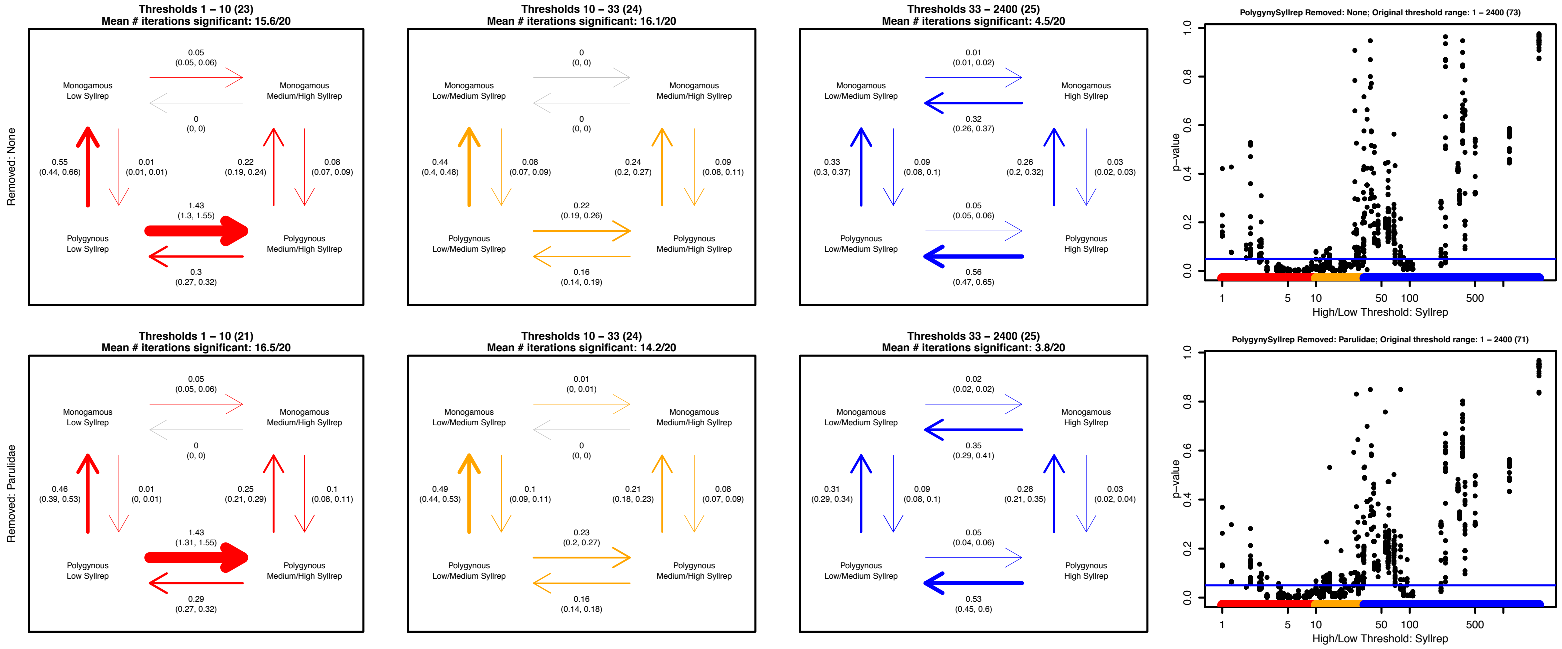
Removed: None
Thresholds 1 – 10 (23)
Mean # iterations significant: 15.6/20
Monogamous Low Syllrep
Monogamous Medium/High Syllrep
Polygynous Low Syllrep
Polygynous Medium/High Syllrep
0.05 (0.05, 0.06)
0 (0, 0)
0.55 (0.44, 0.66)
0.01 (0.01, 0.01)
0.22 (0.19, 0.24)
0.08 (0.07, 0.09)
1.43 (1.3, 1.55)
0.3 (0.27, 0.32)
Thresholds 10 – 33 (24)
Mean # iterations significant: 16.1/20
Monogamous Low/Medium Syllrep
Monogamous Medium/High Syllrep
Polygynous Low/Medium Syllrep
Polygynous Medium/High Syllrep
0 (0, 0)
0 (0, 0)
0.44 (0.4, 0.48)
0.08 (0.07, 0.09)
0.24 (0.2, 0.27)
0.09 (0.08, 0.11)
0.22 (0.19, 0.26)
0.16 (0.14, 0.19)
Thresholds 33 – 2400 (25)
Mean # iterations significant: 4.5/20
Monogamous Low/Medium Syllrep
Monogamous High Syllrep
Polygynous Low/Medium Syllrep
Polygynous High Syllrep
0.01 (0.01, 0.02)
0.32 (0.26, 0.37)
0.33 (0.3, 0.37)
0.09 (0.08, 0.1)
0.26 (0.2, 0.32)
0.03 (0.02, 0.03)
0.05 (0.05, 0.06)
0.56 (0.47, 0.65)
PolygynySyllrep Removed: None; Original threshold range: 1 – 2400 (73)
p−value
0.0 0.2 0.4 0.6 0.8 1.0
1 5 10 50 100 500
High/Low Threshold: Syllrep
Removed: Parulidae
Thresholds 1 – 10 (21)
Mean # iterations significant: 16.5/20
Monogamous Low Syllrep
Monogamous Medium/High Syllrep
Polygynous Low Syllrep
Polygynous Medium/High Syllrep
0.05 (0.05, 0.06)
0 (0, 0)
0.46 (0.39, 0.53)
0.01 (0, 0.01)
0.25 (0.21, 0.29)
0.1 (0.08, 0.11)
1.43 (1.31, 1.55)
0.29 (0.27, 0.32)
Thresholds 10 – 33 (24)
Mean # iterations significant: 14.2/20
Monogamous Low/Medium Syllrep
Monogamous Medium/High Syllrep
Polygynous Low/Medium Syllrep
Polygynous Medium/High Syllrep
0.01 (0, 0.01)
0 (0, 0)
0.49 (0.44, 0.53)
0.1 (0.09, 0.11)
0.21 (0.18, 0.23)
0.08 (0.07, 0.09)
0.23 (0.2, 0.27)
0.16 (0.14, 0.18)
Thresholds 33 – 2400 (25)
Mean # iterations significant: 3.8/20
Monogamous Low/Medium Syllrep
Monogamous High Syllrep
Polygynous Low/Medium Syllrep
Polygynous High Syllrep
0.02 (0.02, 0.02)
0.35 (0.29, 0.41)
0.31 (0.29, 0.34)
0.09 (0.08, 0.1)
0.28 (0.21, 0.35)
0.03 (0.02, 0.04)
0.05 (0.04, 0.06)
0.53 (0.45, 0.6)
PolygynySyllrep Removed: Parulidae; Original threshold range: 1 – 2400 (71)
p−value
0.0 0.2 0.4 0.6 0.8 1.0
1 5 10 50 100 500
High/Low Threshold: Syllrep

Supplementary Figure 31 (following pages): Jackknife analysis of correlated evolution between EPP and syllable repertoire size with Bayestraits. We removed one family at a time and repeated our BayesTraits analysis to detect correlated evolution between syllable repertoire size and EPP. We found consistent results with the analysis of all families, shown in Fig. 6.

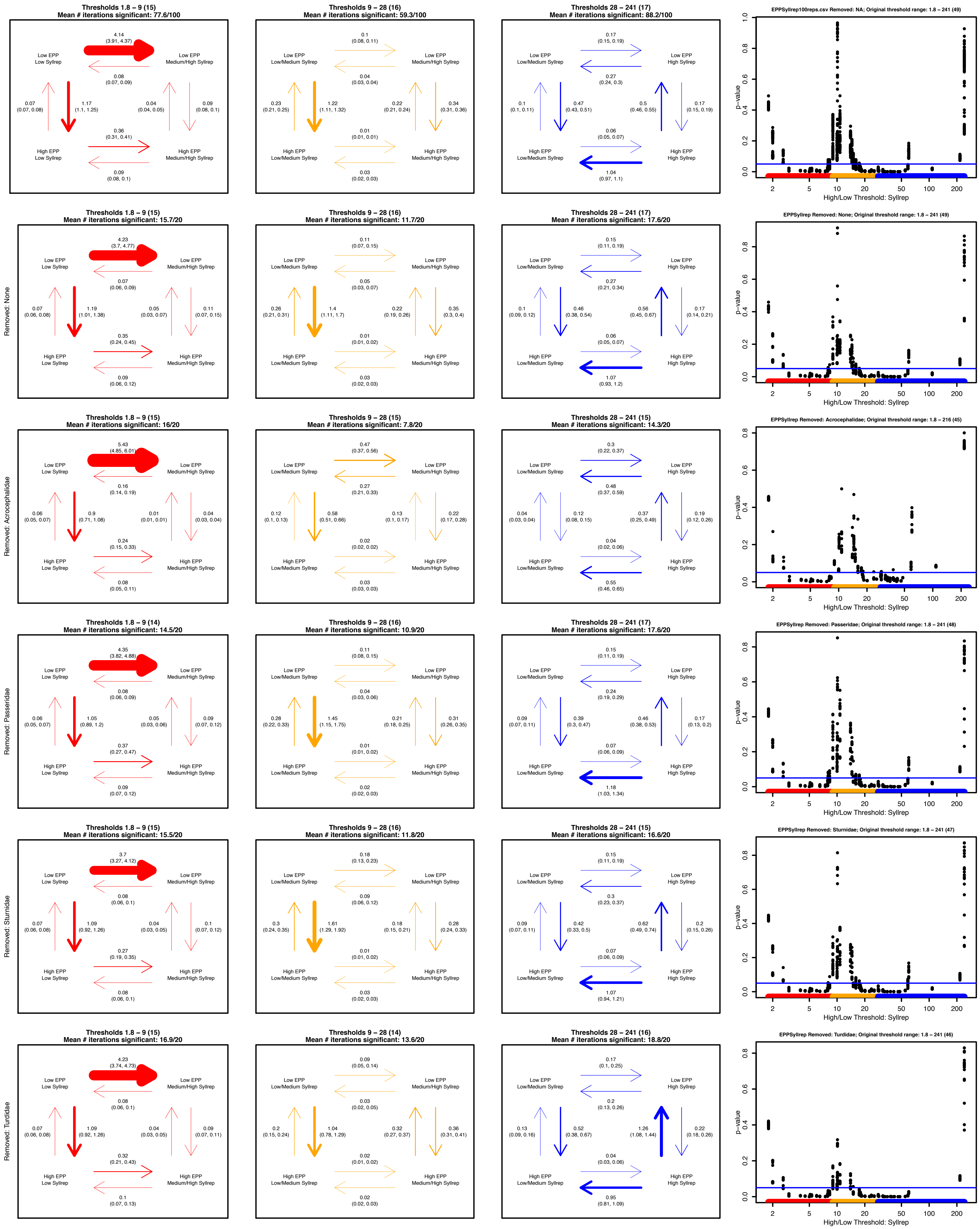
Thresholds 1.8 – 9 (15)
Mean # iterations significant: 77.6/100
4.14 (3.91, 4.37)
Low EPP Low Syllrep
Low EPP Medium/High Syllrep
0.08 (0.07, 0.09)
0.07 (0.07, 0.08)
1.17 (1.1, 1.25)
0.04 (0.04, 0.05)
0.09 (0.08, 0.1)
0.36 (0.31, 0.41)
High EPP Low Syllrep
High EPP Medium/High Syllrep
0.09 (0.08, 0.1)
Thresholds 9 – 28 (16)
Mean # iterations significant: 59.3/100
0.1 (0.08, 0.11)
Low EPP Low/Medium Syllrep
Low EPP Medium/High Syllrep
0.04 (0.03, 0.04)
0.23 (0.21, 0.25)
1.22 (1.11, 1.32)
0.22 (0.21, 0.24)
0.34 (0.31, 0.36)
0.01 (0.01, 0.01)
High EPP Low/Medium Syllrep
High EPP Medium/High Syllrep
0.03 (0.02, 0.03)
Thresholds 28 – 241 (17)
Mean # iterations significant: 88.2/100
0.17 (0.15, 0.19)
Low EPP Low/Medium Syllrep
Low EPP High Syllrep
0.27 (0.24, 0.3)
0.1 (0.1, 0.11)
0.47 (0.43, 0.51)
0.5 (0.46, 0.55)
0.17 (0.15, 0.19)
0.06 (0.05, 0.07)
High EPP Low/Medium Syllrep
High EPP High Syllrep
1.04 (0.97, 1.1)
EPPSyllrep100reps.csv Removed: NA; Original threshold range: 1.8 – 241 (49)
p–value
High/Low Threshold: Syllrep
Removed: None
Thresholds 1.8 – 9 (15)
Mean # iterations significant: 15.7/20
4.23 (3.7, 4.77)
0.07 (0.06, 0.09)
0.07 (0.06, 0.08)
1.19 (1.01, 1.38)
0.05 (0.03, 0.07)
0.11 (0.07, 0.15)
0.35 (0.24, 0.45)
0.09 (0.06, 0.12)
Thresholds 9 – 28 (16)
Mean # iterations significant: 11.7/20
0.11 (0.07, 0.15)
0.05 (0.03, 0.07)
0.26 (0.21, 0.31)
1.4 (1.11, 1.7)
0.22 (0.19, 0.26)
0.35 (0.3, 0.4)
0.01 (0.01, 0.02)
0.03 (0.02, 0.03)
Thresholds 28 – 241 (17)
Mean # iterations significant: 17.6/20
0.15 (0.11, 0.19)
0.27 (0.21, 0.34)
0.1 (0.09, 0.12)
0.46 (0.38, 0.54)
0.56 (0.45, 0.67)
0.17 (0.14, 0.21)
0.06 (0.05, 0.07)
1.07 (0.93, 1.2)
EPPSyllrep Removed: None; Original threshold range: 1.8 – 241 (49)
Removed: Acrocephalidae
Thresholds 1.8 – 9 (15)
Mean # iterations significant: 16/20
5.43 (4.85, 6.01)
0.16 (0.14, 0.19)
0.06 (0.05, 0.07)
0.9 (0.71, 1.08)
0.01 (0.01, 0.01)
0.04 (0.03, 0.04)
0.24 (0.15, 0.33)
0.08 (0.05, 0.11)
Thresholds 9 – 28 (15)
Mean # iterations significant: 7.8/20
0.47 (0.37, 0.56)
0.27 (0.21, 0.33)
0.12 (0.1, 0.13)
0.58 (0.51, 0.66)
0.13 (0.1, 0.17)
0.22 (0.17, 0.28)
0.02 (0.02, 0.02)
0.03 (0.03, 0.03)
Thresholds 28 – 241 (15)
Mean # iterations significant: 14.3/20
0.3 (0.22, 0.37)
0.48 (0.37, 0.59)
0.04 (0.03, 0.04)
0.12 (0.08, 0.15)
0.37 (0.25, 0.49)
0.19 (0.12, 0.26)
0.04 (0.02, 0.06)
0.55 (0.46, 0.65)
EPPSyllrep Removed: Acrocephalidae; Original threshold range: 1.8 – 216 (45)
Removed: Passeridae
Thresholds 1.8 – 9 (14)
Mean # iterations significant: 14.5/20
4.35 (3.82, 4.88)
0.08 (0.06, 0.09)
0.06 (0.05, 0.07)
1.05 (0.89, 1.2)
0.05 (0.03, 0.06)
0.09 (0.07, 0.12)
0.37 (0.27, 0.47)
0.09 (0.07, 0.12)
Thresholds 9 – 28 (16)
Mean # iterations significant: 10.9/20
0.11 (0.08, 0.15)
0.04 (0.03, 0.06)
0.28 (0.22, 0.33)
1.45 (1.15, 1.75)
0.21 (0.18, 0.25)
0.31 (0.26, 0.35)
0.01 (0.01, 0.02)
0.02 (0.02, 0.03)
Thresholds 28 – 241 (17)
Mean # iterations significant: 17.6/20
0.15 (0.11, 0.19)
0.24 (0.19, 0.29)
0.09 (0.07, 0.11)
0.39 (0.3, 0.47)
0.46 (0.38, 0.53)
0.17 (0.13, 0.2)
0.07 (0.06, 0.09)
1.18 (1.03, 1.34)
EPPSyllrep Removed: Passeridae; Original threshold range: 1.8 – 241 (48)
Removed: Sturnidae
Thresholds 1.8 – 9 (15)
Mean # iterations significant: 15.5/20
3.7 (3.27, 4.12)
0.08 (0.06, 0.1)
0.07 (0.06, 0.08)
1.09 (0.92, 1.26)
0.04 (0.03, 0.05)
0.1 (0.07, 0.12)
0.27 (0.19, 0.35)
0.08 (0.06, 0.1)
Thresholds 9 – 28 (16)
Mean # iterations significant: 11.8/20
0.18 (0.13, 0.23)
0.09 (0.06, 0.12)
0.3 (0.24, 0.35)
1.61 (1.29, 1.92)
0.18 (0.15, 0.21)
0.28 (0.24, 0.33)
0.01 (0.01, 0.02)
0.03 (0.02, 0.03)
Thresholds 28 – 241 (15)
Mean # iterations significant: 16.6/20
0.15 (0.11, 0.19)
0.3 (0.23, 0.37)
0.09 (0.07, 0.11)
0.42 (0.33, 0.5)
0.62 (0.49, 0.74)
0.2 (0.15, 0.26)
0.07 (0.06, 0.09)
1.07 (0.94, 1.21)
EPPSyllrep Removed: Sturnidae; Original threshold range: 1.8 – 241 (47)
Removed: Turdidae
Thresholds 1.8 – 9 (15)
Mean # iterations significant: 16.9/20
4.23 (3.74, 4.73)
0.08 (0.06, 0.1)
0.07 (0.06, 0.08)
1.09 (0.92, 1.26)
0.04 (0.03, 0.05)
0.09 (0.07, 0.11)
0.32 (0.21, 0.43)
0.1 (0.07, 0.13)
Thresholds 9 – 28 (14)
Mean # iterations significant: 13.6/20
0.09 (0.05, 0.14)
0.03 (0.02, 0.05)
0.2 (0.15, 0.24)
1.04 (0.78, 1.29)
0.32 (0.27, 0.37)
0.36 (0.31, 0.41)
0.02 (0.01, 0.02)
0.02 (0.02, 0.03)
Thresholds 28 – 241 (16)
Mean # iterations significant: 18.8/20
0.17 (0.1, 0.25)
0.2 (0.13, 0.26)
0.13 (0.09, 0.16)
0.52 (0.38, 0.67)
1.26 (1.08, 1.44)
0.22 (0.18, 0.26)
0.04 (0.03, 0.06)
0.95 (0.81, 1.09)
EPPSyllrep Removed: Turdidae; Original threshold range: 1.8 – 241 (46)

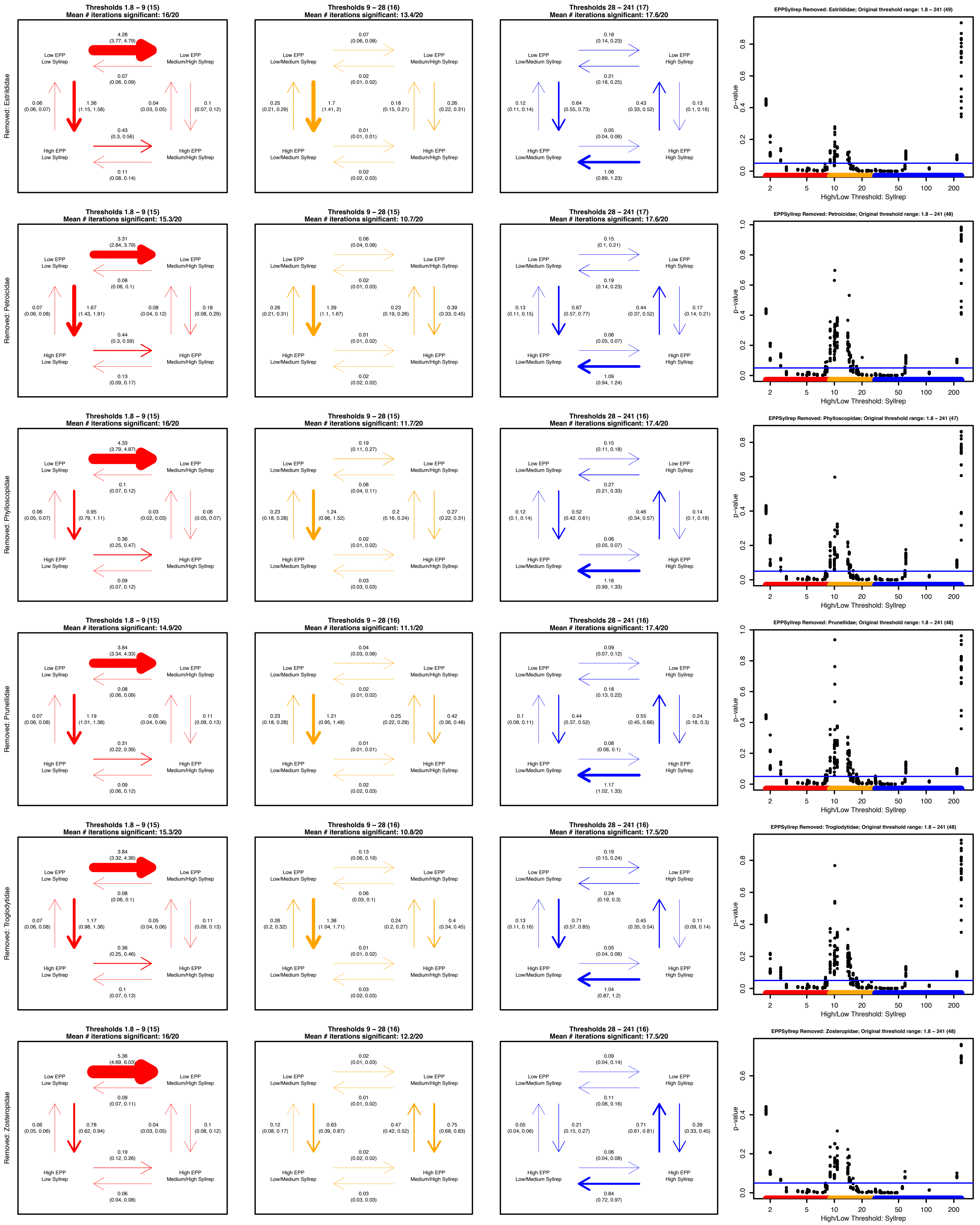
Removed: Estrildidae
Thresholds 1.8 – 9 (15)
Mean # iterations significant: 16/20
Thresholds 9 – 28 (16)
Mean # iterations significant: 13.4/20
Thresholds 28 – 241 (17)
Mean # iterations significant: 17.6/20
EPPSyllrep Removed: Estrildidae; Original threshold range: 1.8 – 241 (49)
Removed: Petroicidae
Thresholds 1.8 – 9 (15)
Mean # iterations significant: 15.3/20
Thresholds 9 – 28 (15)
Mean # iterations significant: 10.7/20
Thresholds 28 – 241 (17)
Mean # iterations significant: 17.6/20
EPPSyllrep Removed: Petroicidae; Original threshold range: 1.8 – 241 (48)
Removed: Phylloscopidae
Thresholds 1.8 – 9 (15)
Mean # iterations significant: 16/20
Thresholds 9 – 28 (15)
Mean # iterations significant: 11.7/20
Thresholds 28 – 241 (16)
Mean # iterations significant: 17.4/20
EPPSyllrep Removed: Phylloscopidae; Original threshold range: 1.8 – 241 (47)
Removed: Prunellidae
Thresholds 1.8 – 9 (15)
Mean # iterations significant: 14.9/20
Thresholds 9 – 28 (16)
Mean # iterations significant: 11.1/20
Thresholds 28 – 241 (16)
Mean # iterations significant: 17.4/20
EPPSyllrep Removed: Prunellidae; Original threshold range: 1.8 – 241 (48)
Removed: Troglodytidae
Thresholds 1.8 – 9 (15)
Mean # iterations significant: 15.3/20
Thresholds 9 – 28 (16)
Mean # iterations significant: 10.8/20
Thresholds 28 – 241 (16)
Mean # iterations significant: 17.5/20
EPPSyllrep Removed: Troglodytidae; Original threshold range: 1.8 – 241 (48)
Removed: Zosteropidae
Thresholds 1.8 – 9 (15)
Mean # iterations significant: 16/20
Thresholds 9 – 28 (16)
Mean # iterations significant: 12.2/20
Thresholds 28 – 241 (16)
Mean # iterations significant: 17.5/20
EPPSyllrep Removed: Zosteropidae; Original threshold range: 1.8 – 241 (48)
Low EPP Low Syllrep
Low EPP Medium/High Syllrep
High EPP Low Syllrep
High EPP Medium/High Syllrep
Low EPP Low/Medium Syllrep
High EPP Low/Medium Syllrep
Low EPP High Syllrep
High EPP High Syllrep
p−value
High/Low Threshold: Syllrep

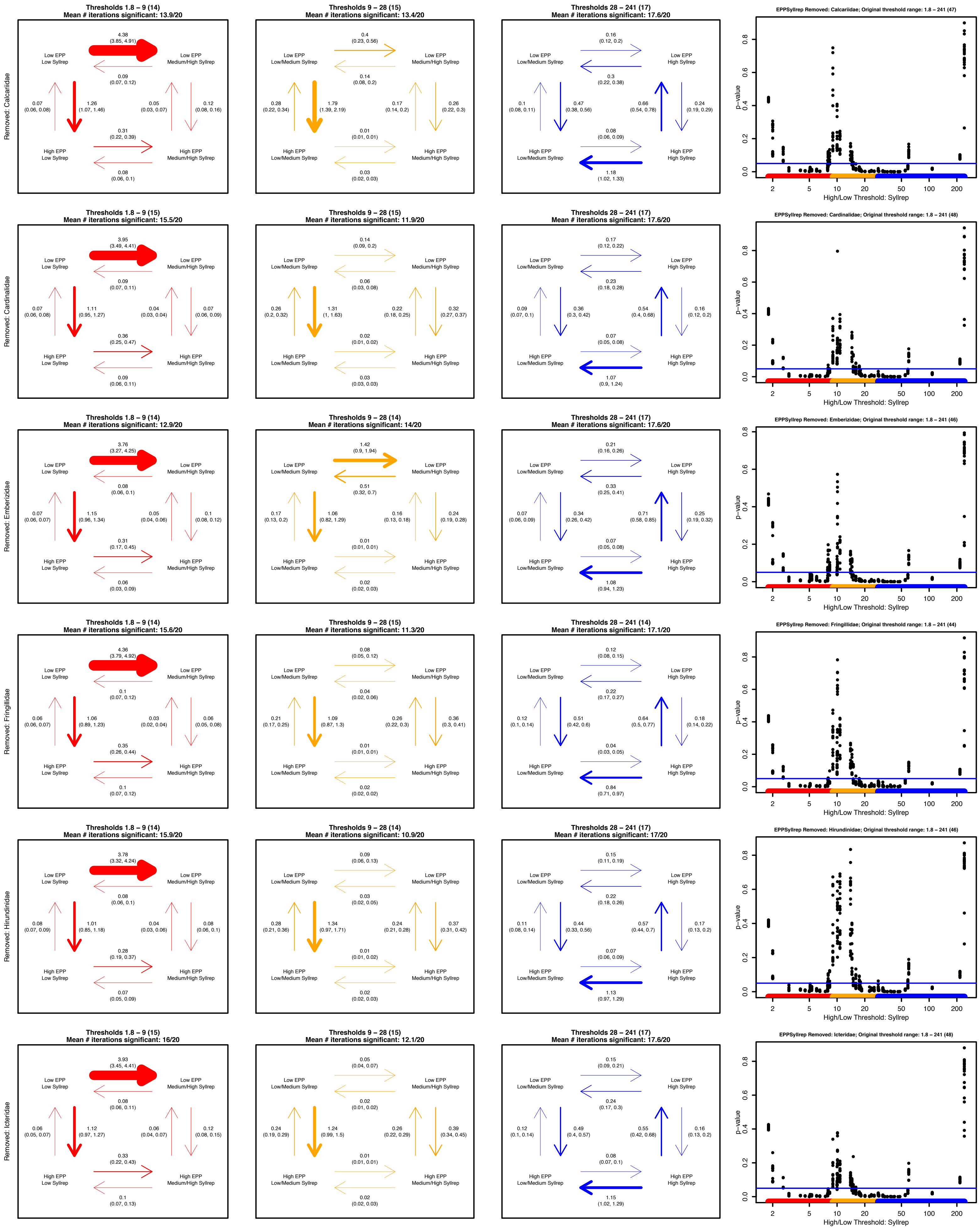
Removed: Calcariidae
Thresholds 1.8 – 9 (14)
Mean # iterations significant: 13.9/20
4.38 (3.85, 4.91)
Low EPP Low Syllrep
Low EPP Medium/High Syllrep
0.09 (0.07, 0.12)
0.07 (0.06, 0.08)
1.26 (1.07, 1.46)
0.05 (0.03, 0.07)
0.12 (0.08, 0.16)
0.31 (0.22, 0.39)
High EPP Low Syllrep
High EPP Medium/High Syllrep
0.08 (0.06, 0.1)
Thresholds 9 – 28 (15)
Mean # iterations significant: 13.4/20
0.4 (0.23, 0.56)
Low EPP Low/Medium Syllrep
Low EPP Medium/High Syllrep
0.14 (0.08, 0.2)
0.28 (0.22, 0.34)
1.79 (1.39, 2.19)
0.17 (0.14, 0.2)
0.26 (0.22, 0.3)
0.01 (0.01, 0.01)
High EPP Low/Medium Syllrep
High EPP Medium/High Syllrep
0.03 (0.02, 0.03)
Thresholds 28 – 241 (17)
Mean # iterations significant: 17.6/20
0.16 (0.12, 0.2)
Low EPP Low/Medium Syllrep
Low EPP High Syllrep
0.3 (0.22, 0.38)
0.1 (0.08, 0.11)
0.47 (0.38, 0.56)
0.66 (0.54, 0.78)
0.24 (0.19, 0.29)
0.08 (0.06, 0.09)
High EPP Low/Medium Syllrep
High EPP High Syllrep
1.18 (1.02, 1.33)
EPPSyllrep Removed: Calcariidae; Original threshold range: 1.8 – 241 (47)
p−value
High/Low Threshold: Syllrep
Removed: Cardinalidae
Thresholds 1.8 – 9 (15)
Mean # iterations significant: 15.5/20
3.95 (3.49, 4.41)
0.09 (0.07, 0.11)
0.07 (0.06, 0.08)
1.11 (0.95, 1.27)
0.04 (0.03, 0.04)
0.07 (0.06, 0.09)
0.36 (0.25, 0.47)
0.09 (0.06, 0.11)
Thresholds 9 – 28 (15)
Mean # iterations significant: 11.9/20
0.14 (0.09, 0.2)
0.06 (0.03, 0.08)
0.26 (0.2, 0.32)
1.31 (1, 1.63)
0.22 (0.18, 0.25)
0.32 (0.27, 0.37)
0.02 (0.01, 0.02)
0.03 (0.03, 0.03)
Thresholds 28 – 241 (17)
Mean # iterations significant: 17.6/20
0.17 (0.12, 0.22)
0.23 (0.18, 0.28)
0.09 (0.07, 0.1)
0.36 (0.3, 0.42)
0.54 (0.4, 0.68)
0.16 (0.12, 0.2)
0.07 (0.05, 0.08)
1.07 (0.9, 1.24)
EPPSyllrep Removed: Cardinalidae; Original threshold range: 1.8 – 241 (48)
Removed: Emberizidae
Thresholds 1.8 – 9 (14)
Mean # iterations significant: 12.9/20
3.76 (3.27, 4.25)
0.08 (0.06, 0.1)
0.07 (0.06, 0.07)
1.15 (0.96, 1.34)
0.05 (0.04, 0.06)
0.1 (0.08, 0.12)
0.31 (0.17, 0.45)
0.06 (0.03, 0.09)
Thresholds 9 – 28 (14)
Mean # iterations significant: 14/20
1.42 (0.9, 1.94)
0.51 (0.32, 0.7)
0.17 (0.13, 0.2)
1.06 (0.82, 1.29)
0.16 (0.13, 0.18)
0.24 (0.19, 0.28)
0.01 (0.01, 0.01)
0.02 (0.02, 0.03)
Thresholds 28 – 241 (17)
Mean # iterations significant: 17.6/20
0.21 (0.16, 0.26)
0.33 (0.25, 0.41)
0.07 (0.06, 0.09)
0.34 (0.26, 0.42)
0.71 (0.58, 0.85)
0.25 (0.19, 0.32)
0.07 (0.05, 0.08)
1.08 (0.94, 1.23)
EPPSyllrep Removed: Emberizidae; Original threshold range: 1.8 – 241 (46)
Removed: Fringillidae
Thresholds 1.8 – 9 (14)
Mean # iterations significant: 15.6/20
4.36 (3.79, 4.92)
0.1 (0.07, 0.12)
0.06 (0.06, 0.07)
1.06 (0.89, 1.23)
0.03 (0.02, 0.04)
0.06 (0.05, 0.08)
0.35 (0.26, 0.44)
0.1 (0.07, 0.12)
Thresholds 9 – 28 (15)
Mean # iterations significant: 11.3/20
0.08 (0.05, 0.12)
0.04 (0.02, 0.06)
0.21 (0.17, 0.25)
1.09 (0.87, 1.3)
0.26 (0.22, 0.3)
0.36 (0.3, 0.41)
0.01 (0.01, 0.01)
0.02 (0.02, 0.02)
Thresholds 28 – 241 (14)
Mean # iterations significant: 17.1/20
0.12 (0.08, 0.15)
0.22 (0.17, 0.27)
0.12 (0.1, 0.14)
0.51 (0.42, 0.6)
0.64 (0.5, 0.77)
0.18 (0.14, 0.22)
0.04 (0.03, 0.05)
0.84 (0.71, 0.97)
EPPSyllrep Removed: Fringillidae; Original threshold range: 1.8 – 241 (44)
Removed: Hirundinidae
Thresholds 1.8 – 9 (14)
Mean # iterations significant: 15.9/20
3.78 (3.32, 4.24)
0.08 (0.06, 0.1)
0.08 (0.07, 0.09)
1.01 (0.85, 1.18)
0.04 (0.03, 0.06)
0.08 (0.06, 0.1)
0.28 (0.19, 0.37)
0.07 (0.05, 0.09)
Thresholds 9 – 28 (14)
Mean # iterations significant: 10.9/20
0.09 (0.06, 0.13)
0.03 (0.02, 0.05)
0.28 (0.21, 0.36)
1.34 (0.97, 1.71)
0.24 (0.21, 0.28)
0.37 (0.31, 0.42)
0.01 (0.01, 0.02)
0.02 (0.02, 0.03)
Thresholds 28 – 241 (17)
Mean # iterations significant: 17/20
0.15 (0.11, 0.19)
0.22 (0.18, 0.26)
0.11 (0.08, 0.14)
0.44 (0.33, 0.56)
0.57 (0.44, 0.7)
0.17 (0.13, 0.2)
0.07 (0.06, 0.09)
1.13 (0.97, 1.29)
EPPSyllrep Removed: Hirundinidae; Original threshold range: 1.8 – 241 (46)
Removed: Icteridae
Thresholds 1.8 – 9 (15)
Mean # iterations significant: 16/20
3.93 (3.45, 4.41)
0.08 (0.06, 0.11)
0.06 (0.05, 0.07)
1.12 (0.97, 1.27)
0.06 (0.04, 0.07)
0.12 (0.08, 0.15)
0.33 (0.22, 0.43)
0.1 (0.07, 0.13)
Thresholds 9 – 28 (15)
Mean # iterations significant: 12.1/20
0.05 (0.04, 0.07)
0.02 (0.01, 0.02)
0.24 (0.19, 0.29)
1.24 (0.99, 1.5)
0.26 (0.22, 0.29)
0.39 (0.34, 0.45)
0.01 (0.01, 0.01)
0.02 (0.02, 0.03)
Thresholds 28 – 241 (17)
Mean # iterations significant: 17.6/20
0.15 (0.09, 0.21)
0.24 (0.17, 0.3)
0.12 (0.1, 0.14)
0.49 (0.4, 0.57)
0.55 (0.42, 0.68)
0.16 (0.13, 0.2)
0.08 (0.07, 0.1)
1.15 (1.02, 1.29)
EPPSyllrep Removed: Icteridae; Original threshold range: 1.8 – 241 (48)

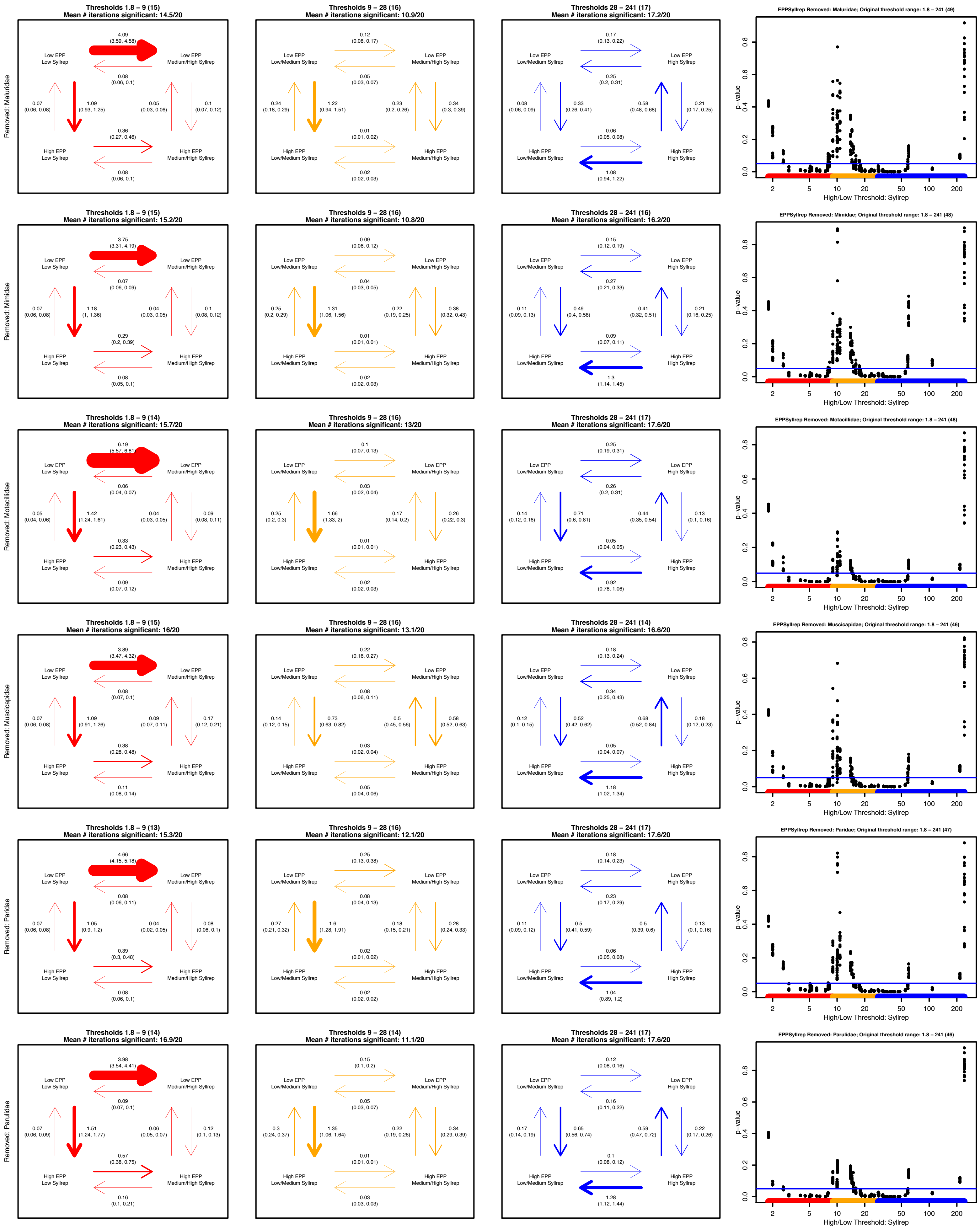

Removed: Maluridae
Thresholds 1.8 – 9 (15)
Mean # iterations significant: 14.5/20
Low EPP Low Syllrep
Low EPP Medium/High Syllrep
High EPP Low Syllrep
High EPP Medium/High Syllrep
4.09 (3.59, 4.58)
0.08 (0.06, 0.1)
0.07 (0.06, 0.08)
1.09 (0.93, 1.25)
0.05 (0.03, 0.06)
0.1 (0.07, 0.12)
0.36 (0.27, 0.46)
0.08 (0.06, 0.1)
Thresholds 9 – 28 (16)
Mean # iterations significant: 10.9/20
Low EPP Low/Medium Syllrep
Low EPP Medium/High Syllrep
High EPP Low/Medium Syllrep
High EPP Medium/High Syllrep
0.12 (0.08, 0.17)
0.05 (0.03, 0.07)
0.24 (0.18, 0.29)
1.22 (0.94, 1.51)
0.23 (0.2, 0.26)
0.34 (0.3, 0.39)
0.01 (0.01, 0.02)
0.02 (0.02, 0.03)
Thresholds 28 – 241 (17)
Mean # iterations significant: 17.2/20
Low EPP Low/Medium Syllrep
Low EPP High Syllrep
High EPP Low/Medium Syllrep
High EPP High Syllrep
0.17 (0.13, 0.22)
0.25 (0.2, 0.31)
0.08 (0.06, 0.09)
0.33 (0.26, 0.41)
0.58 (0.48, 0.68)
0.21 (0.17, 0.25)
0.06 (0.05, 0.08)
1.08 (0.94, 1.22)
EPPSyllrep Removed: Maluridae; Original threshold range: 1.8 – 241 (49)
p−value
High/Low Threshold: Syllrep
Removed: Mimidae
Thresholds 1.8 – 9 (15)
Mean # iterations significant: 15.2/20
3.75 (3.31, 4.19)
0.07 (0.06, 0.09)
0.07 (0.06, 0.08)
1.18 (1, 1.36)
0.04 (0.03, 0.05)
0.1 (0.08, 0.12)
0.29 (0.2, 0.39)
0.08 (0.05, 0.1)
Thresholds 9 – 28 (16)
Mean # iterations significant: 10.8/20
0.09 (0.06, 0.12)
0.04 (0.03, 0.05)
0.25 (0.2, 0.29)
1.31 (1.06, 1.56)
0.22 (0.19, 0.25)
0.38 (0.32, 0.43)
0.01 (0.01, 0.01)
0.02 (0.02, 0.03)
Thresholds 28 – 241 (16)
Mean # iterations significant: 16.2/20
0.15 (0.12, 0.19)
0.27 (0.21, 0.33)
0.11 (0.09, 0.13)
0.49 (0.4, 0.58)
0.41 (0.32, 0.51)
0.21 (0.16, 0.25)
0.09 (0.07, 0.11)
1.3 (1.14, 1.45)
EPPSyllrep Removed: Mimidae; Original threshold range: 1.8 – 241 (48)
Removed: Motacillidae
Thresholds 1.8 – 9 (14)
Mean # iterations significant: 15.7/20
6.19 (5.57, 6.81)
0.06 (0.04, 0.07)
0.05 (0.04, 0.06)
1.42 (1.24, 1.61)
0.04 (0.03, 0.05)
0.09 (0.08, 0.11)
0.33 (0.23, 0.43)
0.09 (0.07, 0.12)
Thresholds 9 – 28 (16)
Mean # iterations significant: 13/20
0.1 (0.07, 0.13)
0.03 (0.02, 0.04)
0.25 (0.2, 0.3)
1.66 (1.33, 2)
0.17 (0.14, 0.2)
0.26 (0.22, 0.3)
0.01 (0.01, 0.01)
0.02 (0.02, 0.03)
Thresholds 28 – 241 (17)
Mean # iterations significant: 17.6/20
0.25 (0.19, 0.31)
0.26 (0.2, 0.31)
0.14 (0.12, 0.16)
0.71 (0.6, 0.81)
0.44 (0.35, 0.54)
0.13 (0.1, 0.16)
0.05 (0.04, 0.05)
0.92 (0.78, 1.06)
EPPSyllrep Removed: Motacillidae; Original threshold range: 1.8 – 241 (48)
Removed: Muscicapidae
Thresholds 1.8 – 9 (15)
Mean # iterations significant: 16/20
3.89 (3.47, 4.32)
0.08 (0.07, 0.1)
0.07 (0.06, 0.08)
1.09 (0.91, 1.26)
0.09 (0.07, 0.11)
0.17 (0.12, 0.21)
0.38 (0.28, 0.48)
0.11 (0.08, 0.14)
Thresholds 9 – 28 (16)
Mean # iterations significant: 13.1/20
0.22 (0.16, 0.27)
0.08 (0.06, 0.11)
0.14 (0.12, 0.15)
0.73 (0.63, 0.82)
0.5 (0.45, 0.56)
0.58 (0.52, 0.63)
0.03 (0.02, 0.04)
0.05 (0.04, 0.06)
Thresholds 28 – 241 (14)
Mean # iterations significant: 16.6/20
0.18 (0.13, 0.24)
0.34 (0.25, 0.43)
0.12 (0.1, 0.15)
0.52 (0.42, 0.62)
0.68 (0.52, 0.84)
0.18 (0.12, 0.23)
0.05 (0.04, 0.07)
1.18 (1.02, 1.34)
EPPSyllrep Removed: Muscicapidae; Original threshold range: 1.8 – 241 (46)
Removed: Paridae
Thresholds 1.8 – 9 (13)
Mean # iterations significant: 15.3/20
4.66 (4.15, 5.18)
0.08 (0.06, 0.11)
0.07 (0.06, 0.08)
1.05 (0.9, 1.2)
0.04 (0.02, 0.05)
0.08 (0.06, 0.1)
0.39 (0.3, 0.48)
0.08 (0.06, 0.1)
Thresholds 9 – 28 (16)
Mean # iterations significant: 12.1/20
0.25 (0.13, 0.38)
0.08 (0.04, 0.13)
0.27 (0.21, 0.32)
1.6 (1.28, 1.91)
0.18 (0.15, 0.21)
0.28 (0.24, 0.33)
0.02 (0.01, 0.02)
0.02 (0.02, 0.02)
Thresholds 28 – 241 (17)
Mean # iterations significant: 17.6/20
0.18 (0.14, 0.23)
0.23 (0.17, 0.29)
0.11 (0.09, 0.12)
0.5 (0.41, 0.59)
0.5 (0.39, 0.6)
0.13 (0.1, 0.16)
0.06 (0.05, 0.08)
1.04 (0.89, 1.2)
EPPSyllrep Removed: Paridae; Original threshold range: 1.8 – 241 (47)
Removed: Parulidae
Thresholds 1.8 – 9 (14)
Mean # iterations significant: 16.9/20
3.98 (3.54, 4.41)
0.09 (0.07, 0.1)
0.07 (0.06, 0.09)
1.51 (1.24, 1.77)
0.06 (0.05, 0.07)
0.12 (0.1, 0.13)
0.57 (0.38, 0.75)
0.16 (0.1, 0.21)
Thresholds 9 – 28 (14)
Mean # iterations significant: 11.1/20
0.15 (0.1, 0.2)
0.05 (0.03, 0.07)
0.3 (0.24, 0.37)
1.35 (1.06, 1.64)
0.22 (0.19, 0.26)
0.34 (0.29, 0.39)
0.01 (0.01, 0.01)
0.03 (0.03, 0.03)
Thresholds 28 – 241 (17)
Mean # iterations significant: 17.6/20
0.12 (0.08, 0.16)
0.16 (0.11, 0.22)
0.17 (0.14, 0.19)
0.65 (0.56, 0.74)
0.59 (0.47, 0.72)
0.22 (0.17, 0.26)
0.1 (0.08, 0.12)
1.28 (1.12, 1.44)
EPPSyllrep Removed: Parulidae; Original threshold range: 1.8 – 241 (46)

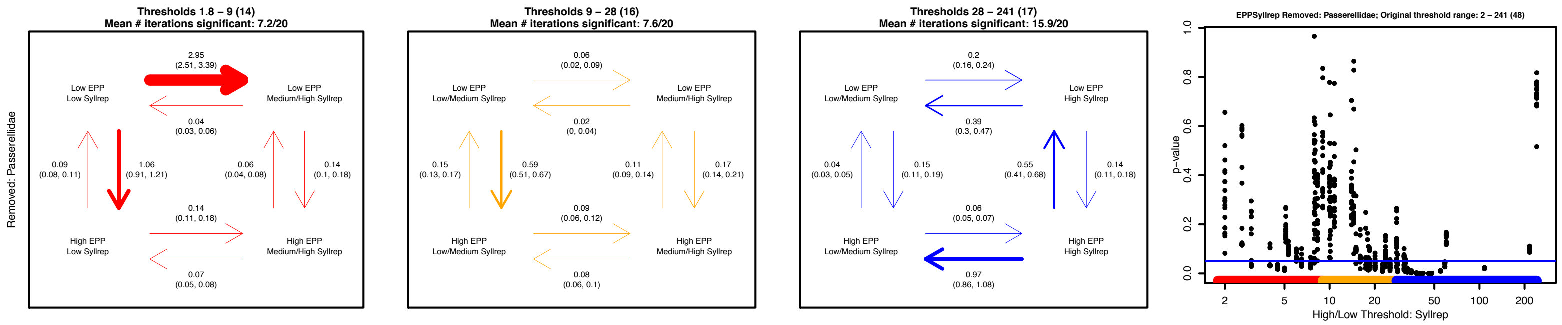

Thresholds 1.8 – 9 (14)
Mean # iterations significant: 7.2/20
Removed: Passerellidae
Low EPP
Low Syllrep
Low EPP
Medium/High Syllrep
High EPP
Low Syllrep
High EPP
Medium/High Syllrep
2.95
(2.51, 3.39)
0.04
(0.03, 0.06)
0.09
(0.08, 0.11)
1.06
(0.91, 1.21)
0.06
(0.04, 0.08)
0.14
(0.1, 0.18)
0.14
(0.11, 0.18)
0.07
(0.05, 0.08)
Thresholds 9 – 28 (16)
Mean # iterations significant: 7.6/20
Low EPP
Low/Medium Syllrep
Low EPP
Medium/High Syllrep
High EPP
Low/Medium Syllrep
High EPP
Medium/High Syllrep
0.06
(0.02, 0.09)
0.02
(0, 0.04)
0.15
(0.13, 0.17)
0.59
(0.51, 0.67)
0.11
(0.09, 0.14)
0.17
(0.14, 0.21)
0.09
(0.06, 0.12)
0.08
(0.06, 0.1)
Thresholds 28 – 241 (17)
Mean # iterations significant: 15.9/20
Low EPP
Low/Medium Syllrep
Low EPP
High Syllrep
High EPP
Low/Medium Syllrep
High EPP
High Syllrep
0.2
(0.16, 0.24)
0.39
(0.3, 0.47)
0.04
(0.03, 0.05)
0.15
(0.11, 0.19)
0.55
(0.41, 0.68)
0.14
(0.11, 0.18)
0.06
(0.05, 0.07)
0.97
(0.86, 1.08)
EPPSyllrep Removed: Passerellidae; Original threshold range: 2 – 241 (48)
p–value
0.0
0.2
0.4
0.6
0.8
1.0
2
5
10
20
50
100
200
High/Low Threshold: Syllrep

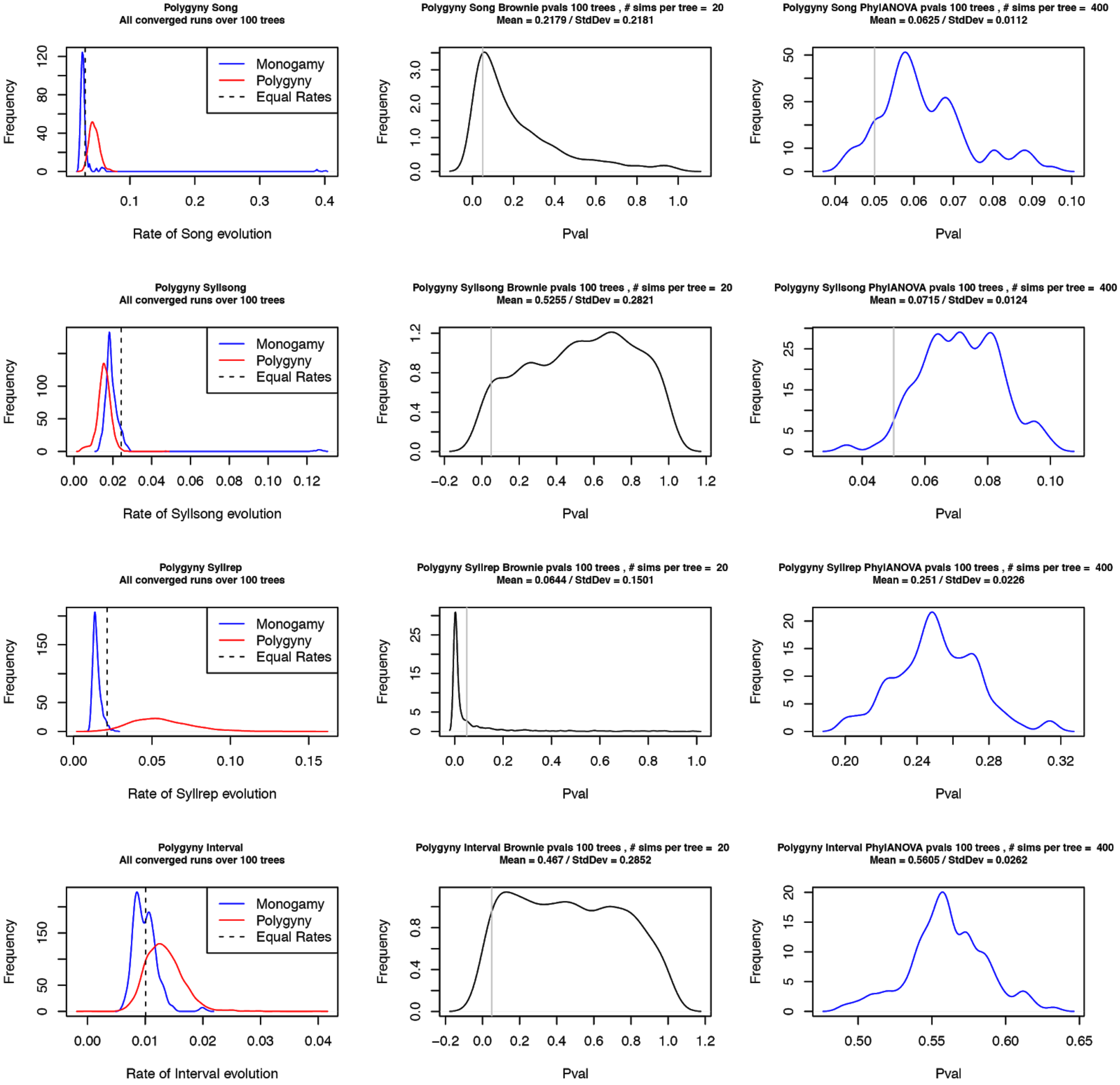
Polygyny Song
All converged runs over 100 trees
Monogamy
Polygyny
Equal Rates
Frequency
Rate of Song evolution
Polygyny Song Brownie pvals 100 trees , # sims per tree = 20
Mean = 0.2179 / StdDev = 0.2181
Frequency
Pval
Polygyny Song PhylANOVA pvals 100 trees , # sims per tree = 400
Mean = 0.0625 / StdDev = 0.0112
Frequency
Pval
Polygyny Syllsong
All converged runs over 100 trees
Monogamy
Polygyny
Equal Rates
Frequency
Rate of Syllsong evolution
Polygyny Syllsong Brownie pvals 100 trees , # sims per tree = 20
Mean = 0.5255 / StdDev = 0.2821
Frequency
Pval
Polygyny Syllsong PhylANOVA pvals 100 trees , # sims per tree = 400
Mean = 0.0715 / StdDev = 0.0124
Frequency
Pval
Polygyny Syllrep
All converged runs over 100 trees
Monogamy
Polygyny
Equal Rates
Frequency
Rate of Syllrep evolution
Polygyny Syllrep Brownie pvals 100 trees , # sims per tree = 20
Mean = 0.0644 / StdDev = 0.1501
Frequency
Pval
Polygyny Syllrep PhylANOVA pvals 100 trees , # sims per tree = 400
Mean = 0.251 / StdDev = 0.0226
Frequency
Pval
Polygyny Interval
All converged runs over 100 trees
Monogamy
Polygyny
Equal Rates
Frequency
Rate of Interval evolution
Polygyny Interval Brownie pvals 100 trees , # sims per tree = 20
Mean = 0.467 / StdDev = 0.2852
Frequency
Pval
Polygyny Interval PhylANOVA pvals 100 trees , # sims per tree = 400
Mean = 0.5605 / StdDev = 0.0262
Frequency
Pval

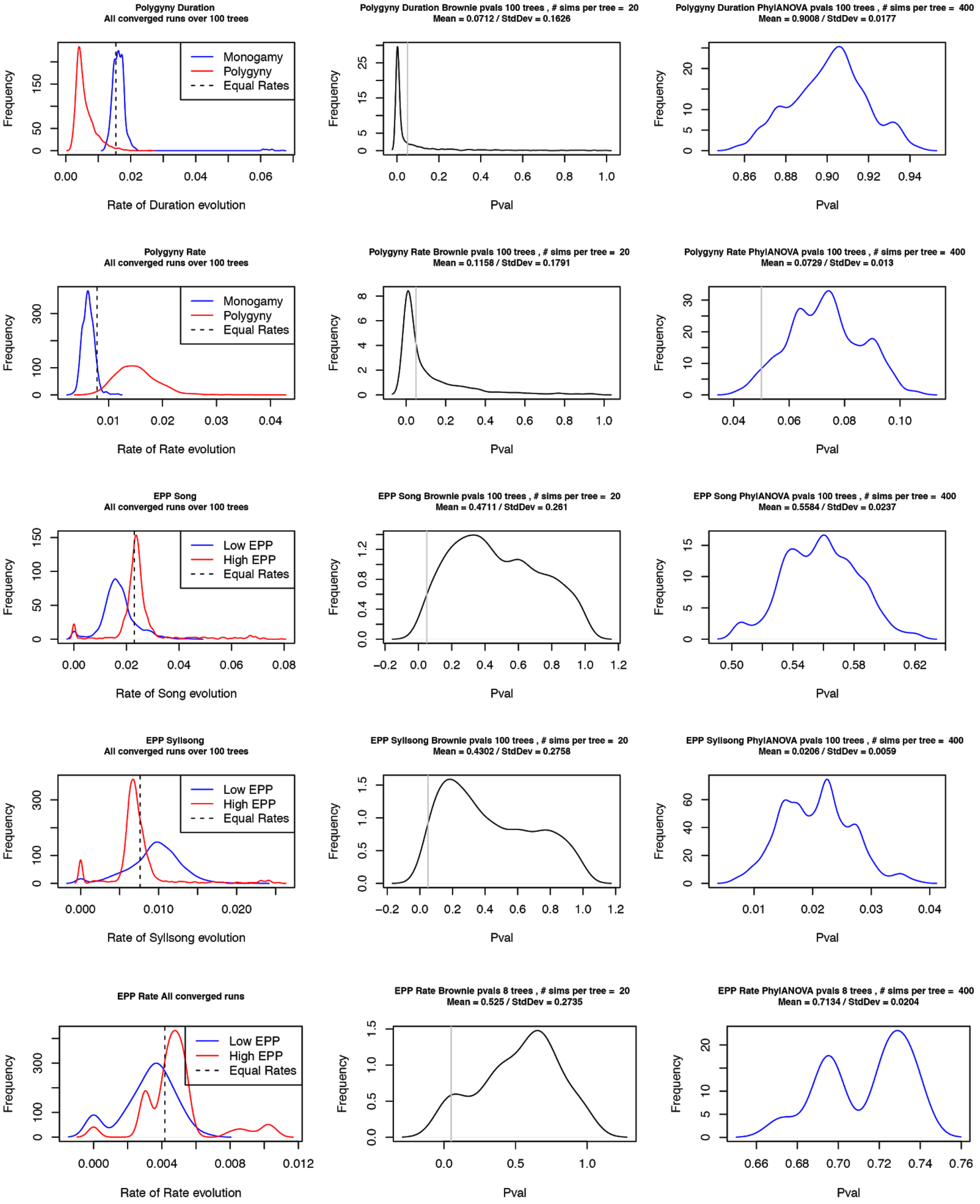

Polygyny Duration
All converged runs over 100 trees
Monogamy
Polygyny
Equal Rates
Frequency
Rate of Duration evolution
Polygyny Duration Brownie pvals 100 trees , # sims per tree = 20
Mean = 0.0712 / StdDev = 0.1626
Frequency
Pval
Polygyny Duration PhylANOVA pvals 100 trees , # sims per tree = 400
Mean = 0.9008 / StdDev = 0.0177
Frequency
Pval
Polygyny Rate
All converged runs over 100 trees
Monogamy
Polygyny
Equal Rates
Frequency
Rate of Rate evolution
Polygyny Rate Brownie pvals 100 trees , # sims per tree = 20
Mean = 0.1158 / StdDev = 0.1791
Frequency
Pval
Polygyny Rate PhylANOVA pvals 100 trees , # sims per tree = 400
Mean = 0.0729 / StdDev = 0.013
Frequency
Pval
EPP Song
All converged runs over 100 trees
Low EPP
High EPP
Equal Rates
Frequency
Rate of Song evolution
EPP Song Brownie pvals 100 trees , # sims per tree = 20
Mean = 0.4711 / StdDev = 0.261
Frequency
Pval
EPP Song PhylANOVA pvals 100 trees , # sims per tree = 400
Mean = 0.5584 / StdDev = 0.0237
Frequency
Pval
EPP Syllsong
All converged runs over 100 trees
Low EPP
High EPP
Equal Rates
Frequency
Rate of Syllsong evolution
EPP Syllsong Brownie pvals 100 trees , # sims per tree = 20
Mean = 0.4302 / StdDev = 0.2758
Frequency
Pval
EPP Syllsong PhylANOVA pvals 100 trees , # sims per tree = 400
Mean = 0.0206 / StdDev = 0.0059
Frequency
Pval
EPP Rate All converged runs
Low EPP
High EPP
Equal Rates
Frequency
Rate of Rate evolution
EPP Rate Brownie pvals 8 trees , # sims per tree = 20
Mean = 0.525 / StdDev = 0.2735
Frequency
Pval
EPP Rate PhylANOVA pvals 8 trees , # sims per tree = 400
Mean = 0.7134 / StdDev = 0.0204
Frequency
Pval

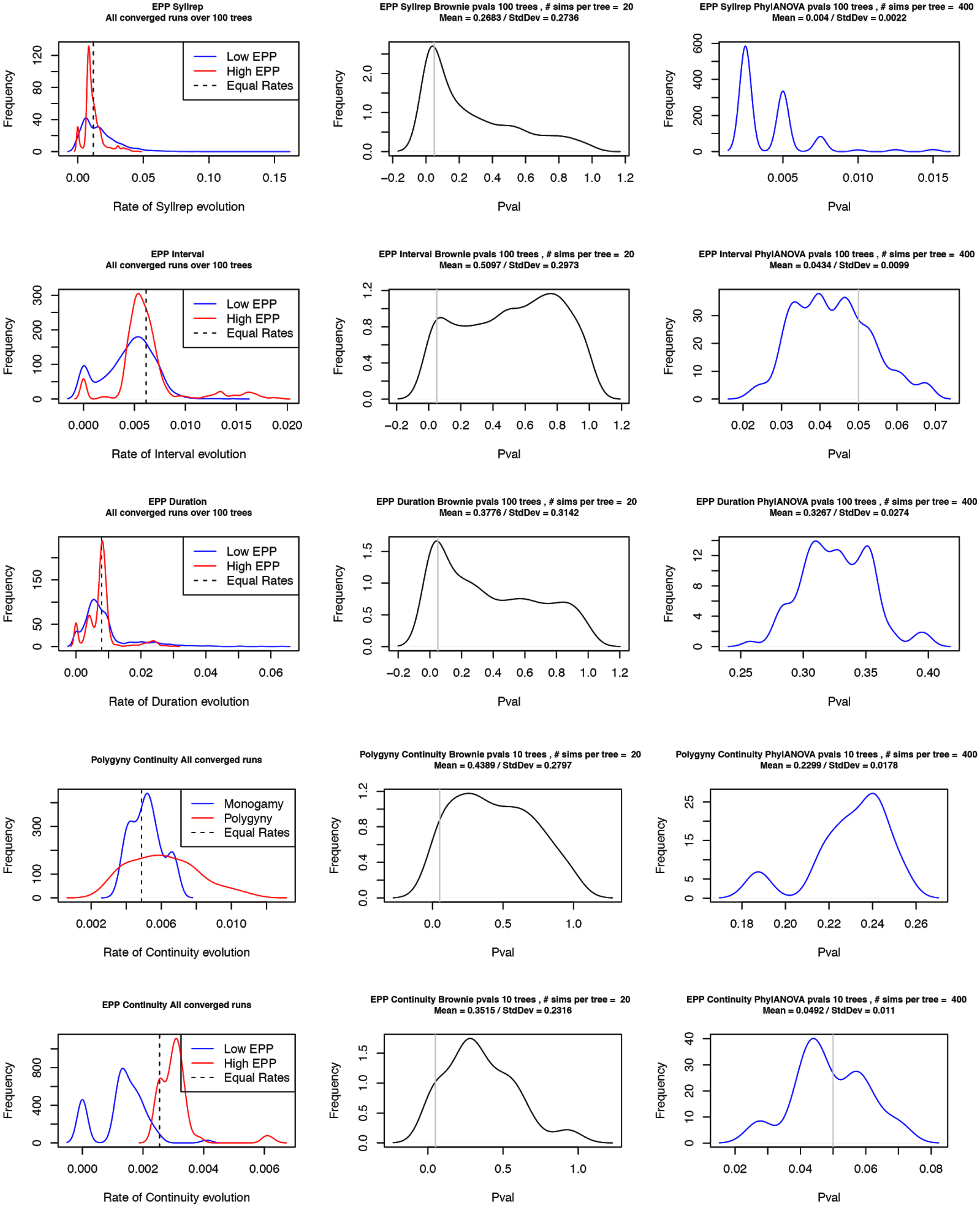
EPP Syllrep
All converged runs over 100 trees
Low EPP
High EPP
Equal Rates
Frequency
Rate of Syllrep evolution
EPP Syllrep Brownie pvals 100 trees , # sims per tree = 20
Mean = 0.2683 / StdDev = 0.2736
Pval
EPP Syllrep PhylANOVA pvals 100 trees , # sims per tree = 400
Mean = 0.004 / StdDev = 0.0022
EPP Interval
All converged runs over 100 trees
Rate of Interval evolution
EPP Interval Brownie pvals 100 trees , # sims per tree = 20
Mean = 0.5097 / StdDev = 0.2973
EPP Interval PhylANOVA pvals 100 trees , # sims per tree = 400
Mean = 0.0434 / StdDev = 0.0099
EPP Duration
All converged runs over 100 trees
Rate of Duration evolution
EPP Duration Brownie pvals 100 trees , # sims per tree = 20
Mean = 0.3776 / StdDev = 0.3142
EPP Duration PhylANOVA pvals 100 trees , # sims per tree = 400
Mean = 0.3267 / StdDev = 0.0274
Polygyny Continuity All converged runs
Monogamy
Polygyny
Equal Rates
Rate of Continuity evolution
Polygyny Continuity Brownie pvals 10 trees , # sims per tree = 20
Mean = 0.4389 / StdDev = 0.2797
Polygyny Continuity PhylANOVA pvals 10 trees , # sims per tree = 400
Mean = 0.2299 / StdDev = 0.0178
EPP Continuity All converged runs
Rate of Continuity evolution
EPP Continuity Brownie pvals 10 trees , # sims per tree = 20
Mean = 0.3515 / StdDev = 0.2316
EPP Continuity PhylANOVA pvals 10 trees , # sims per tree = 400
Mean = 0.0492 / StdDev = 0.011

Supplementary Figure 32 (previous pages): Aggregated results of PhylANOVA and Brownie over 100 trees ("MultiTree/MultiPhy"). 100 trees were sampled from the BirdTree.org Hackett Stage 2 dataset, tree numbers 7001-7100. We performed 20 simulations per tree for Brownie and 400 simulations per tree for PhylANOVA. The left column shows the distribution of rates from all converged runs over all trees. The center column shows the distribution of *p*-values from these Brownie runs. The right column shows the distribution of *p*-values from analysis with PhylANOVA. Polygyny-Continuity, EPP-Rate, and EPP-Continuity all failed to converge on a subset of trees in Brownie, so they were performed on 10 trees for Polygyny-Continuity and EPP-Continuity and 8 trees for EPP-Rate.

Supplementary Figure 33 (following pages): Detecting correlated evolution of mating system and song characteristics over 20 trees ("MultiTree/MultiPhy"). Trees were selected from the BirdTree.org Hackett Stage 2 dataset, tree numbers 7010-7029. We performed 5 simulations per threshold, per tree. The rates shown are the mean rates in the lowest (first column), middle (second column), and highest (third column) thirds of the unique values for each song characteristic over all trees. The fourth column plots the *p*-value from each simulation at each threshold over all trees.

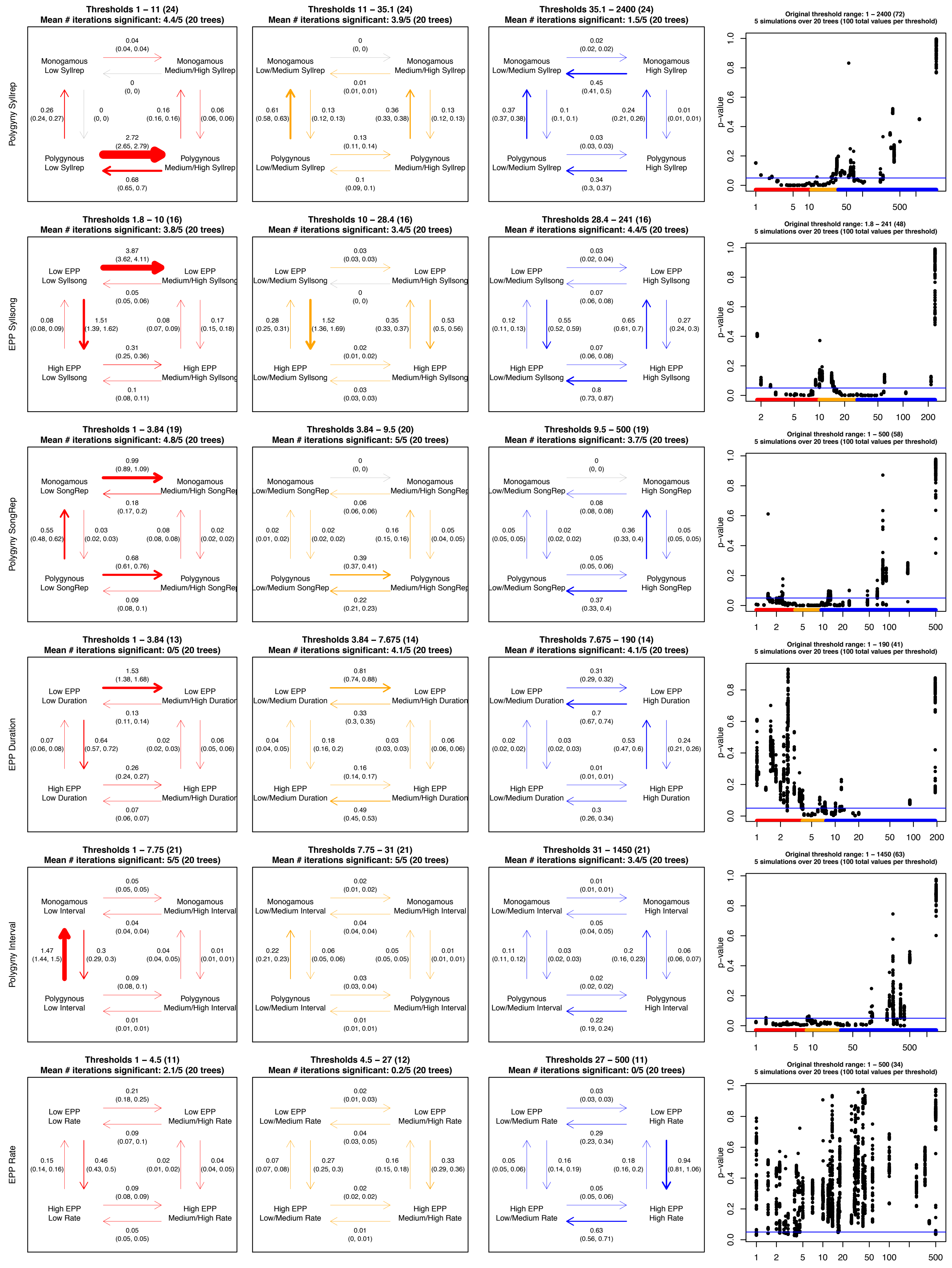
Polygyny Syllrep
Thresholds 1 – 11 (24)
Mean # iterations significant: 4.4/5 (20 trees)
Monogamous Low Syllrep
Monogamous Medium/High Syllrep
Polygynous Low Syllrep
Polygynous Medium/High Syllrep
0.04 (0.04, 0.04)
0 (0, 0)
0.26 (0.24, 0.27)
0 (0, 0)
0.16 (0.16, 0.16)
0.06 (0.06, 0.06)
2.72 (2.65, 2.79)
0.68 (0.65, 0.7)
Thresholds 11 – 35.1 (24)
Mean # iterations significant: 3.9/5 (20 trees)
Monogamous Low/Medium Syllrep
Monogamous Medium/High Syllrep
Polygynous Low/Medium Syllrep
Polygynous Medium/High Syllrep
0 (0, 0)
0.01 (0.01, 0.01)
0.61 (0.58, 0.63)
0.13 (0.12, 0.13)
0.36 (0.33, 0.38)
0.13 (0.12, 0.13)
0.13 (0.11, 0.14)
0.1 (0.09, 0.1)
Thresholds 35.1 – 2400 (24)
Mean # iterations significant: 1.5/5 (20 trees)
Monogamous Low/Medium Syllrep
Monogamous High Syllrep
Polygynous Low/Medium Syllrep
Polygynous High Syllrep
0.02 (0.02, 0.02)
0.45 (0.41, 0.5)
0.37 (0.37, 0.38)
0.1 (0.1, 0.1)
0.24 (0.21, 0.26)
0.01 (0.01, 0.01)
0.03 (0.03, 0.03)
0.34 (0.3, 0.37)
Original threshold range: 1 – 2400 (72)
5 simulations over 20 trees (100 total values per threshold)
p−value
0.0 0.2 0.4 0.6 0.8 1.0
1 5 10 50 500
EPP Syllsong
Thresholds 1.8 – 10 (16)
Mean # iterations significant: 3.8/5 (20 trees)
Low EPP Low Syllsong
Low EPP Medium/High Syllsong
High EPP Low Syllsong
High EPP Medium/High Syllsong
3.87 (3.62, 4.11)
0.05 (0.05, 0.06)
0.08 (0.08, 0.09)
1.51 (1.39, 1.62)
0.08 (0.07, 0.09)
0.17 (0.15, 0.18)
0.31 (0.25, 0.36)
0.1 (0.08, 0.11)
Thresholds 10 – 28.4 (16)
Mean # iterations significant: 3.4/5 (20 trees)
Low EPP Low/Medium Syllsong
Low EPP Medium/High Syllsong
High EPP Low/Medium Syllsong
High EPP Medium/High Syllsong
0.03 (0.03, 0.03)
0 (0, 0)
0.28 (0.25, 0.31)
1.52 (1.36, 1.69)
0.35 (0.33, 0.37)
0.53 (0.5, 0.56)
0.02 (0.01, 0.02)
0.03 (0.03, 0.03)
Thresholds 28.4 – 241 (16)
Mean # iterations significant: 4.4/5 (20 trees)
Low EPP Low/Medium Syllsong
Low EPP High Syllsong
High EPP Low/Medium Syllsong
High EPP High Syllsong
0.03 (0.02, 0.04)
0.07 (0.06, 0.08)
0.12 (0.11, 0.13)
0.55 (0.52, 0.59)
0.65 (0.61, 0.7)
0.27 (0.24, 0.3)
0.07 (0.06, 0.08)
0.8 (0.73, 0.87)
Original threshold range: 1.8 – 241 (48)
5 simulations over 20 trees (100 total values per threshold)
p−value
0.0 0.2 0.4 0.6 0.8 1.0
2 5 10 20 50 100 200
Polygyny SongRep
Thresholds 1 – 3.84 (19)
Mean # iterations significant: 4.8/5 (20 trees)
Monogamous Low SongRep
Monogamous Medium/High SongRep
Polygynous Low SongRep
Polygynous Medium/High SongRep
0.99 (0.89, 1.09)
0.18 (0.17, 0.2)
0.55 (0.48, 0.62)
0.03 (0.02, 0.03)
0.08 (0.08, 0.08)
0.02 (0.02, 0.02)
0.68 (0.61, 0.76)
0.09 (0.08, 0.1)
Thresholds 3.84 – 9.5 (20)
Mean # iterations significant: 5/5 (20 trees)
Monogamous Low/Medium SongRep
Monogamous Medium/High SongRep
Polygynous Low/Medium SongRep
Polygynous Medium/High SongRep
0 (0, 0)
0.06 (0.06, 0.06)
0.02 (0.01, 0.02)
0.02 (0.02, 0.02)
0.16 (0.15, 0.16)
0.05 (0.04, 0.05)
0.39 (0.37, 0.41)
0.22 (0.21, 0.23)
Thresholds 9.5 – 500 (19)
Mean # iterations significant: 3.7/5 (20 trees)
Monogamous Low/Medium SongRep
Monogamous High SongRep
Polygynous Low/Medium SongRep
Polygynous High SongRep
0 (0, 0)
0.08 (0.08, 0.08)
0.05 (0.05, 0.05)
0.02 (0.02, 0.02)
0.36 (0.33, 0.4)
0.05 (0.05, 0.05)
0.05 (0.05, 0.06)
0.37 (0.33, 0.4)
Original threshold range: 1 – 500 (58)
5 simulations over 20 trees (100 total values per threshold)
p−value
0.0 0.2 0.4 0.6 0.8 1.0
1 2 5 10 20 50 100 500
EPP Duration
Thresholds 1 – 3.84 (13)
Mean # iterations significant: 0/5 (20 trees)
Low EPP Low Duration
Low EPP Medium/High Duration
High EPP Low Duration
High EPP Medium/High Duration
1.53 (1.38, 1.68)
0.13 (0.11, 0.14)
0.07 (0.06, 0.08)
0.64 (0.57, 0.72)
0.02 (0.02, 0.03)
0.06 (0.05, 0.06)
0.26 (0.24, 0.27)
0.07 (0.06, 0.07)
Thresholds 3.84 – 7.675 (14)
Mean # iterations significant: 4.1/5 (20 trees)
Low EPP Low/Medium Duration
Low EPP Medium/High Duration
High EPP Low/Medium Duration
High EPP Medium/High Duration
0.81 (0.74, 0.88)
0.33 (0.3, 0.35)
0.04 (0.04, 0.05)
0.18 (0.16, 0.2)
0.03 (0.03, 0.03)
0.06 (0.06, 0.06)
0.16 (0.14, 0.17)
0.49 (0.45, 0.53)
Thresholds 7.675 – 190 (14)
Mean # iterations significant: 4.1/5 (20 trees)
Low EPP Low/Medium Duration
Low EPP High Duration
High EPP Low/Medium Duration
High EPP High Duration
0.31 (0.29, 0.32)
0.7 (0.67, 0.74)
0.02 (0.02, 0.02)
0.03 (0.02, 0.03)
0.53 (0.47, 0.6)
0.24 (0.21, 0.26)
0.01 (0.01, 0.01)
0.3 (0.26, 0.34)
Original threshold range: 1 – 190 (41)
5 simulations over 20 trees (100 total values per threshold)
p−value
0.0 0.2 0.4 0.6 0.8
1 2 5 10 20 50 100 200
Polygyny Interval
Thresholds 1 – 7.75 (21)
Mean # iterations significant: 5/5 (20 trees)
Monogamous Low Interval
Monogamous Medium/High Interval
Polygynous Low Interval
Polygynous Medium/High Interval
0.05 (0.05, 0.05)
0.04 (0.04, 0.04)
1.47 (1.44, 1.5)
0.3 (0.29, 0.3)
0.04 (0.04, 0.05)
0.01 (0.01, 0.01)
0.09 (0.08, 0.1)
0.01 (0.01, 0.01)
Thresholds 7.75 – 31 (21)
Mean # iterations significant: 5/5 (20 trees)
Monogamous Low/Medium Interval
Monogamous Medium/High Interval
Polygynous Low/Medium Interval
Polygynous Medium/High Interval
0.02 (0.01, 0.02)
0.04 (0.04, 0.04)
0.22 (0.21, 0.23)
0.06 (0.05, 0.06)
0.05 (0.05, 0.05)
0.01 (0.01, 0.01)
0.03 (0.03, 0.04)
0.01 (0.01, 0.01)
Thresholds 31 – 1450 (21)
Mean # iterations significant: 3.4/5 (20 trees)
Monogamous Low/Medium Interval
Monogamous High Interval
Polygynous Low/Medium Interval
Polygynous High Interval
0.01 (0.01, 0.01)
0.05 (0.04, 0.05)
0.11 (0.11, 0.12)
0.03 (0.02, 0.03)
0.2 (0.16, 0.23)
0.06 (0.06, 0.07)
0.02 (0.02, 0.02)
0.22 (0.19, 0.24)
Original threshold range: 1 – 1450 (63)
5 simulations over 20 trees (100 total values per threshold)
p−value
0.0 0.2 0.4 0.6 0.8 1.0
1 5 10 50 500
EPP Rate
Thresholds 1 – 4.5 (11)
Mean # iterations significant: 2.1/5 (20 trees)
Low EPP Low Rate
Low EPP Medium/High Rate
High EPP Low Rate
High EPP Medium/High Rate
0.21 (0.18, 0.25)
0.09 (0.07, 0.1)
0.15 (0.14, 0.16)
0.46 (0.43, 0.5)
0.02 (0.01, 0.02)
0.04 (0.04, 0.05)
0.09 (0.08, 0.09)
0.05 (0.05, 0.05)
Thresholds 4.5 – 27 (12)
Mean # iterations significant: 0.2/5 (20 trees)
Low EPP Low/Medium Rate
Low EPP Medium/High Rate
High EPP Low/Medium Rate
High EPP Medium/High Rate
0.02 (0.01, 0.03)
0.04 (0.03, 0.05)
0.07 (0.07, 0.08)
0.27 (0.25, 0.3)
0.16 (0.15, 0.18)
0.33 (0.29, 0.36)
0.02 (0.02, 0.02)
0.01 (0, 0.01)
Thresholds 27 – 500 (11)
Mean # iterations significant: 0/5 (20 trees)
Low EPP Low/Medium Rate
Low EPP High Rate
High EPP Low/Medium Rate
High EPP High Rate
0.03 (0.03, 0.03)
0.29 (0.23, 0.34)
0.05 (0.05, 0.06)
0.16 (0.14, 0.19)
0.18 (0.16, 0.2)
0.94 (0.81, 1.06)
0.05 (0.05, 0.06)
0.63 (0.56, 0.71)
Original threshold range: 1 – 500 (34)
5 simulations over 20 trees (100 total values per threshold)
p−value
0.0 0.2 0.4 0.6 0.8 1.0
1 2 5 10 20 50 100 500

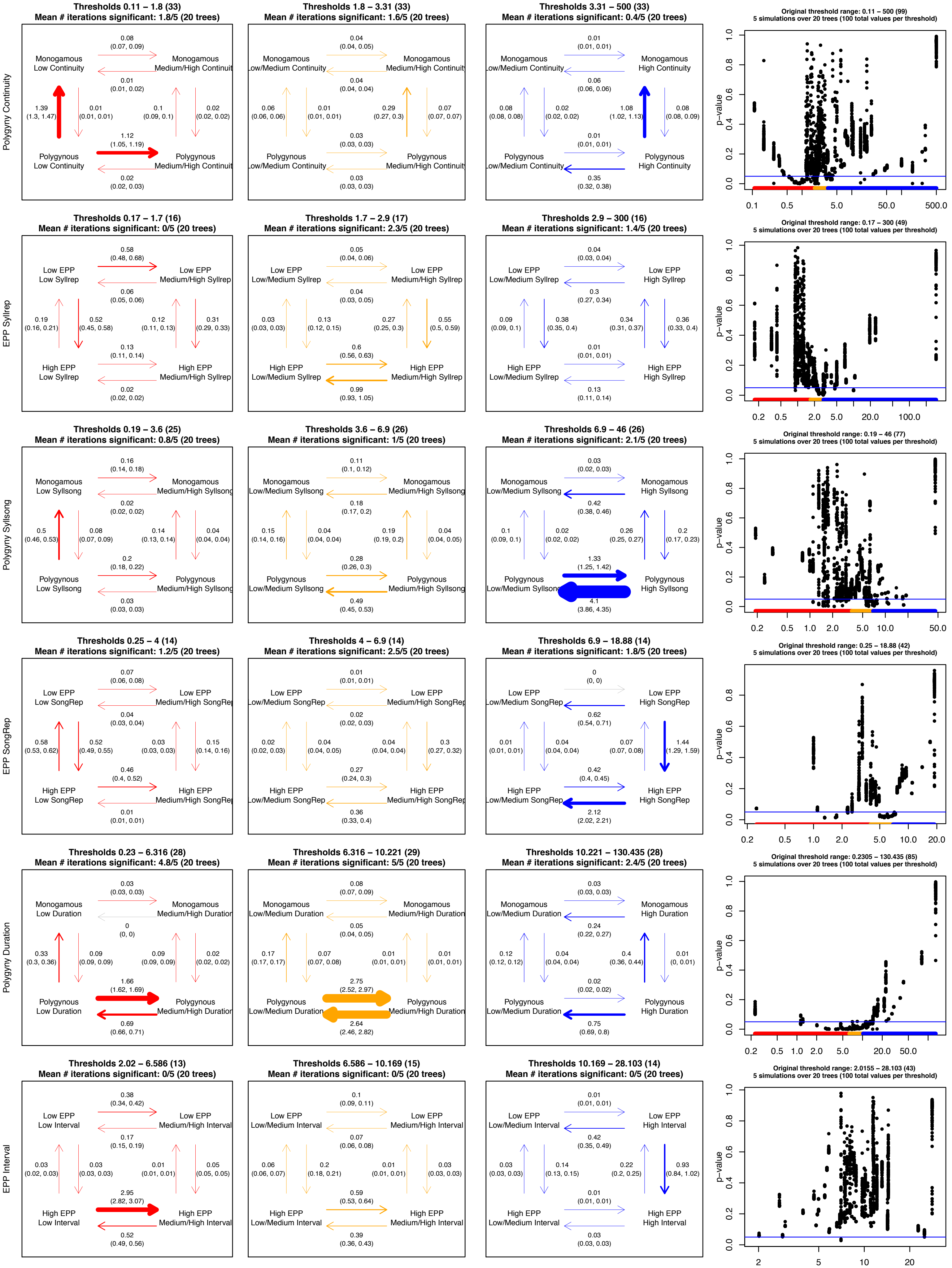

Polygyny Continuity
Thresholds 0.11 – 1.8 (33)
Mean # iterations significant: 1.8/5 (20 trees)
Thresholds 1.8 – 3.31 (33)
Mean # iterations significant: 1.6/5 (20 trees)
Thresholds 3.31 – 500 (33)
Mean # iterations significant: 0.4/5 (20 trees)
Original threshold range: 0.11 – 500 (99)
5 simulations over 20 trees (100 total values per threshold)
EPP Syllrep
Thresholds 0.17 – 1.7 (16)
Mean # iterations significant: 0/5 (20 trees)
Thresholds 1.7 – 2.9 (17)
Mean # iterations significant: 2.3/5 (20 trees)
Thresholds 2.9 – 300 (16)
Mean # iterations significant: 1.4/5 (20 trees)
Original threshold range: 0.17 – 300 (49)
5 simulations over 20 trees (100 total values per threshold)
Polygyny Syllsong
Thresholds 0.19 – 3.6 (25)
Mean # iterations significant: 0.8/5 (20 trees)
Thresholds 3.6 – 6.9 (26)
Mean # iterations significant: 1/5 (20 trees)
Thresholds 6.9 – 46 (26)
Mean # iterations significant: 2.1/5 (20 trees)
Original threshold range: 0.19 – 46 (77)
5 simulations over 20 trees (100 total values per threshold)
EPP SongRep
Thresholds 0.25 – 4 (14)
Mean # iterations significant: 1.2/5 (20 trees)
Thresholds 4 – 6.9 (14)
Mean # iterations significant: 2.5/5 (20 trees)
Thresholds 6.9 – 18.88 (14)
Mean # iterations significant: 1.8/5 (20 trees)
Original threshold range: 0.25 – 18.88 (42)
5 simulations over 20 trees (100 total values per threshold)
Polygyny Duration
Thresholds 0.23 – 6.316 (28)
Mean # iterations significant: 4.8/5 (20 trees)
Thresholds 6.316 – 10.221 (29)
Mean # iterations significant: 5/5 (20 trees)
Thresholds 10.221 – 130.435 (28)
Mean # iterations significant: 2.4/5 (20 trees)
Original threshold range: 0.2305 – 130.435 (85)
5 simulations over 20 trees (100 total values per threshold)
EPP Interval
Thresholds 2.02 – 6.586 (13)
Mean # iterations significant: 0/5 (20 trees)
Thresholds 6.586 – 10.169 (15)
Mean # iterations significant: 0/5 (20 trees)
Thresholds 10.169 – 28.103 (14)
Mean # iterations significant: 0/5 (20 trees)
Original threshold range: 2.0155 – 28.103 (43)
5 simulations over 20 trees (100 total values per threshold)
p−value

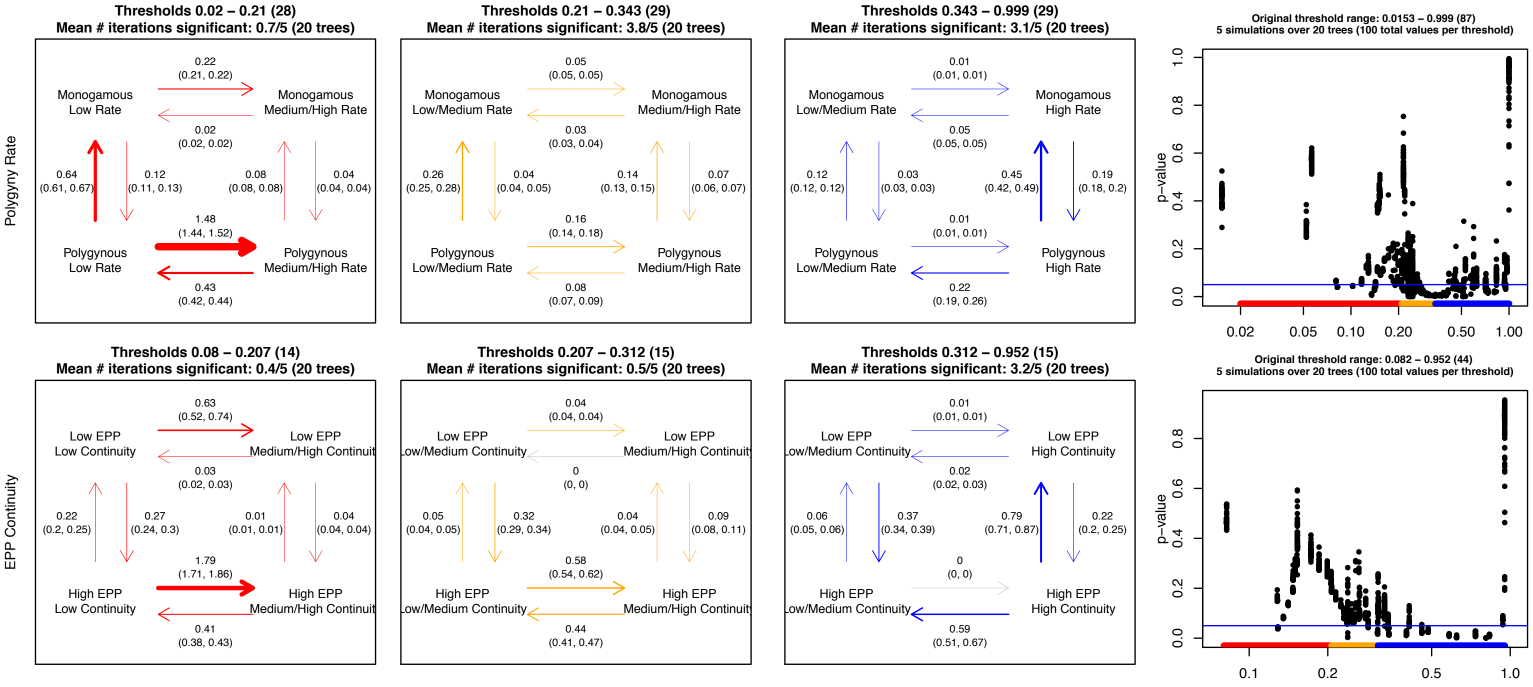
Polygyny Rate
Thresholds 0.02 – 0.21 (28)
Mean # iterations significant: 0.7/5 (20 trees)
Monogamous Low Rate
Monogamous Medium/High Rate
Polygynous Low Rate
Polygynous Medium/High Rate
0.22 (0.21, 0.22)
0.02 (0.02, 0.02)
0.64 (0.61, 0.67)
0.12 (0.11, 0.13)
0.08 (0.08, 0.08)
0.04 (0.04, 0.04)
1.48 (1.44, 1.52)
0.43 (0.42, 0.44)
Thresholds 0.21 – 0.343 (29)
Mean # iterations significant: 3.8/5 (20 trees)
Monogamous Low/Medium Rate
Monogamous Medium/High Rate
Polygynous Low/Medium Rate
Polygynous Medium/High Rate
0.05 (0.05, 0.05)
0.03 (0.03, 0.04)
0.26 (0.25, 0.28)
0.04 (0.04, 0.05)
0.14 (0.13, 0.15)
0.07 (0.06, 0.07)
0.16 (0.14, 0.18)
0.08 (0.07, 0.09)
Thresholds 0.343 – 0.999 (29)
Mean # iterations significant: 3.1/5 (20 trees)
Monogamous Low/Medium Rate
Monogamous High Rate
Polygynous Low/Medium Rate
Polygynous High Rate
0.01 (0.01, 0.01)
0.05 (0.05, 0.05)
0.12 (0.12, 0.12)
0.03 (0.03, 0.03)
0.45 (0.42, 0.49)
0.19 (0.18, 0.2)
0.01 (0.01, 0.01)
0.22 (0.19, 0.26)
Original threshold range: 0.0153 – 0.999 (87)
5 simulations over 20 trees (100 total values per threshold)
p–value
0.0
0.2
0.4
0.6
0.8
1.0
0.02
0.05
0.10
0.20
0.50
1.00
EPP Continuity
Thresholds 0.08 – 0.207 (14)
Mean # iterations significant: 0.4/5 (20 trees)
Low EPP Low Continuity
Low EPP Medium/High Continuit
High EPP Low Continuity
High EPP Medium/High Continuit
0.63 (0.52, 0.74)
0.03 (0.02, 0.03)
0.22 (0.2, 0.25)
0.27 (0.24, 0.3)
0.01 (0.01, 0.01)
0.04 (0.04, 0.04)
1.79 (1.71, 1.86)
0.41 (0.38, 0.43)
Thresholds 0.207 – 0.312 (15)
Mean # iterations significant: 0.5/5 (20 trees)
Low EPP Low/Medium Continuity
Low EPP Medium/High Continuit
High EPP Low/Medium Continuity
High EPP Medium/High Continuit
0.04 (0.04, 0.04)
0 (0, 0)
0.05 (0.04, 0.05)
0.32 (0.29, 0.34)
0.04 (0.04, 0.05)
0.09 (0.08, 0.11)
0.58 (0.54, 0.62)
0.44 (0.41, 0.47)
Thresholds 0.312 – 0.952 (15)
Mean # iterations significant: 3.2/5 (20 trees)
Low EPP Low/Medium Continuity
Low EPP High Continuity
High EPP Low/Medium Continuity
High EPP High Continuity
0.01 (0.01, 0.01)
0.02 (0.02, 0.03)
0.06 (0.05, 0.06)
0.37 (0.34, 0.39)
0.79 (0.71, 0.87)
0.22 (0.2, 0.25)
0 (0, 0)
0.59 (0.51, 0.67)
Original threshold range: 0.082 – 0.952 (44)
5 simulations over 20 trees (100 total values per threshold)
p–value
0.0
0.2
0.4
0.6
0.8
0.1
0.2
0.5
1.0

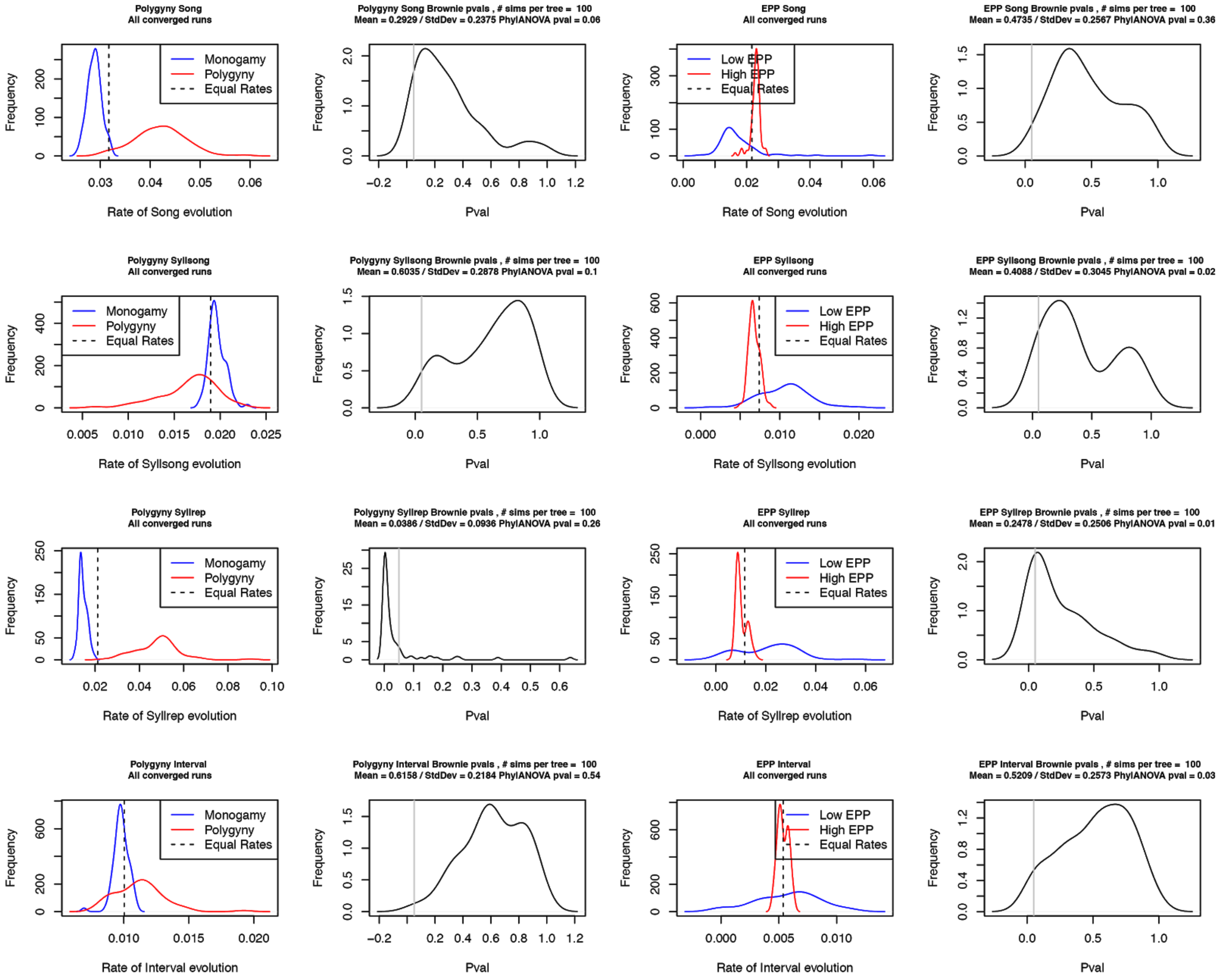
Polygyny Song
All converged runs
Monogamy
Polygyny
Equal Rates
Frequency
Rate of Song evolution
Polygyny Song Brownie pvals , # sims per tree = 100
Mean = 0.2929 / StdDev = 0.2375 PhylANOVA pval = 0.06
Frequency
Pval
EPP Song
All converged runs
Low EPP
High EPP
Equal Rates
Frequency
Rate of Song evolution
EPP Song Brownie pvals , # sims per tree = 100
Mean = 0.4735 / StdDev = 0.2567 PhylANOVA pval = 0.36
Frequency
Pval
Polygyny Syllsong
All converged runs
Monogamy
Polygyny
Equal Rates
Frequency
Rate of Syllsong evolution
Polygyny Syllsong Brownie pvals , # sims per tree = 100
Mean = 0.6035 / StdDev = 0.2878 PhylANOVA pval = 0.1
Frequency
Pval
EPP Syllsong
All converged runs
Low EPP
High EPP
Equal Rates
Frequency
Rate of Syllsong evolution
EPP Syllsong Brownie pvals , # sims per tree = 100
Mean = 0.4088 / StdDev = 0.3045 PhylANOVA pval = 0.02
Frequency
Pval
Polygyny Syllrep
All converged runs
Monogamy
Polygyny
Equal Rates
Frequency
Rate of Syllrep evolution
Polygyny Syllrep Brownie pvals , # sims per tree = 100
Mean = 0.0386 / StdDev = 0.0936 PhylANOVA pval = 0.26
Frequency
Pval
EPP Syllrep
All converged runs
Low EPP
High EPP
Equal Rates
Frequency
Rate of Syllrep evolution
EPP Syllrep Brownie pvals , # sims per tree = 100
Mean = 0.2478 / StdDev = 0.2506 PhylANOVA pval = 0.01
Frequency
Pval
Polygyny Interval
All converged runs
Monogamy
Polygyny
Equal Rates
Frequency
Rate of Interval evolution
Polygyny Interval Brownie pvals , # sims per tree = 100
Mean = 0.6158 / StdDev = 0.2184 PhylANOVA pval = 0.54
Frequency
Pval
EPP Interval
All converged runs
Low EPP
High EPP
Equal Rates
Frequency
Rate of Interval evolution
EPP Interval Brownie pvals , # sims per tree = 100
Mean = 0.5209 / StdDev = 0.2573 PhylANOVA pval = 0.03
Frequency
Pval

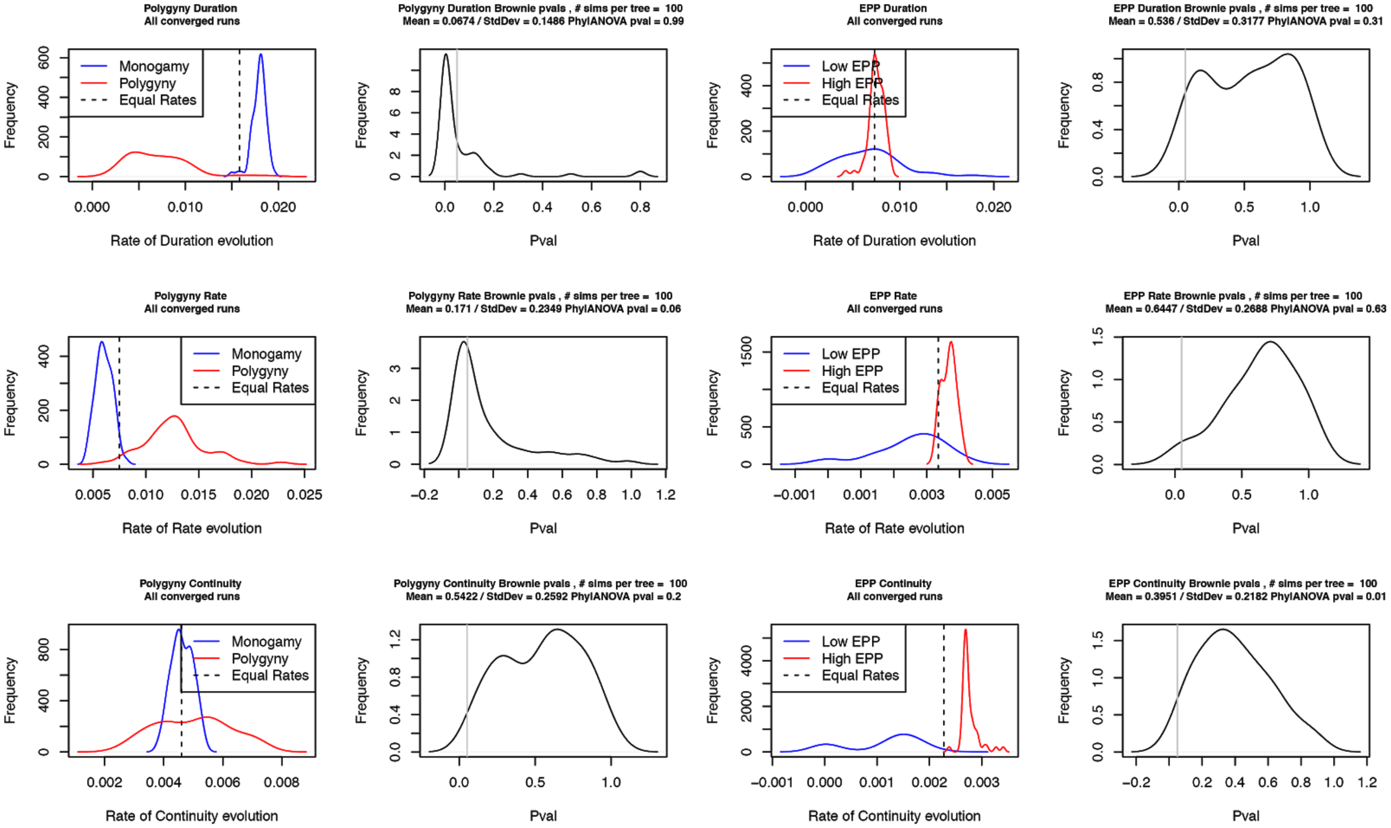

Supplementary Figure 34: Results of Brownie and PhylANOVA analyses performed using the consensus tree generated from Hackett trees using only species with genetic data ("GeneTree"). 1000 trees were sampled from the BirdTree.org Hackett Stage 1 dataset, tree numbers 7001-8000. We performed 100 simulations for brownie and 2000 simulations for PhylANOVA. The first and third columns show the distribution of rates from all converged simulations. The second and fourth columns show the distribution of *p*-values from these Brownie runs.

Supplementary Figure 35 (following pages): Results of BayesTraits analyses performed using the consensus tree generated from Hackett trees using only species with genetic data ("GeneTree"). 1000 trees were sampled from the BirdTree.org Hackett Stage 1 dataset, tree numbers 7001-8000. We performed 20 simulations per threshold. The rates shown are the mean rates in the lowest (first column), middle (second column), and highest (third column) thirds of the unique values for each song characteristic. The fourth column plots the *p*-value from each simulation at each threshold.

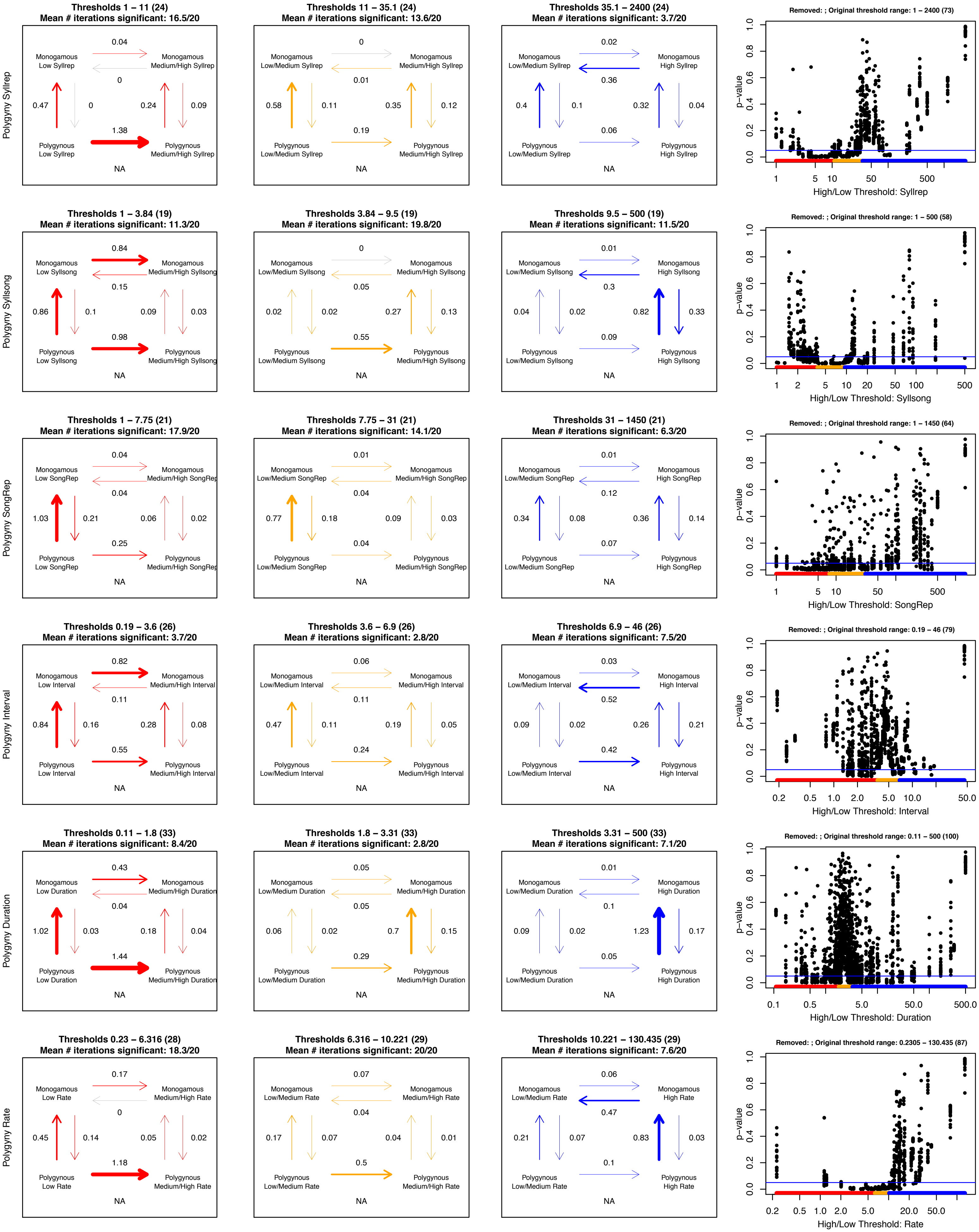
Thresholds 1 – 11 (24)
Mean # iterations significant: 16.5/20
Thresholds 11 – 35.1 (24)
Mean # iterations significant: 13.6/20
Thresholds 35.1 – 2400 (24)
Mean # iterations significant: 3.7/20
Removed: ; Original threshold range: 1 – 2400 (73)
High/Low Threshold: Syllrep
Polygyny Syllrep
Thresholds 1 – 3.84 (19)
Mean # iterations significant: 11.3/20
Thresholds 3.84 – 9.5 (19)
Mean # iterations significant: 19.8/20
Thresholds 9.5 – 500 (19)
Mean # iterations significant: 11.5/20
Removed: ; Original threshold range: 1 – 500 (58)
High/Low Threshold: Syllsong
Polygyny Syllsong
Thresholds 1 – 7.75 (21)
Mean # iterations significant: 17.9/20
Thresholds 7.75 – 31 (21)
Mean # iterations significant: 14.1/20
Thresholds 31 – 1450 (21)
Mean # iterations significant: 6.3/20
Removed: ; Original threshold range: 1 – 1450 (64)
High/Low Threshold: SongRep
Polygyny SongRep
Thresholds 0.19 – 3.6 (26)
Mean # iterations significant: 3.7/20
Thresholds 3.6 – 6.9 (26)
Mean # iterations significant: 2.8/20
Thresholds 6.9 – 46 (26)
Mean # iterations significant: 7.5/20
Removed: ; Original threshold range: 0.19 – 46 (79)
High/Low Threshold: Interval
Polygyny Interval
Thresholds 0.11 – 1.8 (33)
Mean # iterations significant: 8.4/20
Thresholds 1.8 – 3.31 (33)
Mean # iterations significant: 2.8/20
Thresholds 3.31 – 500 (33)
Mean # iterations significant: 7.1/20
Removed: ; Original threshold range: 0.11 – 500 (100)
High/Low Threshold: Duration
Polygyny Duration
Thresholds 0.23 – 6.316 (28)
Mean # iterations significant: 18.3/20
Thresholds 6.316 – 10.221 (29)
Mean # iterations significant: 20/20
Thresholds 10.221 – 130.435 (29)
Mean # iterations significant: 7.6/20
Removed: ; Original threshold range: 0.2305 – 130.435 (87)
High/Low Threshold: Rate
Polygyny Rate
p–value

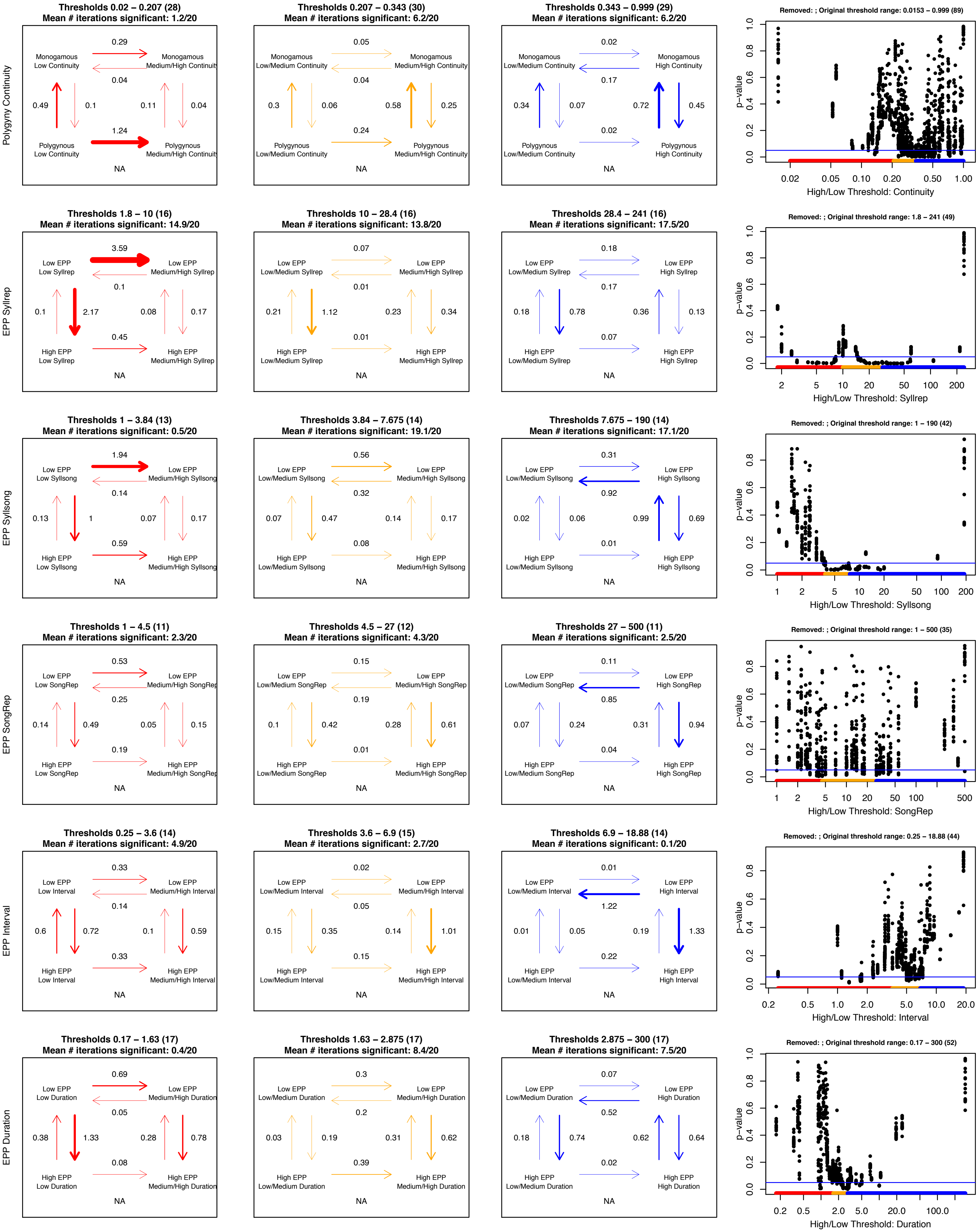

Polygyny Continuity
Thresholds 0.02 – 0.207 (28)
Mean # iterations significant: 1.2/20
Monogamous Low Continuity
Monogamous Medium/High Continuit
Polygynous Low Continuity
Polygynous Medium/High Continuit
0.29
0.04
0.49
0.1
0.11
0.04
1.24
NA
Thresholds 0.207 – 0.343 (30)
Mean # iterations significant: 6.2/20
Monogamous Low/Medium Continuity
Monogamous Medium/High Continuity
Polygynous Low/Medium Continuity
Polygynous Medium/High Continuity
0.05
0.04
0.3
0.06
0.58
0.25
0.24
NA
Thresholds 0.343 – 0.999 (29)
Mean # iterations significant: 6.2/20
Monogamous Low/Medium Continuity
Monogamous High Continuity
Polygynous Low/Medium Continuity
Polygynous High Continuity
0.02
0.17
0.34
0.07
0.72
0.45
0.02
NA
Removed: ; Original threshold range: 0.0153 – 0.999 (89)
p–value
0.0
0.2
0.4
0.6
0.8
1.0
0.02
0.05
0.10
0.20
0.50
1.00
High/Low Threshold: Continuity
EPP Syllrep
Thresholds 1.8 – 10 (16)
Mean # iterations significant: 14.9/20
Low EPP Low Syllrep
Low EPP Medium/High Syllrep
High EPP Low Syllrep
High EPP Medium/High Syllrep
3.59
0.1
0.1
2.17
0.08
0.17
0.45
NA
Thresholds 10 – 28.4 (16)
Mean # iterations significant: 13.8/20
Low EPP Low/Medium Syllrep
Low EPP Medium/High Syllrep
High EPP Low/Medium Syllrep
High EPP Medium/High Syllrep
0.07
0.01
0.21
1.12
0.23
0.34
0.01
NA
Thresholds 28.4 – 241 (16)
Mean # iterations significant: 17.5/20
Low EPP Low/Medium Syllrep
Low EPP High Syllrep
High EPP Low/Medium Syllrep
High EPP High Syllrep
0.18
0.17
0.18
0.78
0.36
0.13
0.07
NA
Removed: ; Original threshold range: 1.8 – 241 (49)
p–value
0.0
0.2
0.4
0.6
0.8
1.0
2
5
10
20
50
100
200
High/Low Threshold: Syllrep
EPP Syllsong
Thresholds 1 – 3.84 (13)
Mean # iterations significant: 0.5/20
Low EPP Low Syllsong
Low EPP Medium/High Syllsong
High EPP Low Syllsong
High EPP Medium/High Syllsong
1.94
0.14
0.13
1
0.07
0.17
0.59
NA
Thresholds 3.84 – 7.675 (14)
Mean # iterations significant: 19.1/20
Low EPP Low/Medium Syllsong
Low EPP Medium/High Syllsong
High EPP Low/Medium Syllsong
High EPP Medium/High Syllsong
0.56
0.32
0.07
0.47
0.14
0.17
0.08
NA
Thresholds 7.675 – 190 (14)
Mean # iterations significant: 17.1/20
Low EPP Low/Medium Syllsong
Low EPP High Syllsong
High EPP Low/Medium Syllsong
High EPP High Syllsong
0.31
0.92
0.02
0.06
0.99
0.69
0.01
NA
Removed: ; Original threshold range: 1 – 190 (42)
p–value
0.0
0.2
0.4
0.6
0.8
1
2
5
10
20
50
100
200
High/Low Threshold: Syllsong
EPP SongRep
Thresholds 1 – 4.5 (11)
Mean # iterations significant: 2.3/20
Low EPP Low SongRep
Low EPP Medium/High SongRep
High EPP Low SongRep
High EPP Medium/High SongRep
0.53
0.25
0.14
0.49
0.05
0.15
0.19
NA
Thresholds 4.5 – 27 (12)
Mean # iterations significant: 4.3/20
Low EPP Low/Medium SongRep
Low EPP Medium/High SongRep
High EPP Low/Medium SongRep
High EPP Medium/High SongRep
0.15
0.19
0.1
0.42
0.28
0.61
0.01
NA
Thresholds 27 – 500 (11)
Mean # iterations significant: 2.5/20
Low EPP Low/Medium SongRep
Low EPP High SongRep
High EPP Low/Medium SongRep
High EPP High SongRep
0.11
0.85
0.07
0.24
0.31
0.94
0.04
NA
Removed: ; Original threshold range: 1 – 500 (35)
p–value
0.0
0.2
0.4
0.6
0.8
1
2
5
10
20
50
100
500
High/Low Threshold: SongRep
EPP Interval
Thresholds 0.25 – 3.6 (14)
Mean # iterations significant: 4.9/20
Low EPP Low Interval
Low EPP Medium/High Interval
High EPP Low Interval
High EPP Medium/High Interval
0.33
0.14
0.6
0.72
0.1
0.59
0.33
NA
Thresholds 3.6 – 6.9 (15)
Mean # iterations significant: 2.7/20
Low EPP Low/Medium Interval
Low EPP Medium/High Interval
High EPP Low/Medium Interval
High EPP Medium/High Interval
0.02
0.05
0.15
0.35
0.14
1.01
0.15
NA
Thresholds 6.9 – 18.88 (14)
Mean # iterations significant: 0.1/20
Low EPP Low/Medium Interval
Low EPP High Interval
High EPP Low/Medium Interval
High EPP High Interval
0.01
1.22
0.01
0.05
0.19
1.33
0.22
NA
Removed: ; Original threshold range: 0.25 – 18.88 (44)
p–value
0.0
0.2
0.4
0.6
0.8
0.2
0.5
1.0
2.0
5.0
10.0
20.0
High/Low Threshold: Interval
EPP Duration
Thresholds 0.17 – 1.63 (17)
Mean # iterations significant: 0.4/20
Low EPP Low Duration
Low EPP Medium/High Duration
High EPP Low Duration
High EPP Medium/High Duration
0.69
0.05
0.38
1.33
0.28
0.78
0.08
NA
Thresholds 1.63 – 2.875 (17)
Mean # iterations significant: 8.4/20
Low EPP Low/Medium Duration
Low EPP Medium/High Duration
High EPP Low/Medium Duration
High EPP Medium/High Duration
0.3
0.2
0.03
0.19
0.31
0.62
0.39
NA
Thresholds 2.875 – 300 (17)
Mean # iterations significant: 7.5/20
Low EPP Low/Medium Duration
Low EPP High Duration
High EPP Low/Medium Duration
High EPP High Duration
0.07
0.52
0.18
0.74
0.62
0.64
0.02
NA
Removed: ; Original threshold range: 0.17 – 300 (52)
p–value
0.0
0.2
0.4
0.6
0.8
1.0
0.2
0.5
2.0
5.0
20.0
100.0
High/Low Threshold: Duration

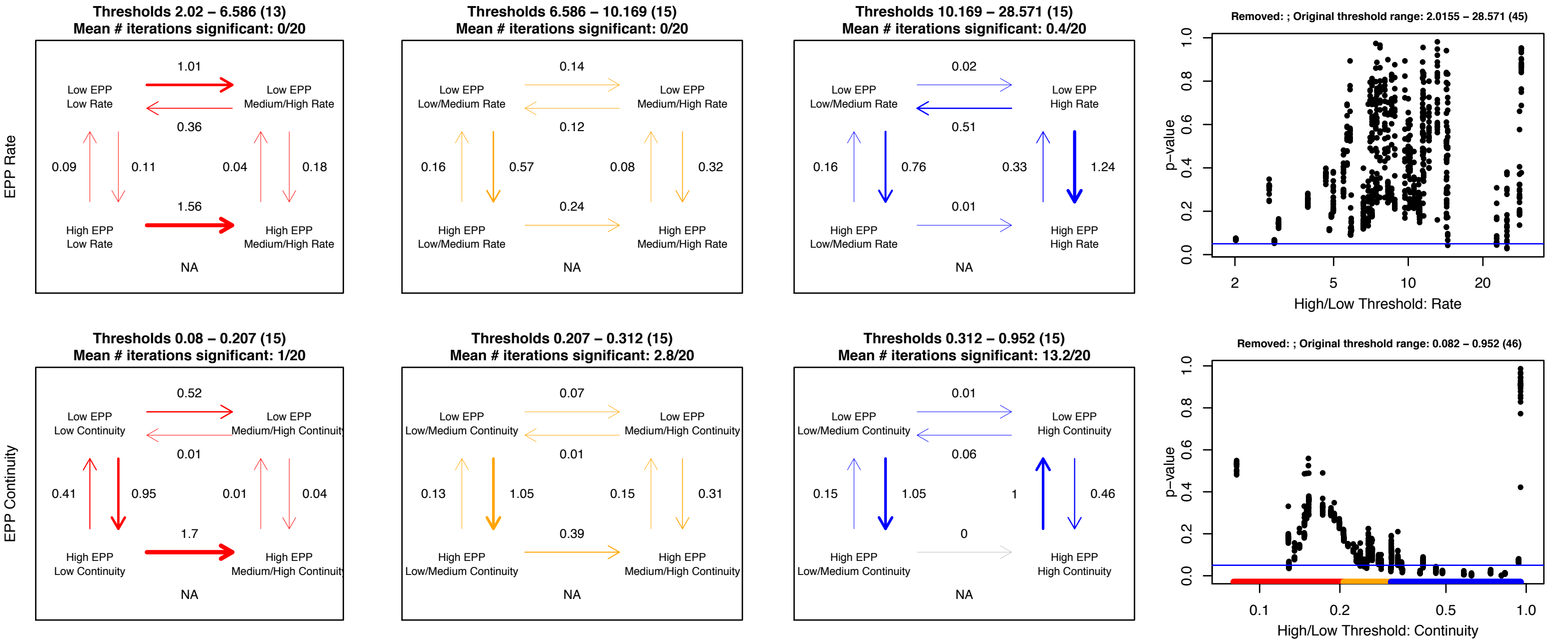
EPP Rate
Thresholds 2.02 – 6.586 (13)
Mean # iterations significant: 0/20
Low EPP
Low Rate
Low EPP
Medium/High Rate
High EPP
Low Rate
High EPP
Medium/High Rate
1.01
0.36
0.09
0.11
0.04
0.18
1.56
NA
Thresholds 6.586 – 10.169 (15)
Mean # iterations significant: 0/20
Low EPP
Low/Medium Rate
Low EPP
Medium/High Rate
High EPP
Low/Medium Rate
High EPP
Medium/High Rate
0.14
0.12
0.16
0.57
0.08
0.32
0.24
NA
Thresholds 10.169 – 28.571 (15)
Mean # iterations significant: 0.4/20
Low EPP
Low/Medium Rate
Low EPP
High Rate
High EPP
Low/Medium Rate
High EPP
High Rate
0.02
0.51
0.16
0.76
0.33
1.24
0.01
NA
Removed: ; Original threshold range: 2.0155 – 28.571 (45)
p−value
High/Low Threshold: Rate
EPP Continuity
Thresholds 0.08 – 0.207 (15)
Mean # iterations significant: 1/20
Low EPP
Low Continuity
Low EPP
Medium/High Continuity
High EPP
Low Continuity
High EPP
Medium/High Continuity
0.52
0.01
0.41
0.95
0.01
0.04
1.7
NA
Thresholds 0.207 – 0.312 (15)
Mean # iterations significant: 2.8/20
Low EPP
Low/Medium Continuity
Low EPP
Medium/High Continuity
High EPP
Low/Medium Continuity
High EPP
Medium/High Continuity
0.07
0.01
0.13
1.05
0.15
0.31
0.39
NA
Thresholds 0.312 – 0.952 (15)
Mean # iterations significant: 13.2/20
Low EPP
Low/Medium Continuity
Low EPP
High Continuity
High EPP
Low/Medium Continuity
High EPP
High Continuity
0.01
0.06
0.15
1.05
1
0.46
0
NA
Removed: ; Original threshold range: 0.082 – 0.952 (46)
p−value
High/Low Threshold: Continuity

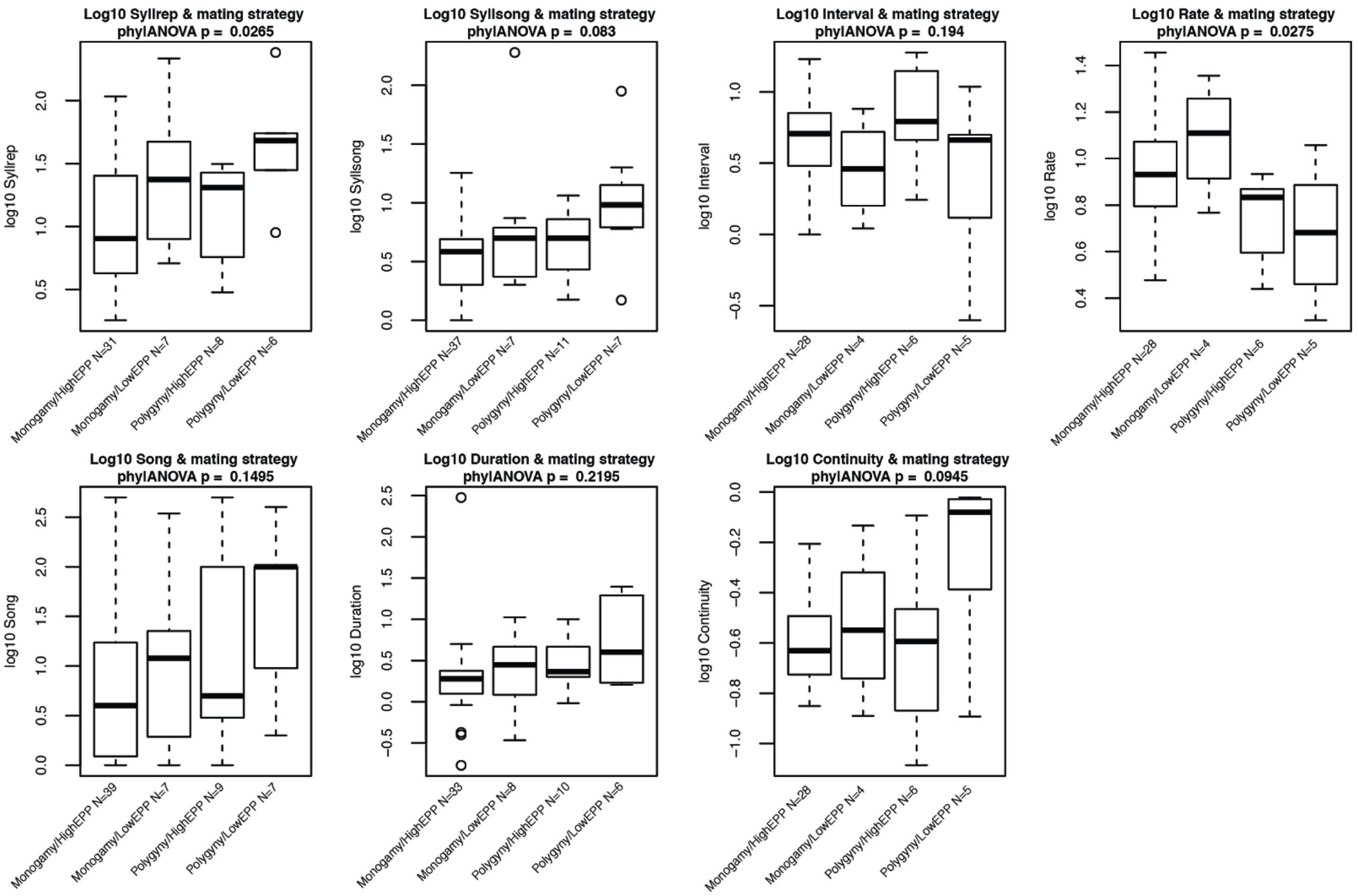

Supplementary Figure 36: Distributions of song characteristics by overall mating strategy. We separated species into four groups formed by combining EPP classification and mating systems classification (Monogamy-LowEPP, Monogamy-HighEPP, Polygyny-LowEPP, Polygyny-HighEPP). The number of species with song characteristic data falling into each group are noted on the x-axis labels for each song characteristic plot. Box plots indicate the median (black bar) interquartile range (IQR, box) and Q1−1.5*IQR, Q3+1.5*IQR (whiskers) of each distribution.

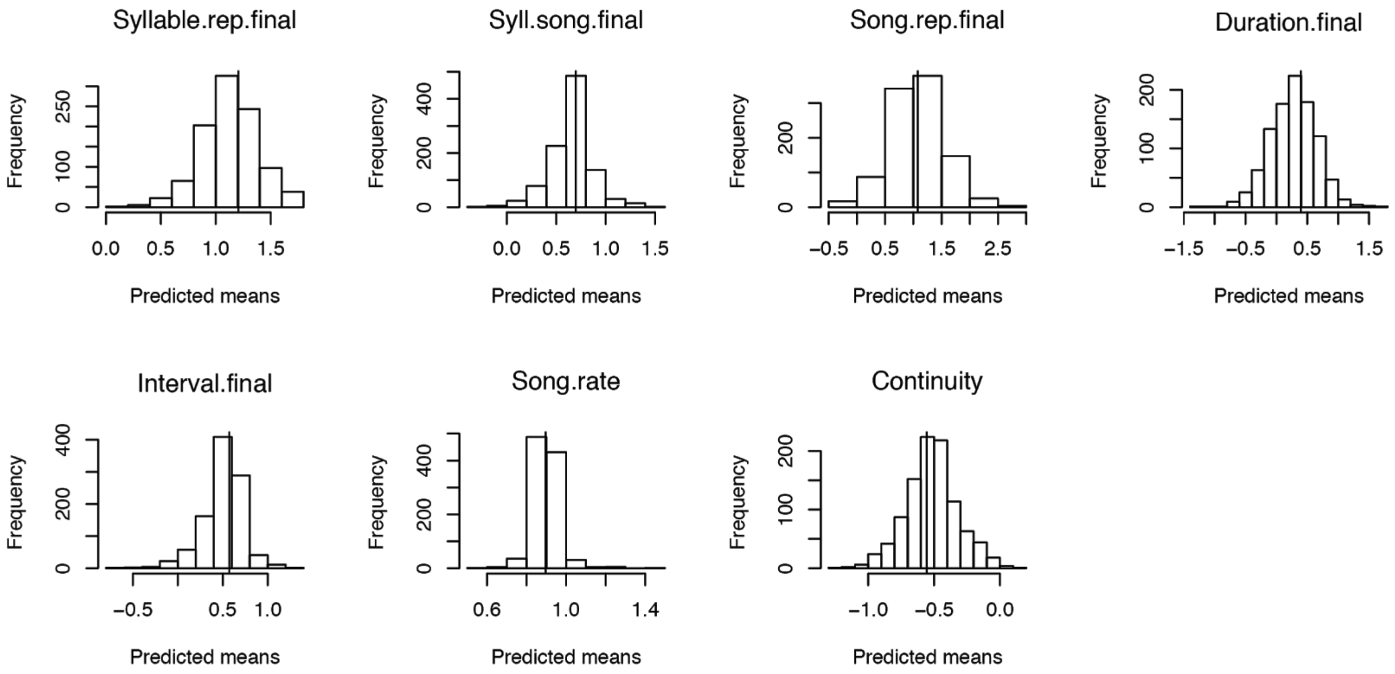

Supplementary Figure 37: Posterior predictive check of Generalized Linear Mixed Model (GLMM) showing distribution of predicted means. We performed a GLMM for each song characteristic, accounting for phylogenetic relationships, mating system, EPP, and the interaction between mating system and EPP. All distributions are nearly centered on the actual mean of the data, indicated by the vertical line. This indicates support for the model accurately predicting the data.

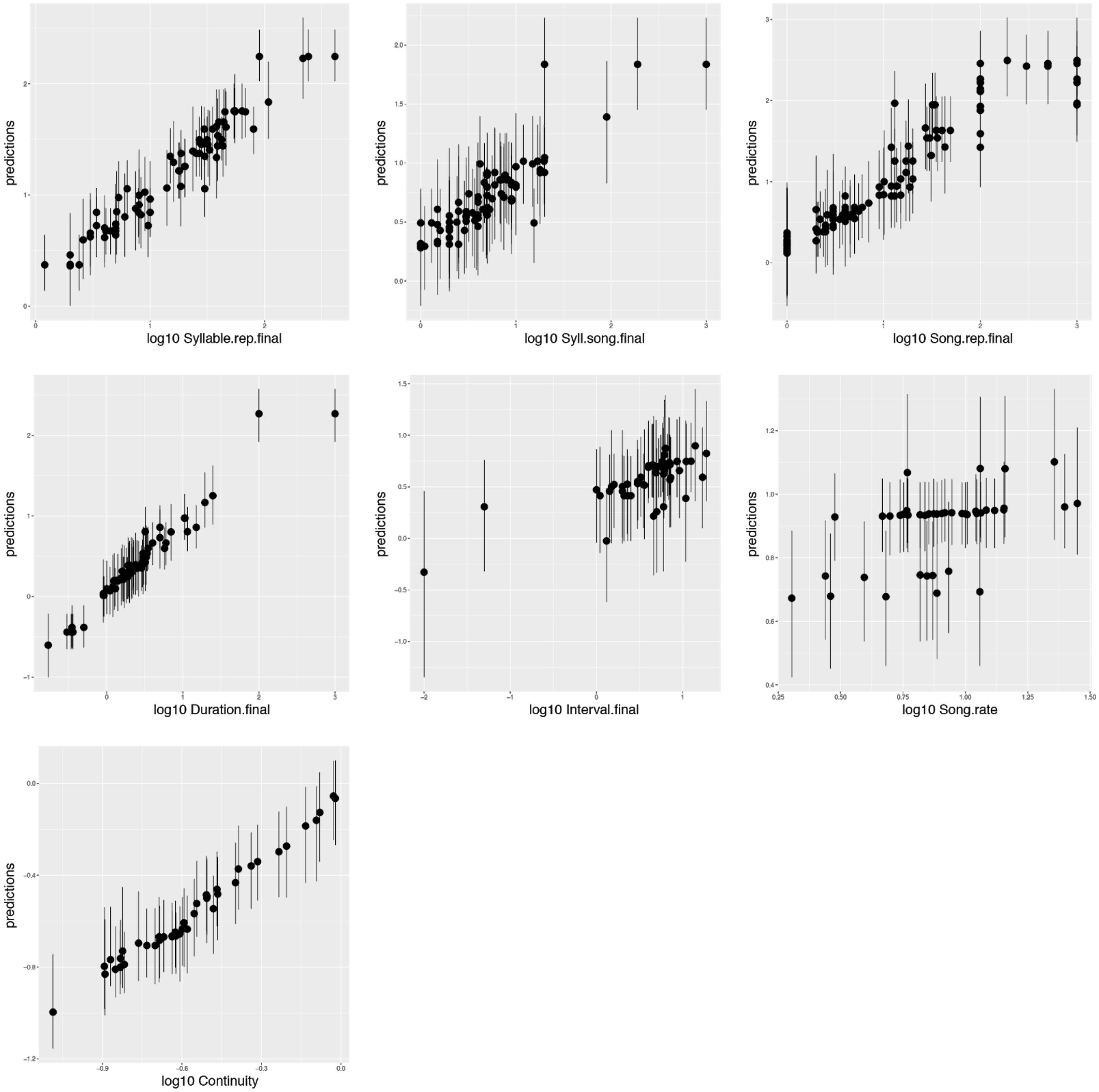

Supplementary Figure 38: Posterior predictive check of the GLMM that accounts for phylogeny, mating system, EPP, and the interaction between mating system and EPP. Plots are of the species values predicted by the model versus the actual species values for each song characteristic. Error bars represent 95% confidence intervals for these predictions. The correlation for syllable repertoire and most other song characteristics is positive and close to linear, indicating that the model, when accounting for phylogeny, mating system, EPP, and repeated measures, accurately represents the data.

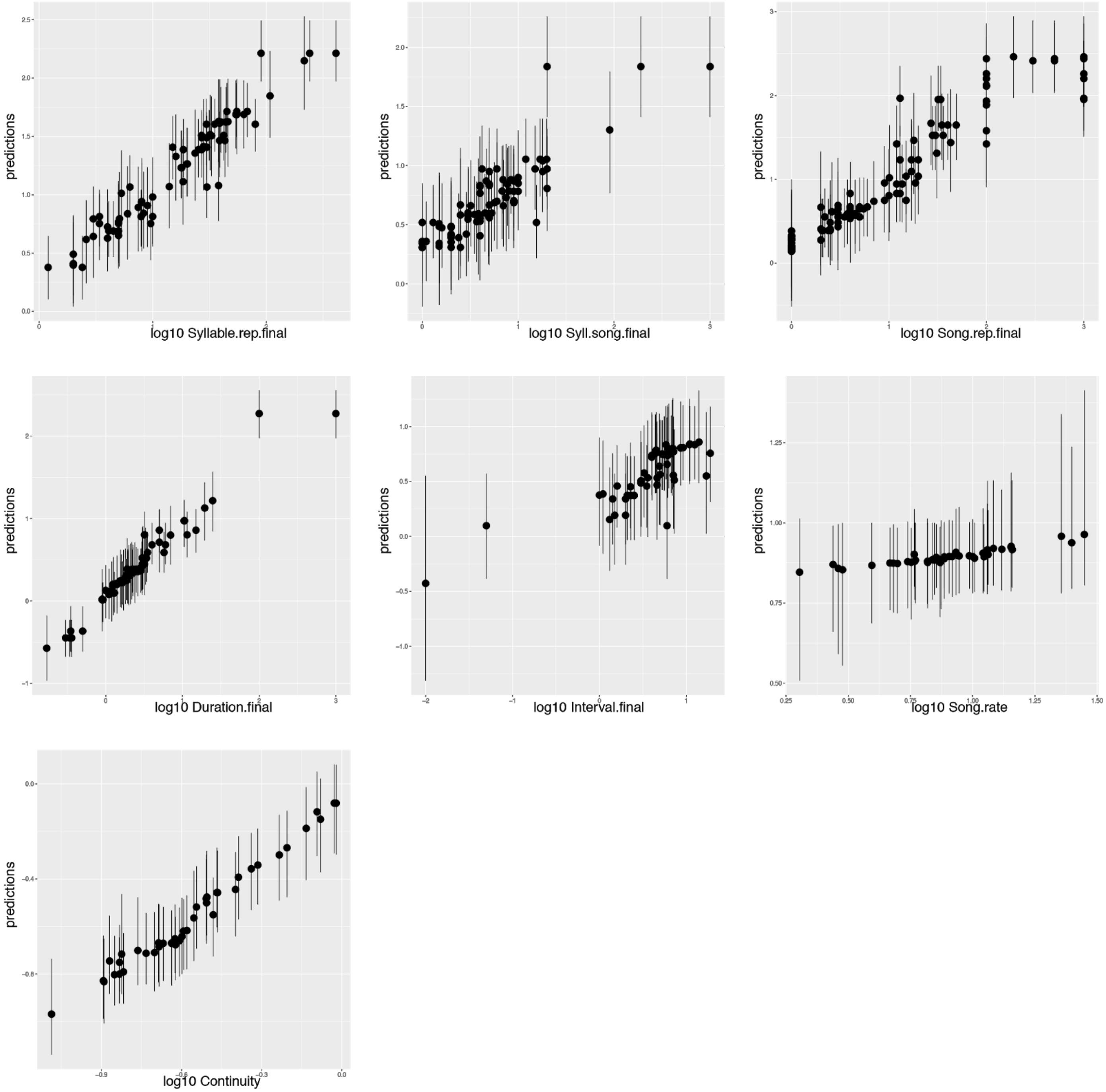

Supplementary Figure 39: Posterior predictive check of GLMM that accounts only for phylogeny. Plots are of the species values predicted by the model versus the actual species values for each song characteristic. Error bars represent 95% confidence intervals for these predictions. The correlation for most song characteristics is positive and approximately linear, indicating that the model accurately represents the data when accounting just for the phylogenetic relationships between species.

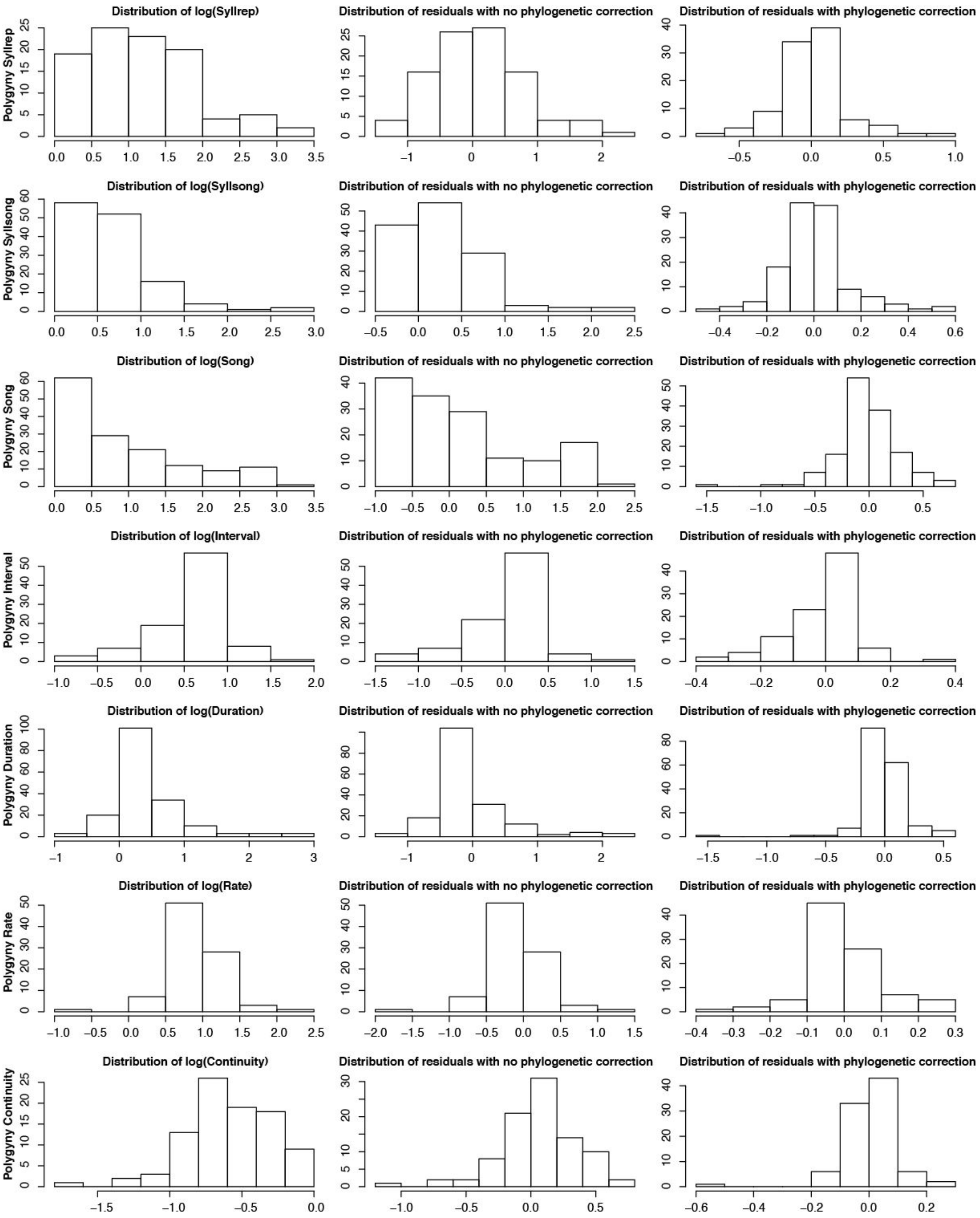

Supplementary Figure 40: Visualizing the distributions of data and residuals in song and mating system comparisons. Left panels show the distribution of each song characteristic (log_{10} transformed). Center panels show the residuals of an ANOVA comparing song characteristics between monogamous and

polygynous species with no phylogenetic correction. Right panels show the residuals of the corresponding ANOVA with phylogenetic correction (right). Note that axes are not uniform between panels.

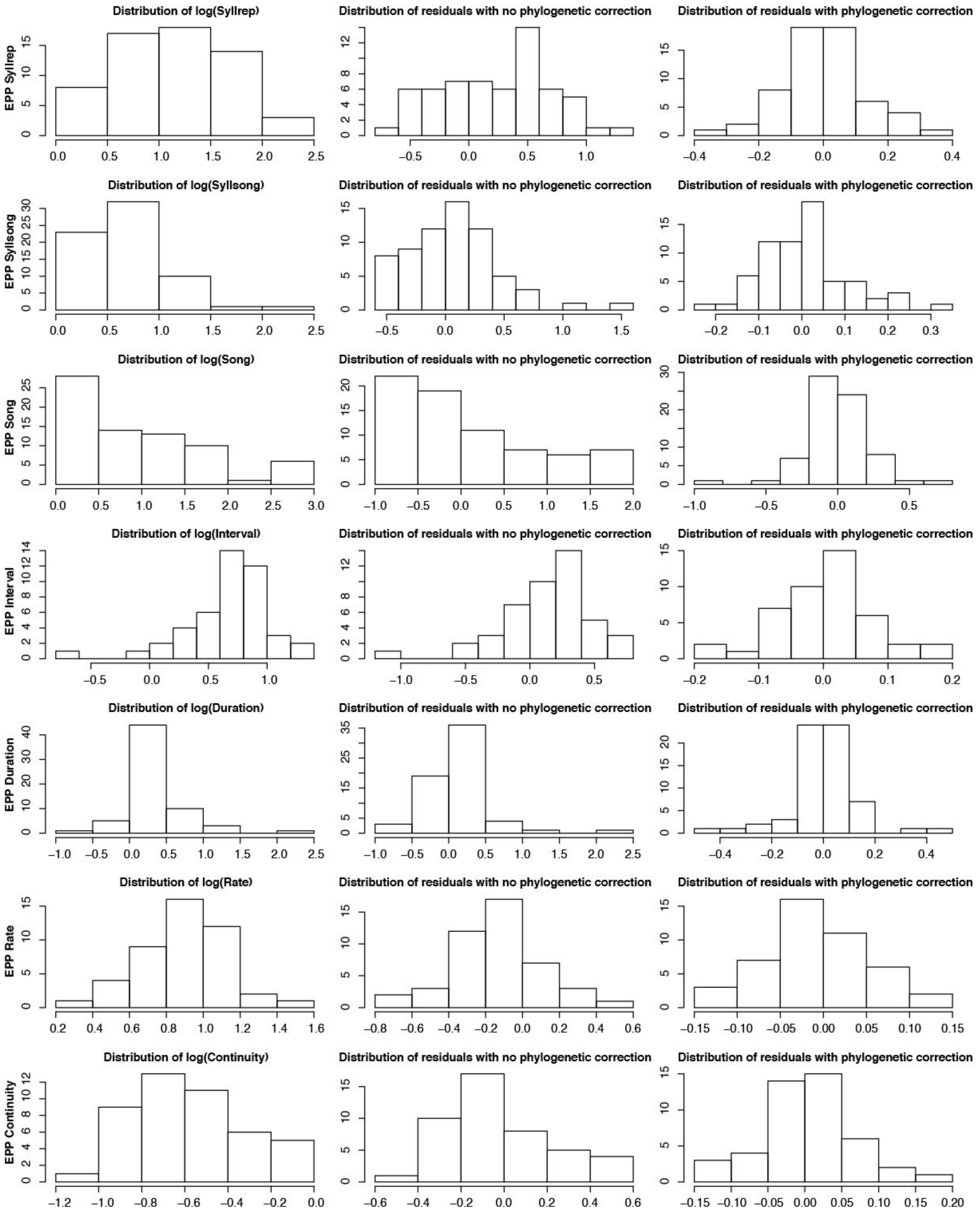

Supplementary Figure 41: Visualizing the distributions of data and residuals in song and EPP comparisons. Left panels show the distribution of each song characteristic ($\log_{10}$ transformed). Center panels show the residuals of an ANOVA comparing song characteristics between Low-EPP and High-EPP species

with no phylogenetic correction. Right panels show the residuals of the corresponding ANOVA with phylogenetic correction (right). Note that axes are not uniform between panels.

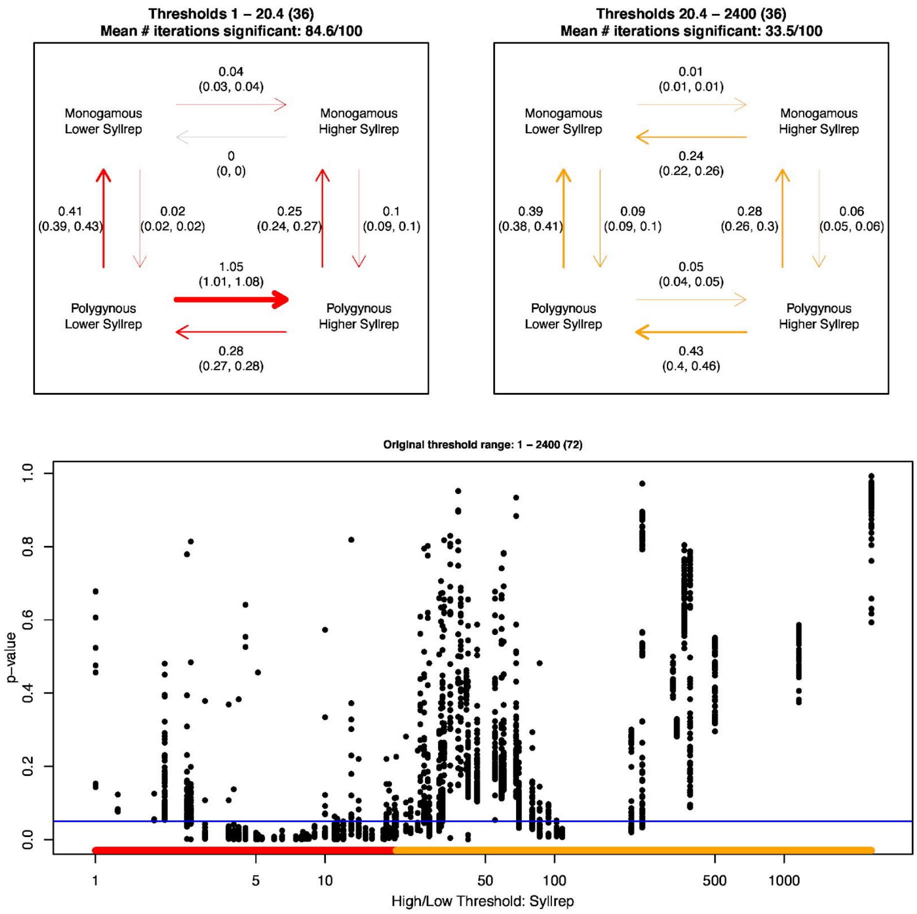

Supplementary Figure 42: Alternative two-bin visualization of tests of correlated evolution between mating system and syllable repertoire. We tested the correlated evolution of syllable repertoire and mating system using BayesTraits, with syllable repertoire made binary based on a threshold delineating smaller versus larger syllable repertoire values. Each observed value of syllable repertoire was used as the threshold for 100 runs of BayesTraits. We generated transition plots by calculating the mean rate and 95% confidence interval (in parentheses) for each transition rate, shown here when the threshold between low and high syllable repertoire is within a given fraction of the range of unique observed values of syllable repertoire. The upper left panel shows the lower half of values (red arrows), while the upper right panel shows the upper half of values (yellow arrows). The lower panel shows the likelihood-ratio test *p*-value for each run of BayesTraits at each threshold value. The color bar along the x-axis denotes the range of threshold values that are included in each segment, corresponding to arrow color.

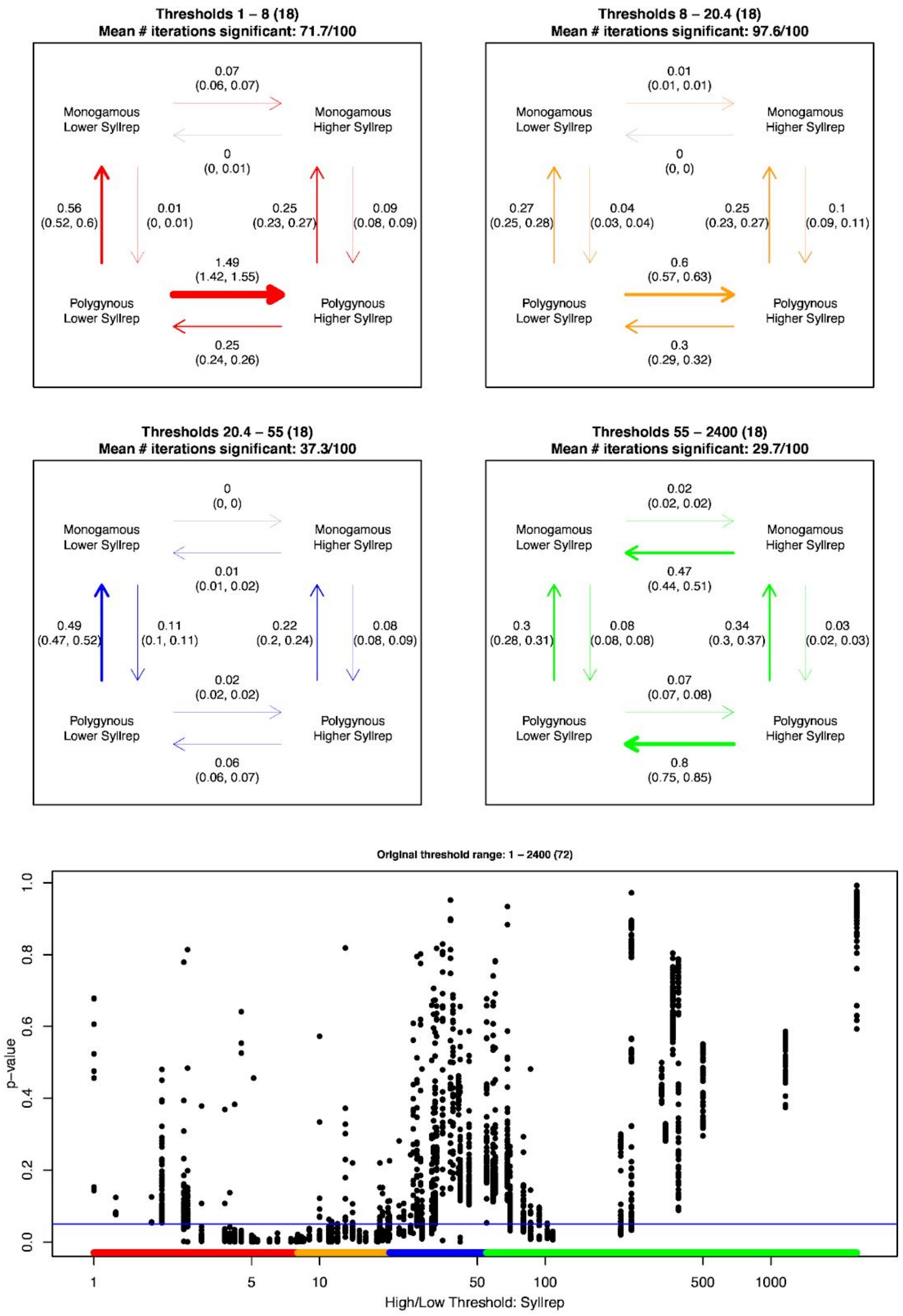

Supplementary Figure 43: Alternative four-bin visualization of tests of correlated evolution between mating system and syllable repertoire. We tested the correlated evolution of syllable repertoire and mating system using BayesTraits, with syllable repertoire made binary based on a threshold delineating smaller versus larger syllable repertoire values. Each observed value of syllable repertoire was used as the threshold for 100 runs of BayesTraits. We generated transition plots by calculating the mean rate and 95% confidence interval (in parentheses) for each transition rate, shown here when the threshold between low and high syllable repertoire is within a given fraction of the range of unique observed values of syllable repertoire. The upper left panel shows the lowest quarter of values (red arrows), the upper right panel shows the second quarter of values (yellow arrows), the middle left panel shows the third quarter of values (blue arrows), and the middle right panel shows the highest quarter of values (green arrows). The lower panel shows the likelihood-ratio test *p*-value for each run of BayesTraits at each threshold value. The color bar along the x-axis denotes the range of threshold values that are included in each segment, corresponding to arrow color.

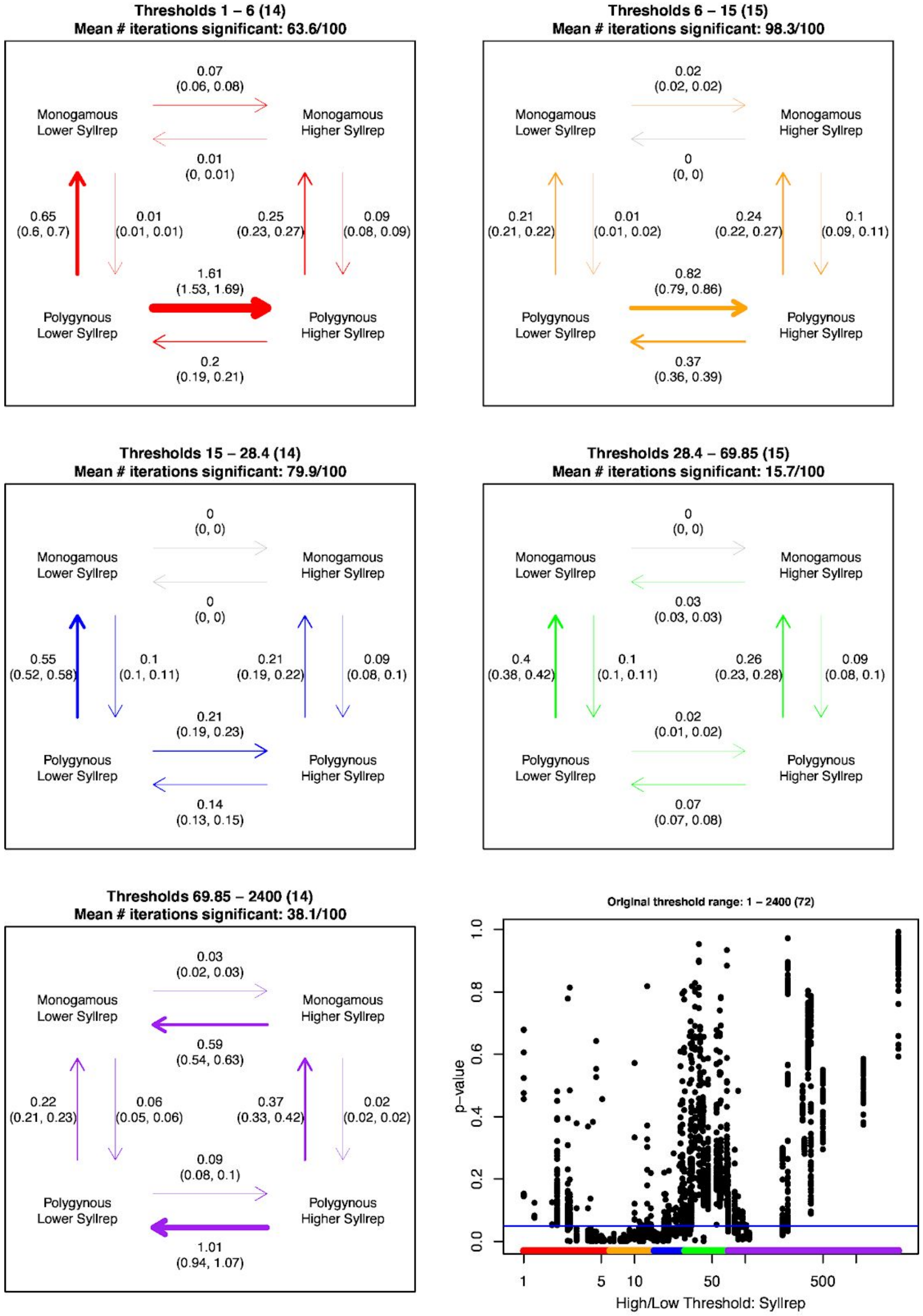

Supplementary Figure 44: Alternative five-bin visualization of tests of correlated evolution between mating system and syllable repertoire. We tested the correlated evolution of syllable repertoire and mating system using BayesTraits, with syllable repertoire made binary based on a threshold delineating smaller versus larger syllable repertoire values. Each observed value of syllable repertoire was used as the threshold for 100 runs of BayesTraits. We generated transition plots by calculating the mean rate and 95% confidence interval (in parentheses) for each transition rate, shown here when the threshold between low and high syllable repertoire is within a given fraction of the range of unique observed values of syllable repertoire. Starting from the top right corner, each fifth of the threshold values is shown from lowest to highest: red arrows, yellow arrows, blue arrows, green arrows, and purple arrows. The lower right panel shows the likelihood-ratio test *p*-value for each run of BayesTraits at each threshold value. The color bar along the x-axis denotes the range of threshold values that are included in each segment, corresponding to arrow color.

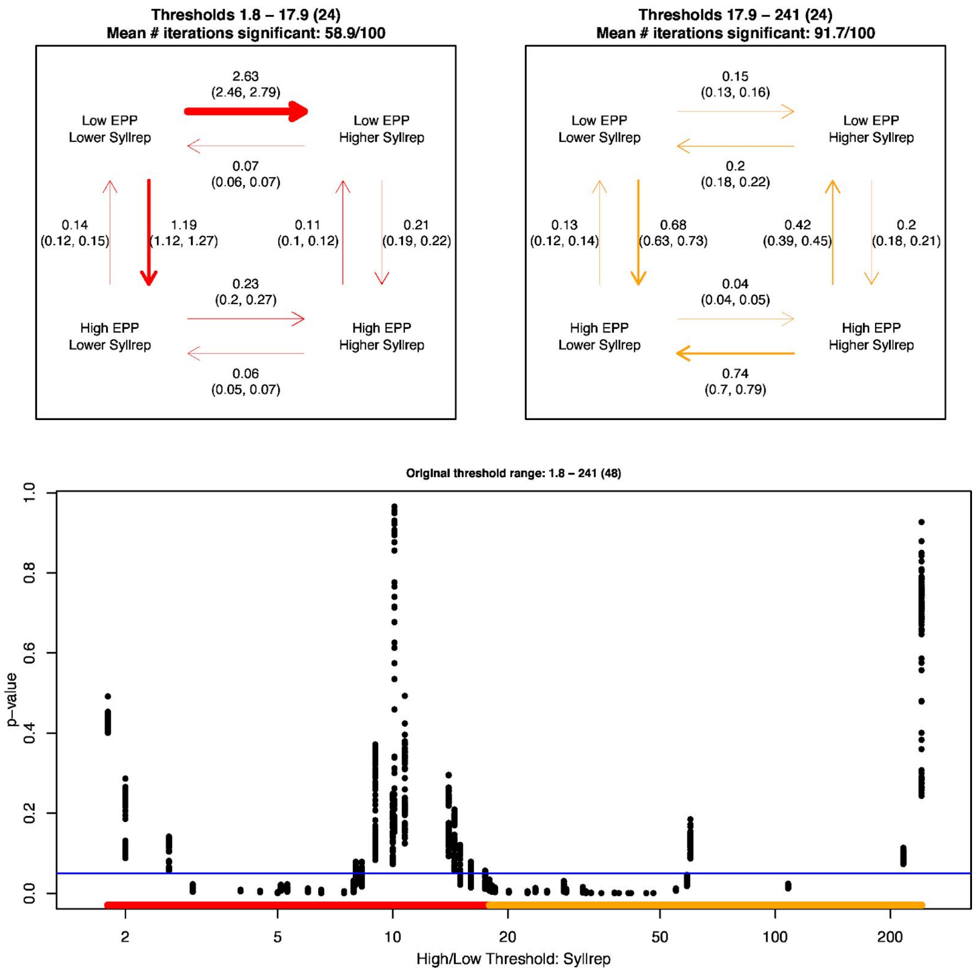

Supplementary Figure 45: Alternative two-bin visualization of tests of correlated evolution between EPP and syllable repertoire. We tested the correlated evolution of syllable repertoire and EPP using BayesTraits, with syllable repertoire made binary based on a threshold delineating smaller versus larger syllable repertoire values. Each observed value of syllable repertoire was used as the threshold for 100 runs of BayesTraits. We generated transition plots by calculating the mean rate and 95% confidence interval (in parentheses) for each transition rate, shown here when the threshold between low and high syllable repertoire is within a given fraction of the range of unique observed values of syllable repertoire. The upper left panel shows the lower half of values (red arrows), while the upper right panel shows the upper half of values (yellow arrows). The lower panel shows the likelihood-ratio test *p*-value for each run of BayesTraits at each threshold value. The color bar along the x-axis denotes the range of threshold values that are included in each segment, corresponding to arrow color.

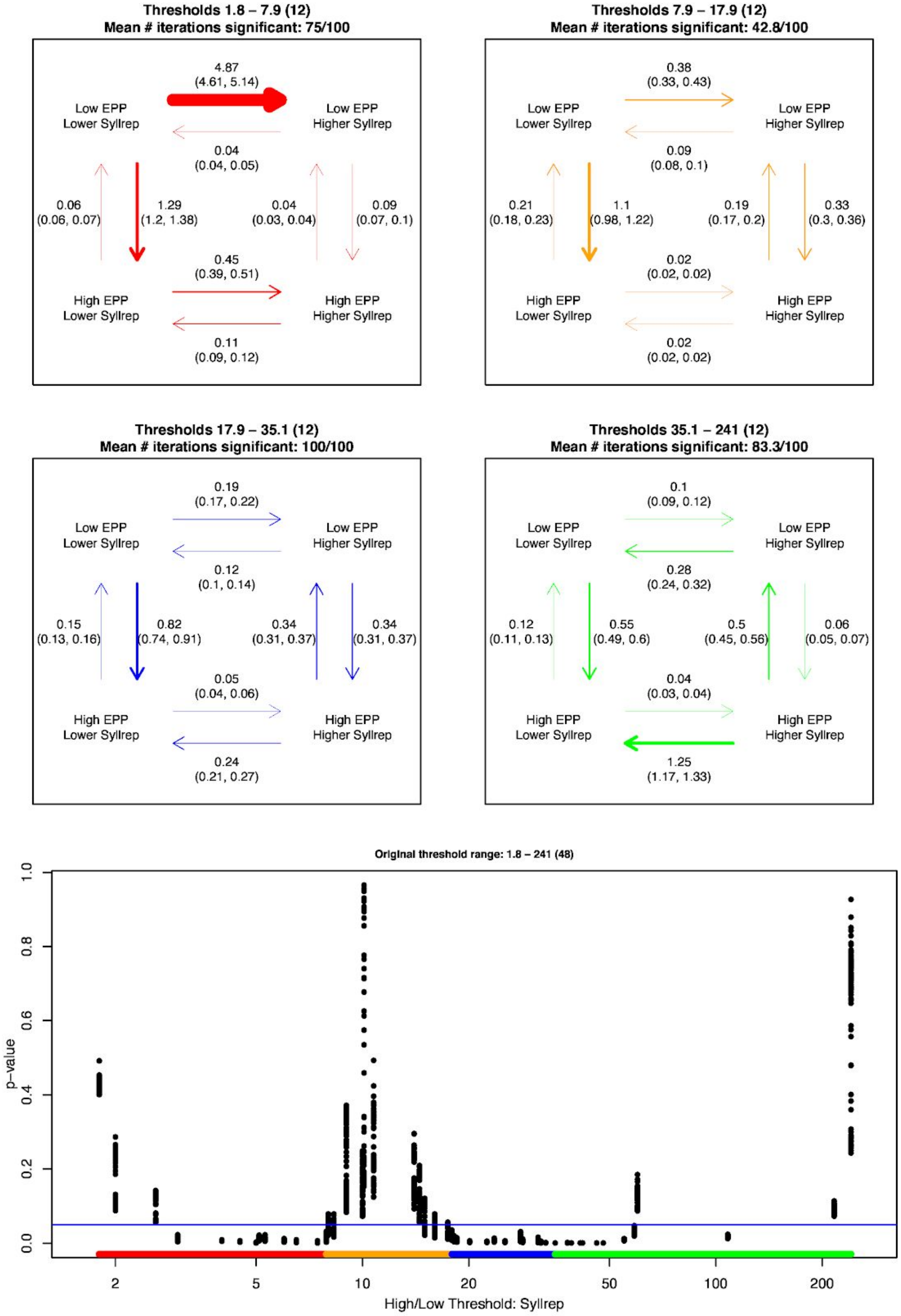

Supplementary Figure 46: Alternative four-bin visualization of tests of correlated evolution between EPP and syllable repertoire. We tested the correlated evolution of syllable repertoire and EPP using BayesTraits, with syllable repertoire made binary based on a threshold delineating smaller versus larger syllable repertoire values. Each observed value of syllable repertoire was used as the threshold for 100 runs of BayesTraits. We generated transition plots by calculating the mean rate and 95% confidence interval (in parentheses) for each transition rate, shown here when the threshold between low and high syllable repertoire is within a given fraction of the range of unique observed values of syllable repertoire. The upper left panel shows the lowest quarter of values (red arrows), the upper right panel shows the second quarter of values (yellow arrows), the middle left panel shows the third quarter of values (blue arrows), and the middle right panel shows the highest quarter of values (green arrows). The lower panel shows the likelihood-ratio test *p*-value for each run of BayesTraits at each threshold value. The color bar along the x-axis denotes the range of threshold values that are included in each segment, corresponding to arrow color.

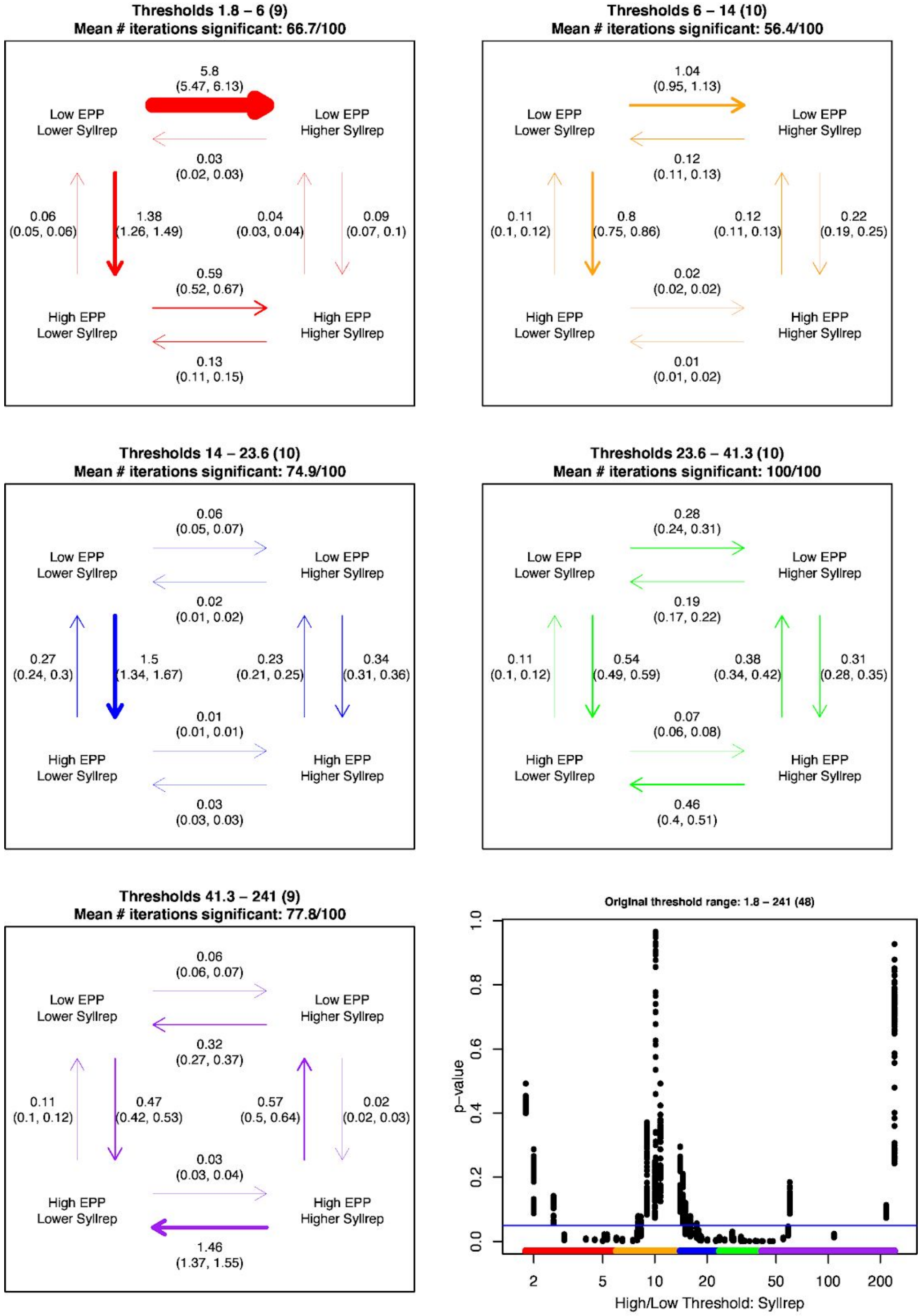

Supplementary Figure 47: Alternative five-bin visualization of tests of correlated evolution between EPP and syllable repertoire. We tested the correlated evolution of syllable repertoire and EPP using BayesTraits, with syllable repertoire made binary based on a threshold delineating smaller versus larger syllable repertoire values. Each observed value of syllable repertoire was used as the threshold for 100 runs of BayesTraits. We generated transition plots by calculating the mean rate and 95% confidence interval (in parentheses) for each transition rate, shown here when the threshold between low and high syllable repertoire is within a given fraction of the range of unique observed values of syllable repertoire. Starting from the top right corner, each fifth of the threshold values is shown from lowest to highest: red arrows, yellow arrows, blue arrows, green arrows, and purple arrows. The lower right panel shows the likelihood-ratio test *p*-value for each run of BayesTraits at each threshold value. The color bar along the x-axis denotes the range of threshold values that are included in each segment, corresponding to arrow color.

Supplementary Tables

**Song Trait**	**N**	**κ**	***p*-value**	**λ**	**λ LogL**	**λ LogL0**	***p*-value**
Syllable rep. size	122	0.4574	1.50E-04	0.8359	-104.43	-126.82	2.19E-11
Syllables per song	171	0.2544	0.02983	0.5725	-102.49	-114.64	8.23E-07
Song rep. size	217	0.5071	1.00E-05	0.9999	-204.32	-272.38	1.89E-31
Intersong interval	127	0.3985	5.00E-05	0.8598	-46.23	-65.29	6.69E-10
Song duration	228	0.3794	1.33E-03	0.9524	-147.31	-188.41	1.23E-19
Song rate	122	0.3162	8.60E-04	0.3399	-38.69	-45.70	1.80E-04
Song continuity	122	0.4018	1.00E-05	0.8176	4.29	-17.11	6.06E-11

Supplementary Table 1. Measuring phylogenetic signal with Blomberg's κ and Pagel's λ. We assessed the significance of Blomberg's κ with a resampling test (100,000 permutations) and the significance of Pagel's λ. with a likelihood-ratio test. Values that were significant after a Holm-Bonferroni correction for multiple hypothesis testing are highlighted in green.

		Wilcoxon rank-sum			phylANOVA			Brownie		
**Mating variable**	**Song characteristic**	***p*-value**	**Rank**	**$\alpha_{corrected}$**	***p*-value**	**Rank**	**$\alpha_{corrected}$**	***p*-value**	**Rank**	**$\alpha_{corrected}$**
Mating system	Syllable rep. size	0.176	5	0.0167	0.25	5	0.0167	0.0056	1	0.0071
Mating system	Syllables per song	0.0036	1	0.0071	0.0597	1	0.0071	0.4248	6	0.025
Mating system	Song rep. size	0.02	3	0.01	0.063	2	0.0083	0.1478	4	0.0125
Mating system	Song duration	0.649	6	0.025	0.899	7	0.05	0.0075	2	0.0083
Mating system	Intersong interval	0.651	7	0.05	0.579	6	0.025	0.4881	7	0.05
Mating system	Song rate	0.0199	2	0.0083	0.0698	3	0.01	0.0266	3	0.01
Mating system	Song continuity	0.1199	4	0.0125	0.221	4	0.0125	0.3985	5	0.0167
EPP	Syllable rep. size	0.0014	1	0.0071	0.001	1	0.0071	0.1156	1	0.0071
EPP	Syllables per song	0.019	2	0.0083	0.02	2	0.0083	0.2764	6	0.025
EPP	Song rep. size	0.547	6	0.025	0.566	6	0.025	0.3792	4	0.0125
EPP	Song duration	0.115	5	0.0167	0.329	5	0.0167	0.1590	2	0.0083
EPP	Intersong interval	0.112	4	0.0125	0.045	3	0.01	0.3650	3	0.01
EPP	Song rate	0.932	7	0.05	0.714	7	0.05	0.5785	7	0.05
EPP	Song continuity	0.086	3	0.01	0.052	4	0.0125	0.3898	5	0.0167

Supplementary Table 2. Testing for significance while controlling for multiple hypothesis testing. The Holm sequential Bonferroni procedure (Holm 1979) is used to correct for multiple tests by setting a more stringent p-value threshold (α) to determine significance, using the following formula: $\alpha_{corrected} = \alpha / (n - R + 1)$, where α is set to 0.05, n is the number of tests performed, and R is the rank number of the uncorrected p-value by degree of significance. Green cells indicate p-values that passed this corrected threshold.

	Polygyny	EPP
Syllrep	0.246	0.0015
Syllsong	0.0575	0.009
Song	0.052	0.481
Duration	0.966	0.26
Interval	0.579	0.0455
Rate	0.0515	0.5915
Continuity	0.1985	0.0385

Supplementary Table 3. P-values from PhylANOVA analysis using consensus tree containing only species with genetic data. This test produced consistent results to the analysis in the main text: EPP + Syllrep remains significant. EPP + Syllsong was not significant after correcting for multiple hypothesis testing.

Syllrep		monoHEPP	monoLEPP	polyHEPP	polyLEPP
	monoHEPP	1	0.292	0.957	**0.045**
	monoLEPP	0.292	1	0.957	0.957
	polyHEPP	0.957	0.957	1	0.215
	polyLEPP	**0.045**	0.957	0.215	1
Syllsong		monoHEPP	monoLEPP	polyHEPP	polyLEPP
	monoHEPP	1	0.586	1	0.225
	monoLEPP	0.586	1	1	1
	polyHEPP	1	1	1	0.455
	polyLEPP	0.225	1	0.455	1
Song		monoHEPP	monoLEPP	polyHEPP	polyLEPP
	monoHEPP	1	1	0.63	0.555
	monoLEPP	1	1	1	1
	polyHEPP	0.63	1	1	1
	polyLEPP	0.555	1	1	1
Duration		monoHEPP	monoLEPP	polyHEPP	polyLEPP
	monoHEPP	1	1	1	0.444
	monoLEPP	1	1	1	0.7625
	polyHEPP	1	1	1	1
	polyLEPP	0.444	0.7625	1	1
Interval		monoHEPP	monoLEPP	polyHEPP	polyLEPP
	monoHEPP	1	0.8745	0.8745	0.722
	monoLEPP	0.8745	1	0.5275	0.8745
	polyHEPP	0.8745	0.5275	1	0.24
	polyLEPP	0.722	0.8745	0.24	1
Rate		monoHEPP	monoLEPP	polyHEPP	polyLEPP
	monoHEPP	1	0.687	0.15	0.15
	monoLEPP	0.687	1	0.1475	0.138
	polyHEPP	0.15	0.1475	1	0.687
	polyLEPP	0.15	0.138	0.687	1
Continuity		monoHEPP	monoLEPP	polyHEPP	polyLEPP
	monoHEPP	1	1	1	0.153
	monoLEPP	1	1	1	0.736
	polyHEPP	1	1	1	0.153
	polyLEPP	0.153	0.736	0.153	1

Supplementary Table 4. P-values of pairwise comparisons in phylANOVA test of interaction between EPP and mating system. monoLEPP = monogamy + low EPP, monoHEPP = monogamy + high EPP, polyLEPP = polygyny + low EPP, polyHEPP = polygyny + high EPP. The comparison of syllable repertoires between monoHEPP and polyLEPP is weakly significant (PhylANOVA $p = 0.045$). No other pairwise comparisons are significant.

		PGLS				GLMM
		Value	Std.Error	t-value	p-value	pMCMC
Syllrep	(Intercept)	1.585541	0.258613	6.13094	1.71E-07	0.001
	Final.polygyny	-0.33611	0.238977	-1.40647	0.166164	0.628
	Final.EPP	-0.68211	0.170692	-3.99616	**0.000226**	**0.001**
	Final.polygyny:Final.EPP	0.264461	0.297073	0.890222	0.377881	0.718
Syllsong	(Intercept)	1.585541	0.258613	6.13094	1.71E-07	0.001
	Final.polygyny	-0.33611	0.238977	-1.40647	0.166164	0.878
	Final.EPP	-0.68211	0.170692	-3.99616	**0.000226**	0.088
	Final.polygyny:Final.EPP	0.264461	0.297073	0.890222	0.377881	0.864
Song	(Intercept)	1.295992	0.455544	2.84493	0.006228	0.001
	Final.polygyny	-0.33823	0.356245	-0.94943	0.346556	0.656
	Final.EPP	-0.25149	0.277228	-0.90715	0.368283	0.444
	Final.polygyny:Final.EPP	0.322242	0.457492	0.704366	0.484177	0.554
Duration	(Intercept)	0.284452	0.268699	1.058626	0.294759	0.194
	Final.polygyny	0.099862	0.202116	0.494082	0.623369	0.516
	Final.EPP	-0.16471	0.149461	-1.10203	0.275622	0.474
	Final.polygyny:Final.EPP	0.034229	0.254048	0.134736	0.893351	0.998
Interval	(Intercept)	0.522349	0.273347	1.910937	0.063578	0.074
	Final.polygyny	-0.07013	0.234609	-0.29893	0.766619	0.324
	Final.EPP	-0.01773	0.185171	-0.09577	0.924205	0.71
	Final.polygyny:Final.EPP	0.238115	0.279967	0.85051	0.400369	0.188
Rate	(Intercept)	1.028859	0.208494	4.934706	1.63E-05	0.001
	Final.polygyny	-0.20031	0.178947	-1.11935	0.270015	0.018
	Final.EPP	0.040323	0.141239	0.285496	0.776815	0.29
	Final.polygyny:Final.EPP	0.060147	0.213544	0.281661	0.779732	0.276
Continuity	(Intercept)	-0.53069	0.177448	-2.9907	0.004865	0.002
	Final.polygyny	0.051889	0.1523	0.340703	0.735203	0.668
	Final.EPP	0.007152	0.120207	0.059498	0.952867	0.938
	Final.polygyny:Final.EPP	-0.13627	0.181745	-0.74976	0.458014	0.418

Supplementary Table 5. Results of PGLS and GLMM. Results are shown for the intercept, effects of mating system alone, effects of EPP alone, and interacting effects of mating system and EPP. Significant findings for the fixed effects are bolded.
