# Supplementary material for: Polygyny is linked to accelerated birdsong evolution but not to larger song repertoires
Source: Nat Commun. 2019 Feb 21;10:884. doi: 10.1038/s41467-019-08621-3 (PMC6385279; doi:10.1038/s41467-019-08621-3)
Supplement: Supplementary file 3 — Description of Additional Supplementary Files [file 41467_2019_8621_MOESM3_ESM.pdf]

Description of Additional Supplementary Files

File Name: Supplementary Data 1
Description: Full database with references and raw data. The first tab in this worksheet contains the full database of 899 species for which we have raw data for at least one trait out of the mating behaviors and song features we included. The second tab contains the references cited in this database.

File Name: Supplementary Data 2
Description: Jackknife resampling for phlyANOVA tests. We removed each family in turn and repeated the phylANOVA test for the mating parameter and song characteristic specified. We highlight p-values less than 0.05 here to show general trends in the jackknife resampling; for the full analysis in the main text we corrected for multiple hypothesis testing (Table 2, Supplementary Table 2). For full analyses with p-values near 0.05, we observe that removing families from the analysis can tip the scales to either side of this arbitrary threshold, which is not unexpected. For the full analysis that was significant after Holm-Bonferroni correction (EPP+Syllable repertoire), we found similar results with each family removed in turn

File Name: Supplementary Data 3
Description: Jackknife resampling of Brownie analyses. We tested whether song characteristics evolved faster in polygynous versus monogamous lineages or in high versus low EPP lineages. For each analysis, we assessed the robustness of our findings by testing whether the minimum and maximum values of song characteristics from the literature yielded the same results (Min; Max). In addition, we removed each avian family from the analysis in turn and repeated the analyses (Jack). We tested the model that the rate of evolution of each song characteristic was the same in monogamous versus polygynous lineages (Equal rates model, ER) against the model that the rate of evolution of that song characteristic differs between monogamous and polygynous lineages (All rates different model, ARD). We then tested whether the evolution of each song characteristic was faster in monogamous versus polygynous lineages. We repeated these analyses with high versus low EPP.

File Name: Supplementary Data 4
Description: Phylogeny of all species for which we had any song or mating data. We sampled 1000 trees and generated a consensus tree using Phylip. Dots at each node indicate the support for that node: green indicates that all 1000 trees supported the node, yellow 900-999, orange 700-899, red 500-699, dark red

File Name: Supplementary Data 5
Description: Consensus tree in Nexus format. We sampled 1000 trees and generated a consensus tree using Phylip.

File Name: Supplementary Software 1
Description: We provide all data and code used in the manuscript.
