# Supplementary material for: Polygyny is linked to accelerated birdsong evolution but not to larger song repertoires
Source: Nat Commun. 2019 Feb 21;10:884. doi: 10.1038/s41467-019-08621-3 (PMC6385279; doi:10.1038/s41467-019-08621-3)

Supplementary Data 4. Phylogeny of all species for which we had any song or mating data. We sampled 1000 trees and generated a consensus tree with Phylip. Dots at each node indicate the support for that node: green indicates that all 1000 trees supported the node, yellow 900-999, orange 700-899, red 500-699, dark red <500.

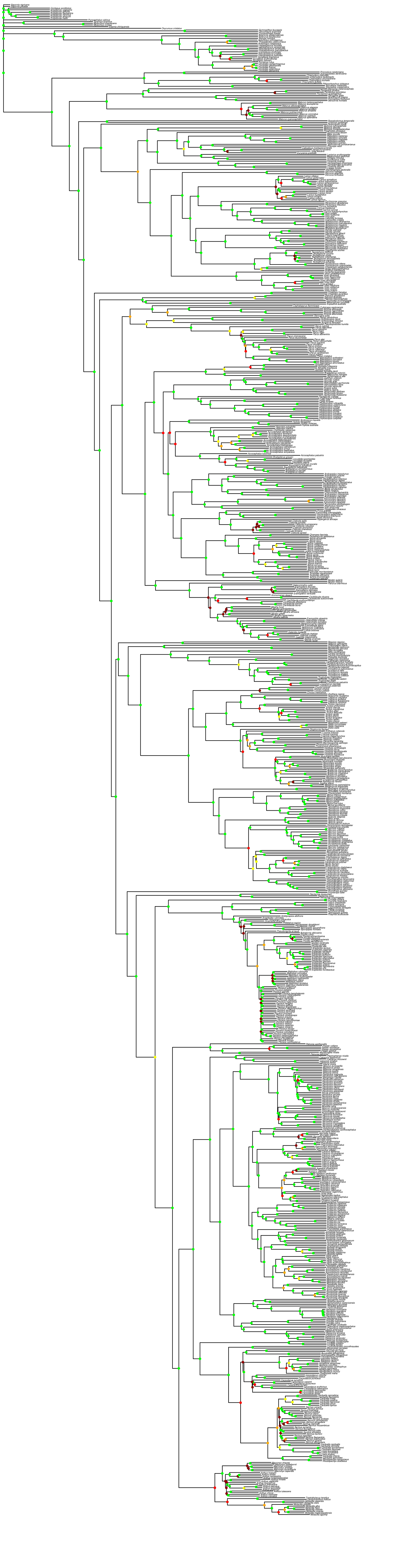

Supplement: Supplementary file 7 — Supplementary Data 4 [file 41467_2019_8621_MOESM7_ESM.pdf]
